# Microbial growth and volatile organic compound (VOC) emissions from carpet and drywall under elevated relative humidity conditions

**DOI:** 10.1186/s40168-021-01158-y

**Published:** 2021-10-19

**Authors:** Sarah R. Haines, Emma C. Hall, Katarzyna Marciniak, Pawel K. Misztal, Allen H. Goldstein, Rachel I. Adams, Karen C. Dannemiller

**Affiliations:** 1 Department of Civil & Mineral Engineering, University of Toronto, Toronto, Ontario M5S 1A4 Canada; 2 Department of Civil, Architectural and Environmental Engineering, University of Texas at Austin, Austin, TX 78712 USA; 3 School of Chemistry, The University of Edinburgh, Edinburgh, EH9 3FJ UK; 4 Department of Environmental Science, Policy and Management, University of California, Berkeley, CA 94720 USA; 5 Department of Plant and Microbial Biology, University of California, Berkeley, CA 94720 USA; 6 Department of Civil, Environmental & Geodetic Engineering, College of Engineering, Ohio State University, Columbus, OH 43210 USA; 7 Division of Environmental Health Sciences, College of Public Health, Ohio State University, Columbus, OH 43210 USA; 8 Sustainability Institute, Ohio State University, Columbus, OH 43210 USA; 9 Department of Civil, Environmental & Geodetic Engineering, Environmental Health Sciences, Ohio State University, 470 Hitchcock Hall, 2070 Neil Ave, Columbus, OH 43210 USA

**Keywords:** Microbiome, Chemistry, Built environment, Carpet, Dust, VOC

## Abstract

**Background:**

Microbes can grow in indoor environments if moisture is available, and we need an improved understanding of how this growth contributes to emissions of microbial volatile organic compounds (mVOCs). The goal of this study was to measure how moisture levels, building material type, collection site, and microbial species composition impact microbial growth and emissions of mVOCs. We subjected two common building materials, drywall, and carpet, to treatments with varying moisture availability and measured microbial communities and mVOC emissions.

**Results:**

Fungal growth occurred in samples at >75% equilibrium relative humidity (ERH) for carpet with dust and >85% ERH for inoculated painted drywall. In addition to incubated relative humidity level, dust sample collection site (adonis *p*=0.001) and material type (drywall, carpet, adonis *p*=0.001) drove fungal and bacterial species composition. Increased relative humidity was associated with decreased microbial species diversity in samples of carpet with dust (adonis *p*= 0.005). Abundant volatile organic compounds (VOCs) that accounted for >1% emissions were likely released from building materials and the dust itself. However, certain mVOCs were associated with microbial growth from carpet with dust such as C_10_H_16_H^+^ (monoterpenes) and C_2_H_6_SH^+^ (dimethyl sulfide and ethanethiol). CO_2_ production from samples of carpet with dust at 95% ERH averaged 5.92 mg hr^-1^ kg^-1^, while the average for carpet without dust at 95% ERH was 2.55 mg hr^-1^ kg^-1^.

**Conclusion:**

Microbial growth and mVOC emissions occur at lower relative humidity in carpet and floor dust compared to drywall, which has important implications for human exposure. Even under elevated relative humidity conditions, the VOC emissions profile is dominated by non-microbial VOCs, although potential mVOCs may dominate odor production.

**Video Abstract**

**Supplementary Information:**

The online version contains supplementary material available at 10.1186/s40168-021-01158-y.

## Background

People spend the majority of their time indoors [1] where they are exposed to a variety of biological and abiotic contaminants. Microbial growth and moldy odors in indoor environments are associated with harmful human health effects [2–5] such as the development of asthma, wheezing and reduced lung function [6–9]. However, the causative agent(s) for these harmful health effects remains unclear [10]. The agent(s) may be a component of microorganisms or the microbial volatile organic compounds (mVOCs) microorganisms release as part of the primary and secondary metabolic processes [11, 12].

Moisture is the critical limiting factor to mold growth indoors [13, 14]. The US Environmental Protection Agency (EPA) recommends that homes maintain a relative humidity lower than 60%, and ideally between 30-50% relative humidity [15]. However, the relative humidity of indoor air does not stay constant and varies with outdoor humidity, occupant density, indoor activities, the air exchange rate, and moisture buffering materials inside [16, 17].

Microbes grow in carpet dust and gypsum drywall under elevated (>80%) relative humidity conditions [18–20]. Microbial growth in both these substrates may be sustained for an extended period of time even after the relative humidity in the air has decreased [19, 21]. Drywall and carpet dust differ in moisture uptake. Drywall is quick to absorb moisture and slow to dry [20, 22]. On the other hand, water uptake in dust is more nuanced, as dust mixtures constitute many different particles that may uptake water differently [23–25]. Therefore, the relative humidity in the surrounding air may be sufficient to support fungal growth in building materials, although the specifics of the process likely depend on the substrate.

Moisture may also impact the release of volatile organic compounds (VOCs) of both biological and abiotic origin. Many compounds have been identified as mVOCs resulting from microbial growth on substrates such as drywall and carpet [11]. The specific VOCs produced by microbes can change and are impacted by substrate [26, 27], microbial species type [28, 29] and other parameters (e.g. temperature, pH) which can vary based on the geographic location [11, 30–32]. Geographic location is known to influence the microbiome of the built environment due to differences in outdoor microbial communities impacted by atmosphere, land type, climate and human occupant factors [33, 34]. Different homes contain different microbial communities [19] and housing characteristics such as number of occupants, presence of pets, urbanization level, air conditioner usage among others influence indoor taxonomy [35].

Certain VOCs, such as 1-octen-3-ol and 2-ethyl-1-hexanol, are also known to have both microbial and non-microbial sources [26]. Carpet releases hundreds of VOCs, acting as a primary source of VOCs in the indoor environment [36–40]. Household moisture may also impact the release of VOCs from indoor materials independent of microorganisms [41, 42] because high relative humidity conditions cause polar compounds to be desorbed quickly [43, 44]. For example, the emissions rate of toluene, n-butyl acetate, ethylbenzene and m,p-xylene from wooden floors have been observed to increase when the relative humidity was raised from 50 to 80% [42]. However, we need to better understand how material type and sample location influence microbial growth and mVOCs in different building materials under different relative humidity conditions to ultimately elucidate the impact on the chemistry within a damp home.

The goal of this study is to understand how moisture availability, collection site of dust, and substrate type impact microbial growth and mVOC production. We want to better predict mVOC emissions in regard to microbial growth as well as VOC emissions from a variety of substrates under a range of humidity conditions. Samples of dust, carpet, and drywall were subjected to various relative humidity levels, ranging from 50% - 95% equilibrium relative humidity (ERH), to evaluate differences in microbial growth and VOC/mVOC emissions. The results have implications for understanding and controlling mVOC emissions in damp homes as well as the relationship between health associations and moldy odor.

## Methods

### Sample collection

Nylon cut pile carpet (carpet A) and nylon loop pile carpet (carpet B) were purchased five months prior to sampling incubations. Carpet A contained soil and stain resistant treatments with no antimicrobial treatment while carpet B contained no soil protection but did contain an antimicrobial treatment. The carpets were cut into 5 cm x 5 cm squares, wrapped in aluminum foil, autoclaved for 60 minutes, and then dried overnight at 30°C to sterilize the carpet. Dust was collected from three different parts of the United States: San Francisco Bay Area, CA (three homes), Columbus, OH (three homes) and Gainesville, FL (five homes), from homes without known history of mold and dampness as to not influence the results of our study. Study procedures were reviewed by the University of California IRB, protocol 2018-07-11235. Dust from the same location was mixed together and hand sieved to 250 nm to remove large particles. The dust was homogenized to minimize impacts to the microbial community due to home differences. A subset of the homogenized dust was sent to Steris Corporation (Petaluma, CA) to be ionized with 10 kGy radiation in attempt to create sterilized dust samples. 100 mg of dust was embedded into each pre-autoclaved carpet square using a modified version of the American Society for Testing and Materials (ASTM) method F608-18, in which a 12 cm long 1440 g steel pipe covered in baked aluminum foil was rolled over the carpet squares 30 times.

Gypsum drywall was separated out into two groups (A and B) based on the paint coating: drywall A was painted with low VOC interior latex flat white paint, while drywall B was painted with interior acrylic latex flat wall paint. The drywall was cut into 5 cm x 5 cm squares, wrapped in aluminum foil and autoclaved. The samples were then separated into groups and left to naturally inoculate for four weeks in four separate homes: two in the San Francisco Bay Area, CA (CA 1 and CA 2) and two in Columbus, OH (OH 1 and OH 2). A list of all sample types and characteristics as well as samples used in each experiment type can be found in Table 1 and Table 2 respectively. More information about where the carpet and drywall samples were inoculated can be found in the Online Supplementary Information.

**Table 1:** Descriptions of carpet and drywall types used in sampling

Sample ID	Material Type	Characteristics
Carpet A	Nylon carpet	Cut pile; soil and stain resistant treatment
Carpet B	Nylon carpet	Loop pile; antimicrobial treatment
Drywall A	Gypsum drywall	Low VOC interior latex flat white paint
Drywall B	Gypsum drywall	Interior acrylic latex flat wall paint

**Table 2:** Sample information for the two main experiments: Moisture Availability and Collection Location Samples.

Experiment Name	Description
**Moisture Availability Samples**	
	Samples incubated at 50%, 65%, 70%, 75%, 80%, 85% and 95% ERH for 4 weeks at 25°C. Chemical emissions were measured using the flow-through chamber approach in which emissions were measured for a total of 2 hours**.**
** Carpet**	
	Carpet A sterilized (control)
	Carpet A with CA dust
** Drywall**	
	Painted Drywall A sterilized (control)
	Painted Drywall A inoculated in CA home 1
**Collection Location Samples**	
	Duplicates of each sample type were incubated at 50% and 85% ERH for 4 weeks at ambient room temperature. Chemical emissions were measured using a direct sampling approach in which the sampling line for the volatile chemistry instrument was inserted into the parafilm covering the jar of an individual sample.
** Carpet**	
	Carpet A sterile (control)
	Carpet B sterile (control)
	Carpet A with CA dust
	Carpet A with OH dust
	Carpet A with FL dust
	Carpet A with irradiated CA dust (control)
	Carpet A with irradiated OH dust (control)
	Carpet A with irradiated FL dust (control)
	Carpet B with CA dust
	Carpet B with OH dust
	Carpet B with FL dust
** Dust**	
	Irradiated CA dust (control)
	Irradiated OH dust (control)
	Irradiated FL dust (control)
	Original collected non-incubated dust from CA
	Original collected non-incubated dust from OH
	Original collected non-incubated dust from CA
	Painted Drywall A in OH home 1
	Painted Drywall B in OH home 1
	Painted Drywall A in OH home 2
** Drywall**	
	Painted Drywall B in OH home 2
	Painted Drywall A in CA home 1
	Painted Drywall B in CA home 1
	Painted Drywall A in CA home 2
	Painted Drywall B in CA home 2

### Moisture availability incubations

The first set of experiments was designed to determine the relative humidity inflection point, or the relative humidity level at which microbial growth and microbially-mediated chemical emissions increases in the different materials. We incubated both carpets embedded with dust and drywall at ERH values of 50%, 65%, 70%, 75%, 80%, 85% and 95% for four weeks. ERH is the relative humidity of the sealed headspace above the material (carpet, dust drywall). At equilibrium conditions the water activity and relative humidity are equal, however the water activity of building materials cannot be measured [45]. Therefore, we measured the equilibrium relative humidity and utilized salt solutions to simulate these ERH conditions [19]. To create water activity levels of 0.50, 0.65, 0.70, 0.75, 0.80, 0.85 and 0.95, salt solutions were made using NaCl and MgCl_2_ [18]. The water activity of the salt solutions was tested for accuracy using an AquaLab™ PawKit Water Activity Meter (Decagon 125 Devices, Pullman, WA, USA). For this sample set, San Francisco Bay Area, CA dust embedded into carpet A and inoculated drywall A in CA home A were utilized and incubated at each of the ERH conditions. Samples of autoclaved “non-microbial” carpet A and drywall A were incubated as controls. The samples were placed into 1 L incubation jars, with triplicates in each jar and incubated for ~4 weeks at 25°C.

When collecting dust from the samples of carpet with dust incubated at 95% ERH, one challenge was how to prevent excessive moisture accumulation in the carpet. For instance, while incubating samples at 95% ERH, water had condensed on the sides and bottom of the incubation jar resulting in visibly moist carpet. Therefore, the dust was not able to be efficiently vacuumed due to excessive moisture in the carpet. While growth was visible on the carpet, only a low quantity of dust was collected on the filter and estimates of biomass through qPCR were not possible. We incubated new samples of carpet A embedded with CA dust at 95% ERH for four weeks in a larger open mouth jar to allow for air to pass efficiently through the parafilm and prevent a visibly moist carpet. This new sample was used for qPCR microbial quantification analysis and the original sample was used for species composition.

### Collection location

Samples from the different building materials (carpet A, carpet B, drywall A, drywall B) as well as the homogenized dust samples that were collected in CA, OH, or FL, were utilized to determine variations in microbial growth, mVOC, and VOC emissions (Table 2). Each sample type was incubated in duplicate in separate glass jars covered with parafilm. Samples were incubated at either 50% ERH or 85% ERH for four weeks at room temperature using the same method described previously. Once the incubations were complete, VOC/mVOC emissions were sampled using a direct sampling approach.

### VOC measurement

VOCs were measured using an Ionicon proton-transfer-reaction time-of-flight mass spectrometer (PTR-TOF-MS), a new system that allows for VOC measurements at high time (<1 s) and mass (>5000 d/dm) resolution, high sensitivity and an ultra-low detection limit [46]. The PTR-TOF-MS (PTR-TOF 8000, IONICON Analytik GmbH) detects VOCs in real-time through a proton transfer reaction that takes place between a sample gas and the produced H_3_O^+^ ions form the ion source [46]. It records the mass spectrum as a mass to charge ratio (m/z) of typically 10.000-500.000 at a rate of 0.1 - 10 Hz, using the primary ion reagent H_3_O^+^ [27, 47]. In our experiments, the time resolution was set at 5-s consistently. A multi-port flow-through valve system in conjunction with a flow-through multi-chamber approach was used to measure up to eight samples in a 60-minute cycle, with each sample chamber being individually measured for 7.5 minutes. The chambers made of glass and VOC-compatible materials (Teflon® lid) were continually flushed throughout the experiments at 0.2 L/min of zero-air generated by the Ultra High Purity Zero Air Generator (ZAG) (Aadco, Cleves, OH, USA), which was connected to a glass bubbler to produce the desired ERH condition throughout the chambers. The ZAG produces VOC free air that contains ultra-low concentration of impurities and CO_2_ ensuring that the background concentration in the air is zero. The sample chambers were connected to both the humidity controlled zero air supply and the PTR-TOF-MS by 1.6 mm (1/16”) polyether ether ketone (PEEK) tubing. A negative control chamber was included in each set of samples to measure the baseline emission levels from the instrument setup to account for the concentration and emission rates calculations. A similar setup was used in Misztal (2018) and a diagram of the experimental setup can be found in the Supporting Information in the referenced manuscript [27].

The moisture availability samples were sampled using the flow-through chamber approach, in which a sample was placed in one of seven chambers. An eighth jar was used as a negative control. Each chamber was individually sampled twice for 7.5 minutes each time, leading to an overall sampling time of two hours. For the collection location samples, a direct approach to sampling was applied, as the sampling line for the PTR-TOF-MS was inserted into the parafilm covering the chamber of an individual sample. The sampling time was also significantly shorter than the moisture availability samples, as each collection location sample was only sampled for two to three minutes total.

Calibrations were performed using a gas mixture that contained compounds representative for both microbial and non-microbial sources. The gas mixture was prepared by Apel-Reimer (Miami, FL, USA) and has a guaranteed +/- 5% accuracy [48]. The gas standard was composed of compounds detectable at the following m/z ratios, representative of the protonated parent compounds: 33.034, 47.05, 69.034, 59.05, 63.027, 85.029,93.071, 97.029, 118.066, 145.159, 153.128, 155.09, 205.196, 234.876, 371.102. Some semi volatile compounds (e.g. nonanal, indole, citral) with very low vapor pressures were not included in sensitivity and transmission calculations due to potential losses. CO_2_ and H_2_O concentrations within each chamber were measured for each experiment using a Licor LI-840. The measurements for CO2 (ppm) and H_2_O (‱) were taken at a 1-s time resolution.

### Microbial analysis.

#### DNA extraction and sequencing

Each carpet square was vacuumed, and dust was collected using a sterile 37mm Air/Liquid Sampling Cassette 3-Piece w/0.45um Gridded MCE Filter (Zeflon International, FL USA) connected to the laboratory vacuum system at a flow rate of 47 L/min. To prepare for DNA extractions, the collected dust was weighed into 50 mg aliquots and placed in 2.0 mL screw top vials. To collect microbes from the drywall samples, the top of the painted drywall was swabbed with a Puritan® Sterile Cotton Tipped Applicator that had been dipped in Tris-EDTA. The swab tip was then cut and placed in a 2.0 mL screw top vial.

DNA was extracted and processed for qPCR and amplicon sequencing following the protocols described in Haines (2020)[19]. Sequence data was submitted to the European Nucleotide Archive under accession number PRJEB41403. Detailed methods regarding qPCR and DNA sequencing analysis can be found in the Supplementary Information.

### Chemical analysis

The raw data from the PTR-TOF-MS was pre-processed using PTRwid [49] and MATLAB® [50]. General process routines included trimming and removal of poor-quality data, subtraction of Zero Air, processing calibrations and applying sensitivities.

The emission rates (μg hr^-1^) were calculated from the corresponding concentration data (ppb) for the moisture availability samples. The emissions factor (μg hr^-1^ g^-1^) was calculated by dividing the emissions rate by the weight of the carpet sample. Pre-experimental runs on clean empty chambers showed consistent and small trace backgrounds of VOCs which were typically zero or close to zero (typically several orders of magnitude lower than those measured from the samples). In addition, the negative control that was included in each group of samples was used to subtract potential trace emissions from the non-sample sources (chambers, petri dishes, or any other chemical background traces of emissions in the system set-up). Emissions were calculated using the formula that is outlined in the Supplementary Information. The collection location samples were measured using a direct headspace sampling approach instead of the flow-through chamber approach. This allows for a large volume of samples to be processed in a short duration; however, it limits the interpretation of the data to a semiquantitative approach. Therefore, no emission rates were calculated for the collection location samples, and instead the concentration fingerprints were examined for a variety of samples and translated into a categorical representation of either “present” or “not present.”

There is high confidence in the assigned chemical formulas and in the formulas determined using the protonated mass. However, the exact compound structure the formula represents in multi-isomer cases cannot be structurally speciated. A “compound consistent with chemical formula” was appointed to most chemical formulas based on both the properties of the specific compound (e.g. vapor pressure, boiling point, known sources) and on the properties of the sample itself. Expert opinion was utilized when selecting these consistent compounds to ensure that the most accurate conclusion was drawn. All chemical formulas are represented with the chemical ionization method of that ion, which in the majority of cases is a proton transfer reaction resulting in H^+^ addition to the formula. A more detailed description of both the process of formula identification and compound identification are contained in the Supplementary Information.

### Statistical analysis

Statistical Analysis System (SAS), version 9.4 (SAS Institute, Inc) was utilized to determine comparisons within the taxonomy and chemical emissions for the moisture availability samples. The average emissions were found across the entire mass range for each individual sample replicate, and three replicates were used for each sample group. A table with all of the statistical tests performed can be found in Supplemental Information Table S1. Comparisons were made between emission rates from different samples type (carpet, dust, drywall) and humidity levels. The MULTTEST Procedure with the false discovery rate (FDR) adjustment [51], was utilized to determine the statistical significance (*p* <0.05) of different variables impacting the taxonomy of samples as well as the significance of chemical emissions at different humidity levels. The FDR adjustment uses the linear step-up method that is described in Benjamini and Hochberg (1995), in which the p-value may control the false discovery rate. Each data set was normalized using the inverse hyperbolic sine (IHS) transformation, which, unlike a logarithmic transformation, allows for values <1 and 0 [18, 19, 52, 53].

For fungi and bacteria, only species that were detected in ≥20% of samples in each sample set were included in the statistical analysis. For the “moisture availability samples” sample sets were separated by carpet and drywall in which species found in ≥20% of carpet A with CA home 1 dust (328 fungal species and 156 bacterial species) were utilized for the carpet analysis and species found in ≥20% of painted drywall A inoculated in CA home 1 (235 fungal species and 53 bacterial species) were utilized for the drywall analysis. For the “collection location samples”, as comparisons were made between collection locations, sample type and relative humidity, only species found in ≥20% of all collection location samples (582 fungal species and 157 bacterial species) were utilized in the statistical analysis. In QIIME, the adonis method was used to determine the statistical significance of taxonomic sample groupings from the beta diversity distance matrices, Bray-Curtis for fungi and Unifrac for bacteria. Relative humidity groupings based on species composition in carpet with dust samples and inoculated drywall samples were first observed by creating large heat maps using the quantity of each species and sorting by abundance. Using these visual cues from the heat maps, we tested the significance of the groupings of species based on relative humidity level through adonis in QIIME. Adonis was also used on the chemical emissions data in RStudio using Euclidean distances [54]. The statistical tests explored the role of relative humidity, sample type, and collection site on microbial and chemical emissions.

## Results

### Determination of relative humidity level required to support growth

**Fig. 1:**
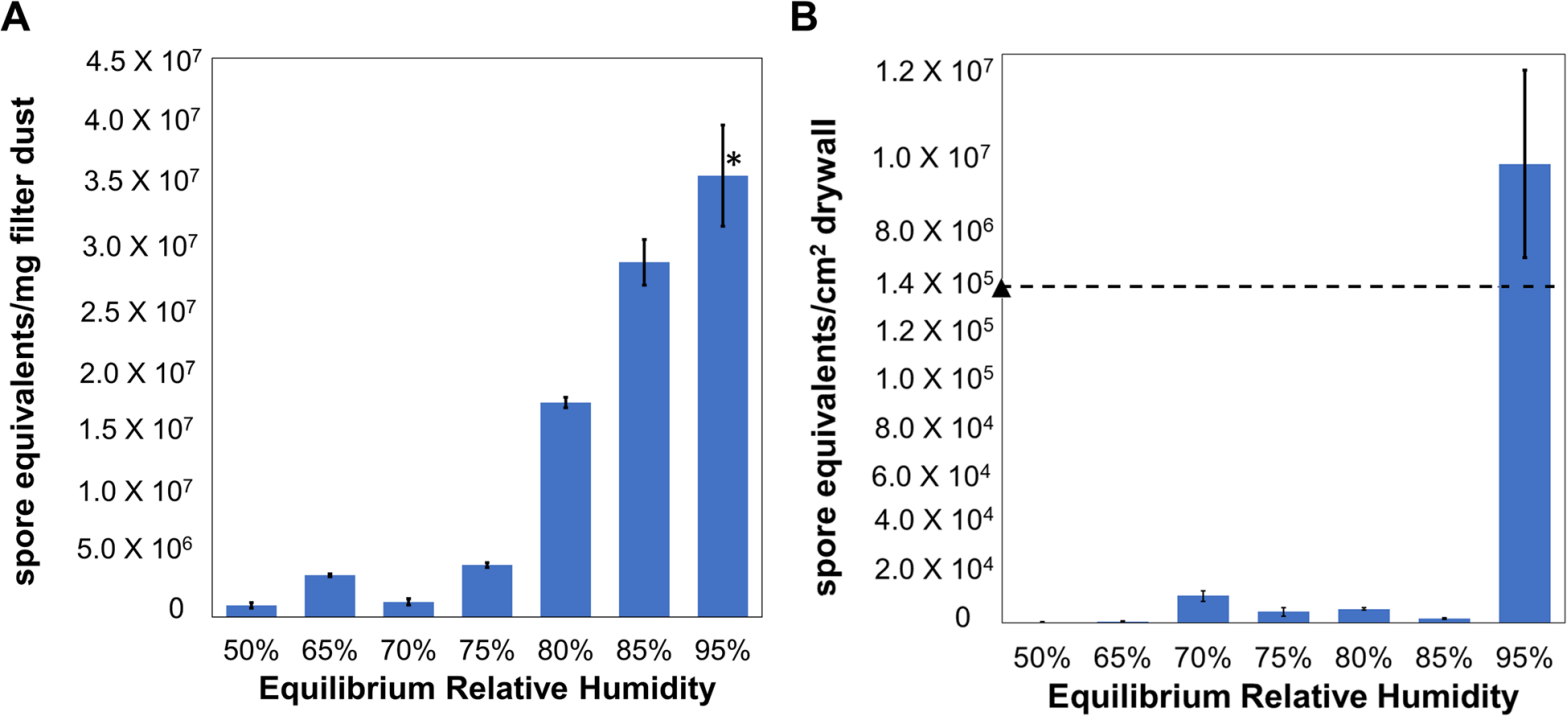
Fungal quantity from qPCR of the CA dust embedded in carpet A samples and drywall taken from qPCR of the CA inoculated drywall samples. **A** Fungal growth in CA carpet A with dust revealed an inflection point between 75% to 80% ERH. **B** Fungal growth in CA painted drywall A revealed an inflection point between 85% to 95% ERH. *New samples of dust and carpet were incubated at 95% ERH for 4 weeks continuously at a later date to determine accurate quantities of fungal growth. Original samples were inconclusive at 95% ERH as the carpet was too wet to collect adequate dust.

Fungal growth was measured in carpet samples with CA dust and painted drywall inoculated in CA home 1 at different RH levels. Growth in CA carpet A with dust was determined to occur somewhere between 75% ERH and 80% ERH, while growth in drywall was observed to occur between 85% ERH and 95% ERH (Fig. 1A and B). *Penicillium* was abundant in both the carpet and drywall samples at 95% ERH, while *Wallemia* and *Aspergillus* were abundant in carpet and *Cladosporium* on drywall. No clear growth patterns were observed for bacteria in these samples (Figure S1).

**Fig. 2:**
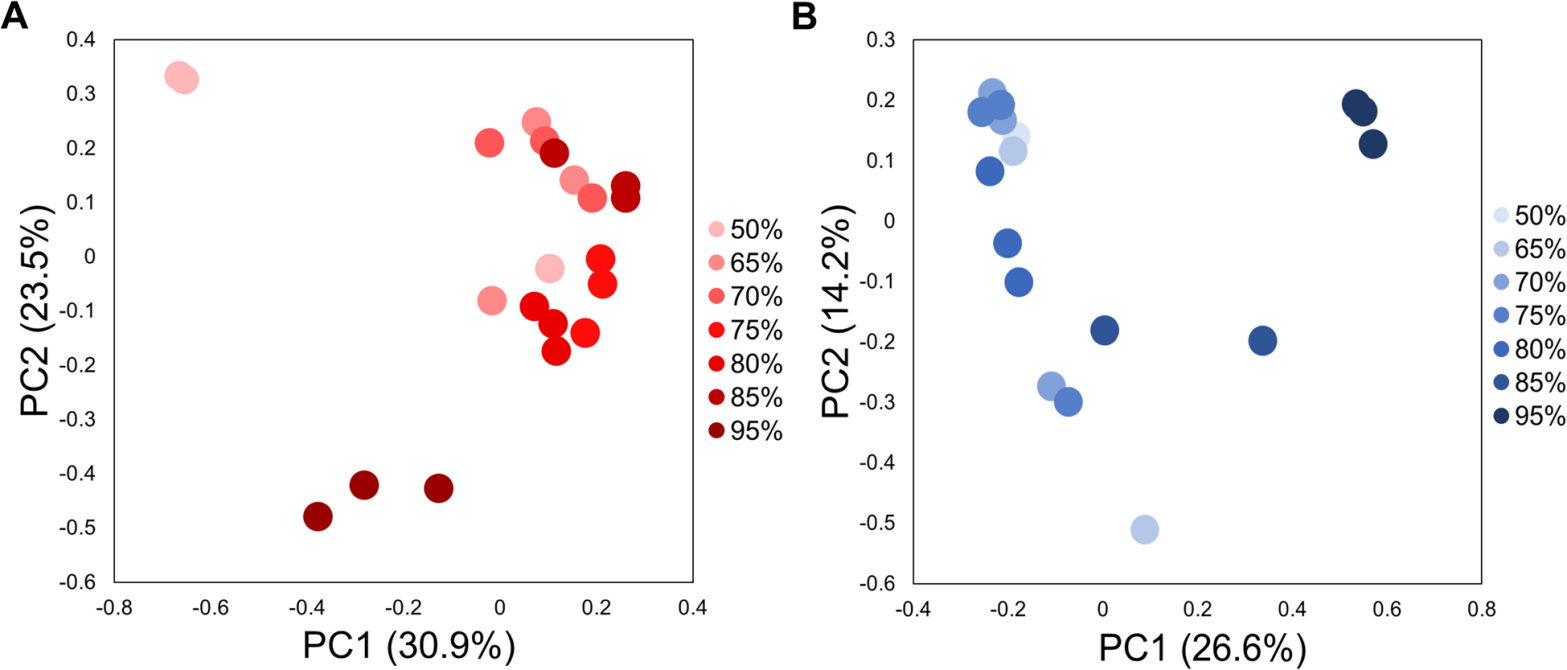
Fungal PCoA plot of the CA dust embedded in carpet A (**A**) and samples of inoculated CA painted drywall A (**B**) incubated at different relative humidity conditions (50%, 65%, 70%, 75%, 80%, 85%, 95%) for 4 weeks.

**Fig. 3:**
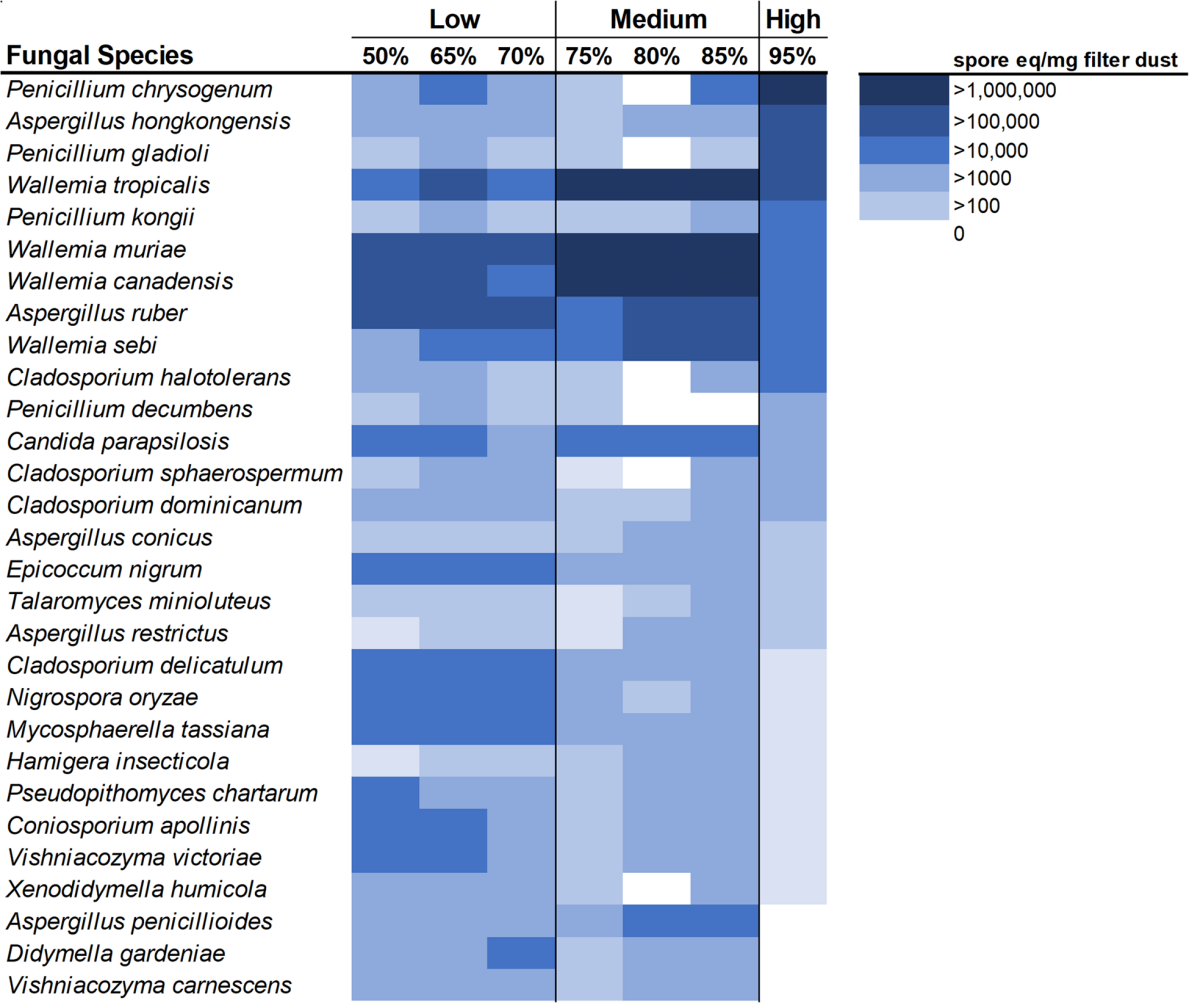
Heatmap of the 29 most abundant species found in at least 70% of the CA carpet with dust samples for the moisture availability tests. An additional heatmap showing fungal species found in at least 20% of all samples can be found in Supplementary Information Figure S2. Species statistically separated into three categories, Low (50%-70%), Medium (75%-85%) and High (95%) (*R*^*2*^=0.18, *p*=0.001).

Fungal communities statistically significantly varied by ERH condition in both carpet and drywall (Fig. 2A, adonis R^2^ = 0.17, *p*=0.003, Fig. 2B, adonis *R*^*2*^=0.16, *p*= 0.002 respectively). The fungal species composition of carpet with CA dust were statistically associated with three distinct groups based on relative humidity condition: Low ERH = 50%, 65% and 70%, Medium ERH = 75%, 80% and 85% and High ERH = 95% (*R*^*2*^=0.18, *p*=0.001)(Fig. 3, Figure S2 & S3). Species within the genera *Penicillium, Aspergillus, Alternaria*, and *Cladosporium* were associated with the High ERH condition when compared to the Medium and Low ERH condition (Table S2), while *Wallemia* species were more associated with the Medium ERH condition than Low ERH (Table S3). Many species were more statistically significantly associated with the Low ERH condition than the High and Medium conditions (Table S4).

**Fig. 4:**
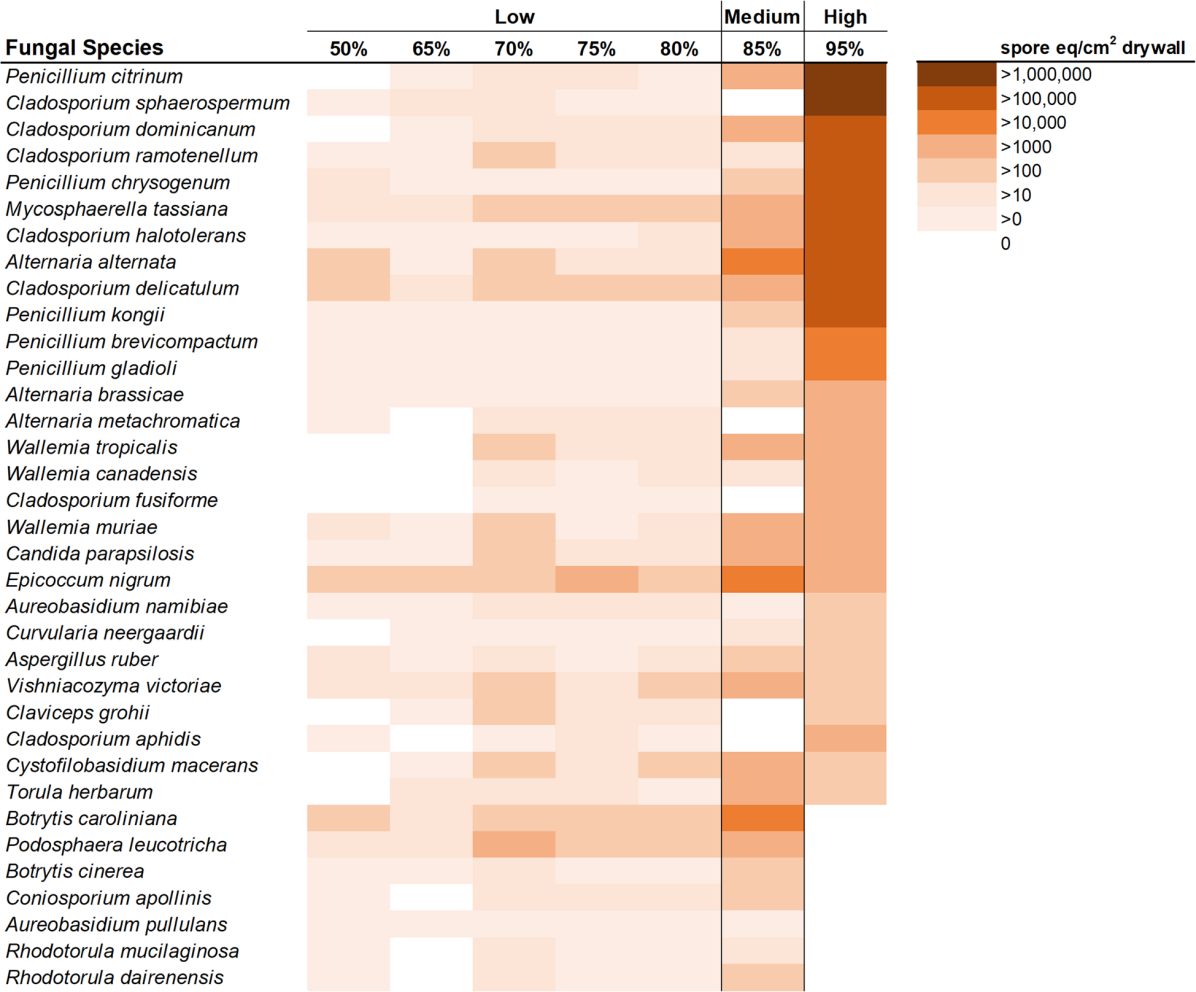
Heatmap of the 35 most abundant fungal species found in at least 60% of all CA painted drywall A samples in the moisture availability tests. Additional heatmap with fungal species found in at least 20% of all samples can be found in Supplementary Information Figure S3. Species statistically separated into three categories based on relative humidity 50%-80% ERH (Low), 85% ERH (Medium) and 95% ERH (High) (*R*^*2*^=0.25, *p*=0.001).

The adonis statistical analysis revealed that the fungal communities on the painted drywall samples also grouped into three categories based on relative humidity, although the cutoff values for the groups differed from carpet: 50%-80% ERH (Low), 85% ERH (Medium) and 95% ERH (High) (*R*^*2*^=0.25, *p*=0.001)(Fig. 4 & S4). *Cladosporium, Penicillium* and *Alternaria* species were more associated with 95% ERH when compared to samples incubated at 50%-80% ERH or 85% ERH (Table S5). *Penicillium kongii, Penicillium citrinum, Penicillium phoeniceum, Aspergillus clavatus,* and *Aspergillus carbonarius* were all associated with the 85% ERH condition compared to the 50%-80% ERH condition (Table S6).

**Fig. 5:**
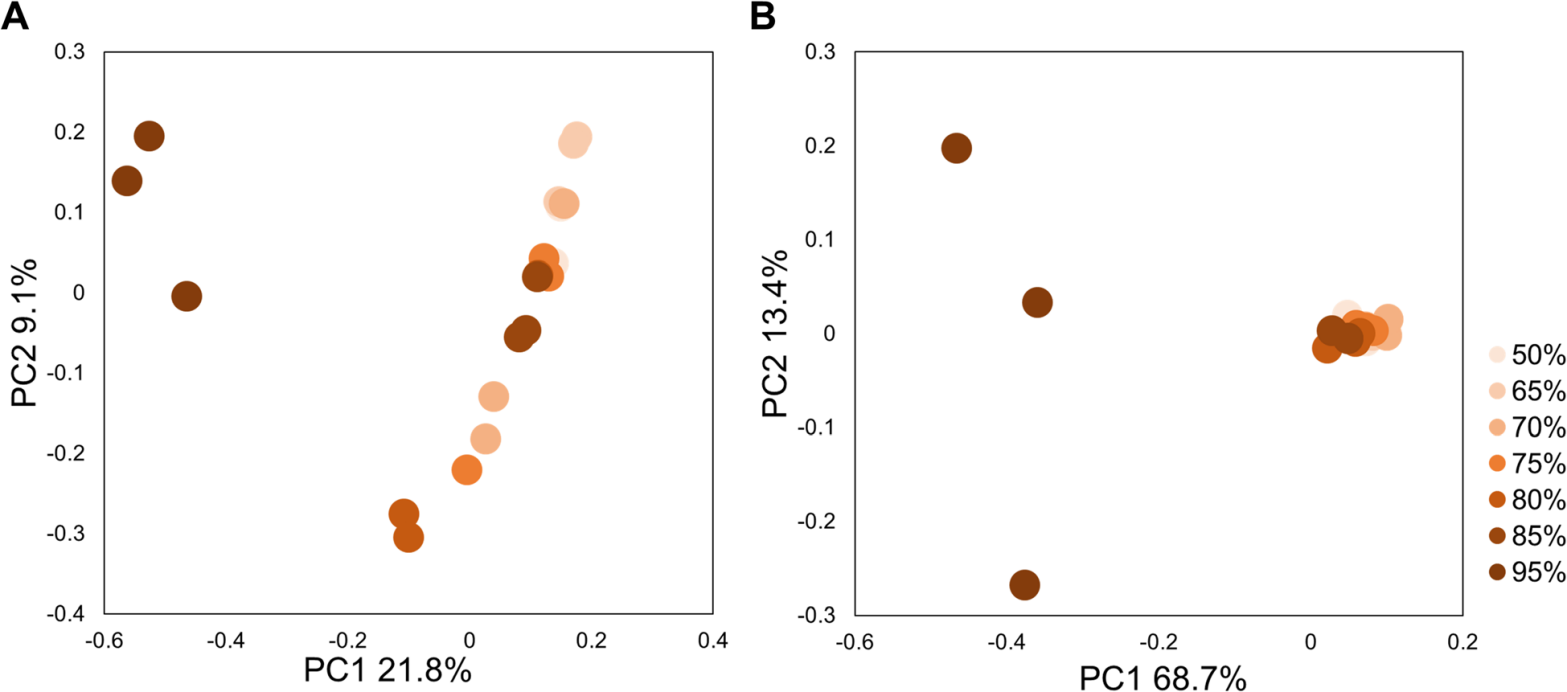
Bacterial PCoA, using the unweighted unifrac (**A**) and weighted unifrac (**B**) distance indices, of the carpet with CA dust from the moisture availability experiments incubated at 50%, 65%, 70%, 75%, 80%, 85% and 95% ERH.

For the carpet and dust samples, the bacterial communities statistically significantly separated by relative humidity condition (Unweighted Unifrac R^2^ = 0.13, *p*=0.001 & Weighted Unifrac R^2^ = 0.31, *p*=0.002)(Fig. 5A and B). Bacterial communities in carpet samples with CA dust statistically separated into four groups based on relative humidity: 50%-65%, 70%-75%, 80%-85%, and 95% (Unweighted Unifrac R^2^ = 0.15, *p*=0.001 & Weighted Unifrac R^2^ = 0.36, *p*=0.001) (Figure S6). Certain bacterial species, such as *Staphylococcus aureus*, *Serratia marcescens*, *Pseudomonas viridiflava*, *Acinetobacter johnsonii,* and *Janthinobacterium lividum*, among others, were more associated with 50%-85% when compared to 95% ERH (Table S7). *Streptococcus, Staphylococcus, Sphingomonas*, *Corynebacterium* and *Pseudomonas* were the most abundant bacterial genera found in carpet with CA dust (Figure S6).

Similar to the dust embedded in carpet samples, the abundant genera in the painted inoculated drywall consisted of *Corynebacterium, Sphingomonas, Staphylococcus* and *Streptococcus* (Figure S7). In painted drywall A, *Rothia mucilaginosa* and *Methylobacterium adhaesivum* were found in abundance in the 95% ERH samples (Figure S7). In fact, each ERH condition contained different abundant bacterial species in the drywall samples when compared across ERH incubation (Table S8).

### Microbial diversity associated with material type and collection location

**Fig. 6:**
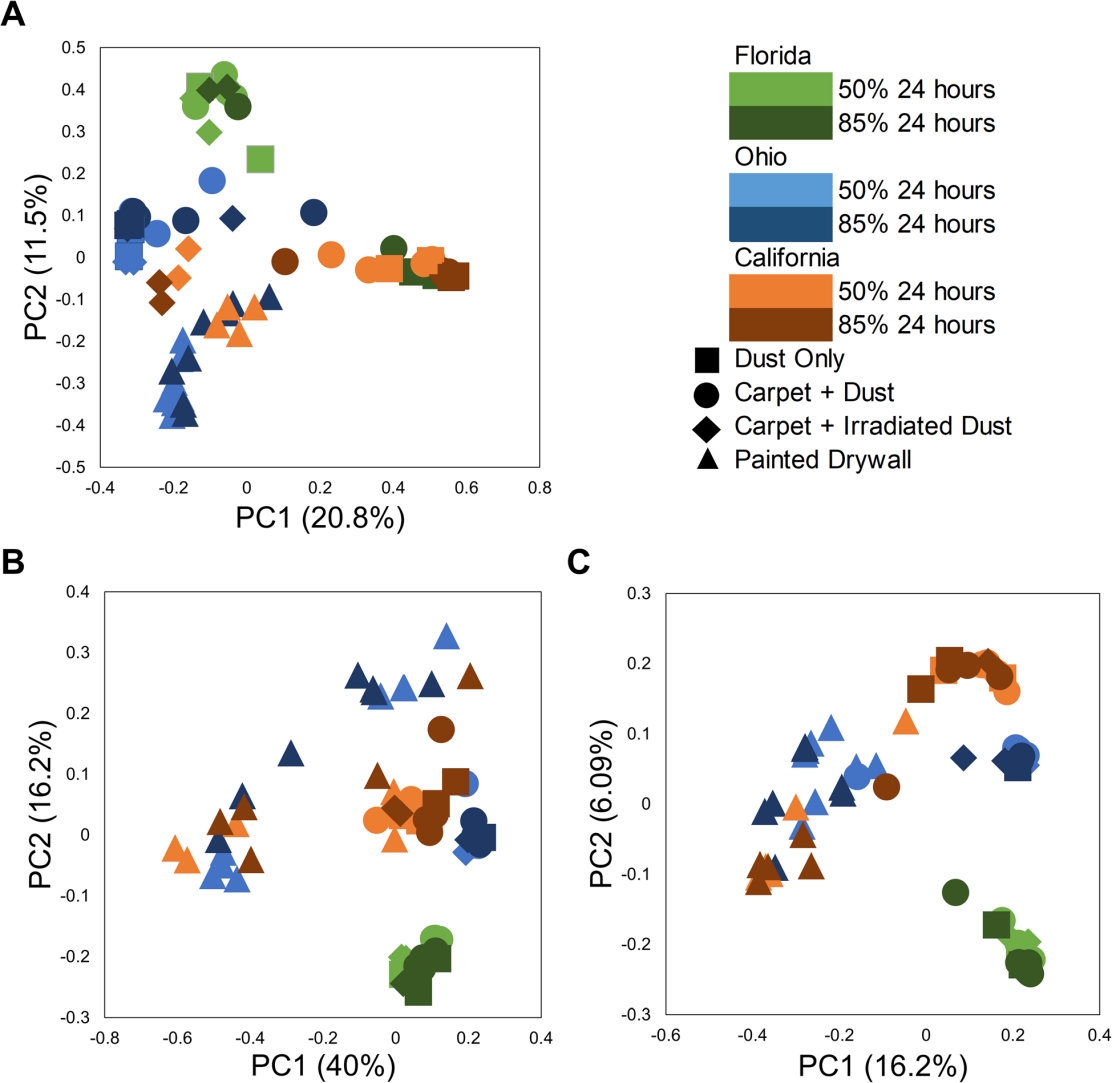
Principle coordinate analysis (PCoA) of all collection location samples that were incubated at either 50% ERH or 85% ERH. **A** Fungal Bray-Curtis analysis, **B** Bacterial Weighted Unifrac and **C** Bacterial Unweighted Unifrac where sample separated due to collection site (Florida, Ohio or California) as well as material type.

Composition of fungal and bacterial communities statistically differed based on the material type and collection site (Table S9). Fungal and bacterial communities were different in carpet compared to drywall samples (Bray-Curtis, adonis *R*^*2*^ = 0.26, *p*=0.001)(Figure S8), Unweighted Unifrac R^2^ = 0.23, *p*=0.001 & Weighted Unifrac, adonis *R*^*2*^ = 0.48, *p*=0.001)(Figure S9). When comparing material type, carpet was associated with higher concentrations of 411 fungal species, while drywall was only associated with higher concentrations of nine fungal species (Table S10). Within a material sample type, the location of the collection influenced the composition of both fungal and bacterial communities (Fig. 6 & Table S9). Different fungal species were determined to be abundant at 85% ERH for each location, FL, CA and OH (Figure S10, Figure S11 & Figure S12). Fungal species associated with a specific location of collection site when compared to the other locations can be found in the Supporting Information (Table S11, S12 & S13).

When comparing relative humidity among all sample types in the moisture availability samples, ERH level was statistically significant for fungal and bacterial growth (Bray-Curtis R^2^ = 0.04, *p*=0.04; Weighted Unifrac R^2^ = 0.05, *p*=0.04; Unweighted Unifrac R^2^ = 0.04, *p*=0.01). However, no statistical taxonomic differences were found when comparing the ERH level (50% or 85%) across all sample types from the collection site samples (Bray-Curtis R^2^ = 0.02, *p*=0.08; Weighted Unifrac R^2^ = 0.005, *p*=0.9; Unweighted Unifrac R^2^ = 0.01, *p*=0.8). Within samples from only CA or only OH, no distinct difference in fungal communities were observed between carpet type or relative humidity condition (50% vs 85%) (Figure S13A & S13B) (*R*^*2*^ = 0.05, *p*=0.86 and R^2^ = 0.08, *p*=0.25 respectively). However, there was a significant difference in fungal communities in carpet with dust samples from FL that were incubated at 50% ERH compared to 85% ERH (Figure S13C)(*R*^*2*^ = 0.22, *p*=0.021), with more species associated with 50% ERH than 85% ERH (Table S14). There was no significant difference in fungal species comparing carpet A to carpet B when grouping all samples from the collection site locations.

### Chemical emissions were dominated by building materials

**Fig. 7:**
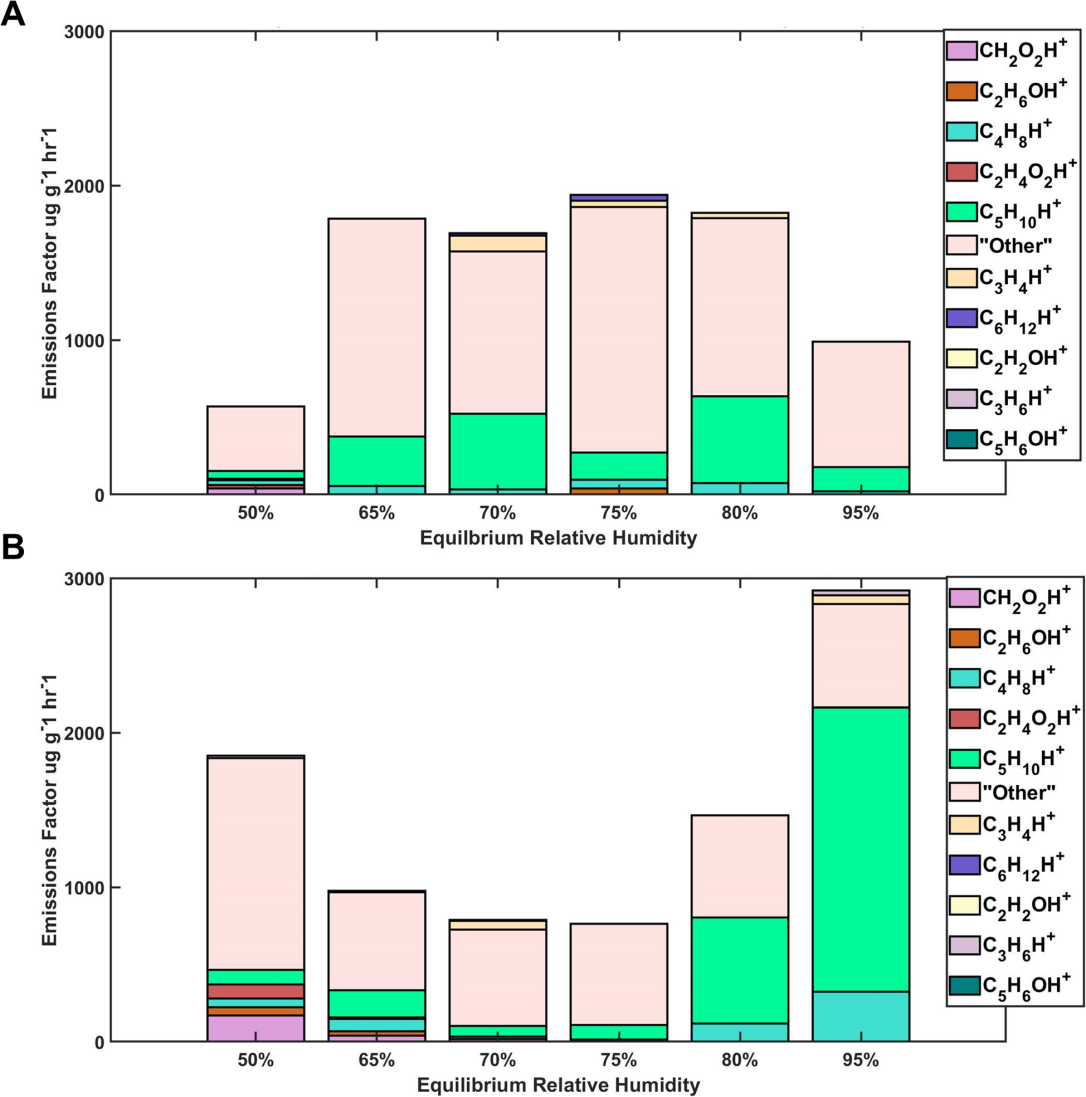
Displaying the emissions factors (μg emission g^-1^ carpet h^-1^ sampling) of abundant (>1%) chemicals emitted from household carpet with dust (**A**) and carpet without dust (**B**) incubated at each ERH condition.

The most abundant VOC emissions (in terms of emissions factor) released from carpet and drywall samples were related to the materials themselves, not microbial compounds. The most abundant (>1% concentration) chemical compounds determined in both carpet without dust samples and dust embedded in carpet samples incubated at 95% ERH were C_5_H_10_H^+^, (cyclopentane/pentene), followed by C_4_H_8_H^+^, (butanol/butene). However, the majority of compounds emitted from the carpet with dust samples contributed to <1% of the total emissions each and were grouped together and classified as “Other” (Fig. 7). Total emissions from carpet with dust samples at 65-80% ERH ranged from ~1750-2000 μg hr^-1^ g^-1^ carpet, while carpet without dust alone samples at 65-80% ERH ranged from ~750-1500 μg hr^-1^ g^-1^ carpet (Fig. 7). Emissions from carpet without dust samples at 95% ERH emitted more C_5_H_10_H^+^ than the carpet with dust samples at 95% ERH. Chemical compounds C_2_H_4_O_2_H^+^ (acetic acid), CH_2_O_2_H^+^ (formic acid), C_2_H_6_OH^+^ (ethanol), and C_2_H_2_OH^+^ (ketene) were more abundantly emitted from samples incubated at 50% ERH and 65% ERH while C_3_H_4_H^+^ (propyne) appeared in abundance in both the carpet without dust and carpet with dust samples at 70% ERH. C_3_H_6_H^+^ (propene), C_6_H_6_H^+^ (benzene), C_5_H_10_H^+^ (pentene and cyclopentane) and C_3_H_5_NO_2_H^+^ (vinyl carbamate + dehydroalanine), among others were more associated with carpet without dust at 95% ERH than carpet with dust at 95% ERH (Table S15).

The abundant (>1%) chemical families found in carpet without dust incubated at 75-80% ERH consisted of C_x_H_y_O_1_H^+^, C_x_H_y_N_z_O_n_H^+^, C_x_H_y_O_2_H^+^, C_x_H_y_O_3_H^+^, C_x_H_y_H^+^ and S-Containing (sulfur containing) compounds families (Figure S14A). The majority of compounds (92.2%) associated with the carpet without dust incubated at 95% ERH were classified as from the family CxHyH^+^ (Figure S14B). Samples of carpet with dust at 75%-80% ERH and 95% ERH emitted similar chemical families (Figure S15A & S15B). 45.3% of compounds associated with carpet with dust samples incubated at 95% ERH were unidentified while around 12.5% of compounds were classified as S-containing (Figure S15B).

The most abundant compound emitted from the inoculated drywall samples was C_5_H_10_H^+^ (cyclopentane + pentene), which consistently accounts for >10% of the emissions at each ERH level with the exception of the 80% ERH sample. C_4_H_6_O_2_H^+^ (butyrolactone + butenic acid) made up 42.5% of the emissions in the 80% ERH drywall samples. Autoclaved drywall incubated at High 95% ERH was associated with compounds such as CH_2_O_3_H^+^ (performic acid, FDR *p*=0.02), C_2_H_5_NO_2_H^+^ (methyl carbamate, FDR *p*=0.02), C_6_H_6_O_2_H^+^ (2-acetylfuran, FDR *p*=0.02), C_6_H_6_H^+^ (benzene, FDR *p*=0.04), and C_3_H_6_H^+^ (propene, FDR *p*=0.04) among others when compared to inoculated drywall at High 95% ERH (Table S16). Only three mass ratios were associated with the inoculated drywall at High 95% ERH: 77.94 (FDR *p*=0.04), 97.95 (FDR *p*=0.04) and 314.11 (FDR *p*=0.04), though the chemical formula and compound for these masses are undetermined. However, certain compounds that are likely mVOCs such as C_2_H_6_S_2_H^+^ and C_2_H_6_SO_2_H^+^ (dimethyl disulfide and dimethyl sulfone, FDR *p*=0.03), C_9_H_16_H^+^ (nonadiene, FDR *p*=0.04), C_6_H_12_H^+^ (1-hexene, *p*=0.01) and C_4_H_6_O_4_H^+^ (succinic acid, FDR *p*=0.02) were associated with the inoculated drywall at the High ERH condition when compared to the emissions from the inoculated drywall at Low or Medium conditions (Table S17 & Table S18). The emissions rates over the duration of the sampling time were highest during the first 50 hours of sampling and then decreased. An example of the emissions rate for samples of carpet without dust and carpet with dust incubatde at 50% ERH can be found in the Supplementary Information Figure S16. (Compounds listed in parentheses are isomers consistent with the chemical formulas, but other isomers are possible.)

### mVOCs associated with carpet samples

Carpet with dust samples incubated at the High (95% ERH) condition compared to carpet without dust samples incubated at the High (95% ERH) condition were associated with common indoor mVOCs C_10_H_16_H^+^ (monoterpenes, FDR *p*=0.004), C_2_H_6_SH^+^ (dimethyl sulfide + ethanethiol, FDR *p*=0.03) and C_10_H_12_O_2_H^+^ (phenethyl acetate, FDR *p*=0.01) along with other potential mVOCs (Table 3). Carpet with dust samples incubated at the Medium (75%-80% ERH) condition compared to carpet without dust samples incubated at the Medium (75%-80% ERH) condition were associated with probable mVOCs, C_3_H_4_H^+^ (propyne, FDR *p*=0.004), C_7_H_6_OH^+^ (benzaldehyde, FDR *p*=0.04), C_8_H_16_OH^+^ (1-octen-3-ol, FDR *p*=0.0009) and C_3_H_8_OSH^+^ (2(methylmercapto)ethanol, FDR *p*=0.03) (Table S19). No associations were found when comparing dust embedded in carpet samples incubated at the Low ERH condition to carpet without dust incubated at the Low ERH condition.

**Table 3:** The m/z ratios that are associated with carpet with dust samples incubated at 95% ERH when compared to carpet without dust samples incubated at 95% ERH for 4 weeks utilizing the FDR adjustment.

M/z ratios associated with carpet with dust samples at High (95%) ERH				
m/z	Most likely formula	Compounds consistent with chemical formula	Unadjusted ***P***-value	Adjusted ***P***-value
49.028	CH_4_O_2_H^+^	Methanediol	0.0003	0.003
58.029	C_2_H_3_NOH^+^	Methyl Isocyanate	0.003	0.01
63.025	C_2_H_6_SH^+^	Dimethyl sulfide + Ethanethiol	0.009	0.03
74.024	C_2_H_3_NO_2_H^+^	Nitroethene	0.007	0.02
89.04	C_7_H_4_H^+^	Butadiynylallene	0.0002	0.003
91.947	C_2_HClS+	Chloroacetylenethiol	0.002	0.008
93.036	C_3_H_8_SOH^+^	Methylmercaptoethanol	0.008	0.03
103.074	C_5_H_10_O_2_H^+^	Propyl Acetate + Valeric acid	0.01	0.04
115.092	C_7_H_11_FH^+^	Fluoronorbornane	0.01	0.03
117.955	C_3_H_4_S_2_O^+^	Dithiolanone	0.004	0.02
118.903	CHCl_3_H^+^	Chloroform	0.01	0.04
120.066	C_4_H_9_NO_3_H^+^	Aminohydroxybutyric acid	<.0001	0.003
121.955	C_3_HCl_2_NH^+^	Dichloroacrylonitrile	0.0003	0.003
124.047	C_3_H_9_NO_2_SH^+^		0.001	0.006
124.12			0.005	0.02
129.017	C_5_H_4_O4H^+^	Hydroxyfuroic acid	<.0001	0.003
130.091	C_6_H_11_NO_2_H^+^	Nitrocyclohexane	0.002	0.01
132.109	C_5_H_13_N_3_OH^+^	Aminobutylurea	0.009	0.03
136.083	C_5_H_13_NSOH^+^	Methioninol	0.006	0.02
136.952	C_3_H_4_OSeH^+^	Oxopropaneselenal	0.002	0.008
137.131	C_10_H_16_H^+^	Monoterpenes	0.0005	0.004
137.959	C_3_H_5_OSeH^+^	Propeneselenenic acid	0.0002	0.003
138.062	C_4_H_11_NO_2_SH^+^	Tertbutylsulfonamide	0.003	0.01
142.123	C_8_H_15_NOH^+^	Cyclohexylacetamide	0.005	0.02
143.123	^13^CC_7_H_15_NOH^+^	^13^C-cyclohexylacetamide	0.002	0.008
144.065	C_6_H_9_NO_3_H^+^	2-Methoxyethyl cyanoacetate	0.0001	0.003
149.095	C_10_H_12_OH^+^	Estragole	0.01	0.04
153.055	CH_8_O_3_H^+^	Methyl hydroxybenzoate	0.0006	0.004
153.126	C_10_H_16_OH^+^	Monoterpene carbonyls (e.g. camphor)	0.0006	0.004
158.089	C_6_H_11_N_3_O_2_H^+^		0.0007	0.004
158.96			0.002	0.008
159.137	C_9_H_18_O_2_H^+^	Nonanoic Acid	0.01	0.04
161.132	C_12_H_16_H^+^	Benzene	0.01	0.04
162.908			0.002	0.01
165.092	C_10_H_12_O_2_H^+^	Phenethyl acetate	0.003	0.01
168.184			<.0001	0.003
169.122	C_10_H_16_O_2_H^+^	Geranic acid, Massoia lactone, Jasmine lactone	0.003	0.01
171.117	C_13_H_14_H^+^	Isopropylnaphthalene	0.002	0.008
177.009			0.004	0.02
178.015			0.002	0.01
178.06	C_6_H_13_N_2_S_2_H^+^		<.0001	0.003
182.156	C_11_H_19_ONH^+^	N-ethyl trans-2-cis-6-nonadienamide	0.0003	0.003
185.189	C_12_H_24_OH^+^	Methylundecanal	0.01	0.03
189.162	C_9_H_20_N_2_O_2_H^+^	Diaminononanoic acid	0.006	0.02
195.135	C_7_H_18_O_4_N_2_H^+^	1-[2-(2-Hydroxyethylamino)ethylamino]propane-1,2,3-trio	0.0004	0.003
200.206	C_12_H_25_NOH^+^	Dodecanamide	<.0001	0.003
201.114	C_10_H_16_O_4_H^+^	Camphoric Acid	0.005	0.02
204.186	C_8_H_21_N_3_OH^+^		0.0004	0.003
214.167	C_9_H_19_N_5_OH^+^		0.007	0.02
219.177	C_12_H_26_OSH^+^	Dihexyl sulfoxide, 12-Mercapto-1-dodecanol	0.003	0.01
222.159	C_12_H_19_N_3_OH^+^		0.0001	0.003
238.872			0.01	0.03
247.241	C_18_H_30_H^+^	Dodecylbenzene	<.0001	0.003
251.271	C_18_H_34_H^+^	1-Octadecyne	0.0005	0.004
262.262	C_19_H_33_H^+^/C_12_H_31_N_5_OH^+^		0.0002	0.003
267.008	C_11_H_10_N_2_S_3_H^+^/C_10_H_6_N_2_O_5_SH^+^	5-[(1Z,2E)-3-(5-Nitro-2-furanyl)-2-propenylidene]thiazolidine-2,4-dione	0.005	0.02
268.983	C_10_H_8_N_2_O_2_SeH^+^		0.008	0.03
283.266	C_18_H_34_O_2_H^+^	Oleic Acid, Elaidic Acid	<.0001	0.003
298.085			0.0003	0.003
300.065	C_14_H_10_ClN_5_OH^+^		0.007	0.02
301.289	C_22_H_36_H^+^		<.0001	0.003
317.079			<.0001	0.003
375.081			0.0006	0.004
390.117			<.0001	0.003
442.126			0.0002	0.003
444.123			0.002	0.01
445.107			0.0001	0.003
447.095			0.004	0.02
461.136			0.0007	0.004
464.125			0.0002	0.003

The concentration of CO_2_ from the carpet with dust samples incubated at 95% ERH was higher than the concentration from the carpet without dust incubated at the same ERH (Figure S17). The average CO_2_ concentration at 95% ERH for carpet with dust samples was 5.92±1.15 mg hr^-1^ kg^-1^ while the average for carpet without dust at 95% ERH was 2.55±1.22 mg hr^-1^ kg^-1^. CO_2_ data at other ERH conditions (65%, 70% and 75%) did not reveal differences in concentration of CO_2_ from carpet with dust and carpet without dust (Figure S18).

The collection location samples consisted of carpet without dust, carpet with dust, dust only samples, drywall only and inoculated drywall collected from CA, OH, and FL that were incubated at either 50% or 85% ERH consistently for two weeks. As these samples and were sampled on the PTR-TOF-MS for ~3 minutes using a “snap-shot” approach they cannot be used to report quantitative results. Concentrations from this data therefore provide a semiquantitative look at changes in compositional signatures. Four compounds that were associated with carpet with dust at 95% ERH from the moisture availability samples, C_2_H_6_SH^+^ (dimethyl sulfide + ethanethiol), CHCl_3_H^+^ (chloroform), C_10_H_16_H^+^ (monoterpenes) and C_10_H_16_O_4_H^+^ (camphoric acid,) were chosen to analyze in the collection location samples. No site differences for these compounds were found. The emissions profile of samples of carpet with dust from the different locations were visually similar (Figure S19).

### Microbial and chemical diversity

Increased equilibrium relative humidity was associated with decreased fungal species diversity, the number of different fungal species found within a given microbiome (Figure S20, linear regression *p*= 0.005, *R*^*2*^=0.82). For instance, only 92 fungal species were measured in the 95% ERH CA carpet and dust samples, while 501 fungal species were found in the 50% ERH CA carpet and dust samples. The data for carpet with dust samples were provided for the same 752 chemical VOC ions, however the abundance varied by six orders of magnitude and certain compounds were clearly associated with different ERH conditions. 38 ions were more associated with the High ERH condition when compared to both the Medium and Low ERH condition while 108 ions were more associated with Medium ERH compared to Low and High ERH and 103 ions were more associated with Low ERH compared to High and Medium ERH in the carpet with dust moisture samples.

## Discussion

Carpet with dust and drywall have different equilibrium relative humidity requirements to support fungal growth. Collection site contributed to microbial species composition, while type of substrate (carpet, dust, drywall) contributed to the release of VOCs and mVOCs. The release of VOC emissions was mainly due to the materials themselves; however certain compounds were determined as mVOCs at higher moisture conditions (>85% ERH).

### Excess moisture and microbial changes

Excess moisture altered both the amount of microbial growth and species type in the different building materials. In this study, microbial communities in carpet began to grow between 75% and 80% ERH and in drywall began to grow above 85% ERH. These findings are generally consistent with other studies [18, 20, 55] and highlight that the threshold relative humidity values to keep a home “dry” are localized. For instance, microbial communities on drywall in a bathroom are likely to be less responsive to episodic periods of high humidity than those in carpet in a living room. This emphasizes the need to include both dust and carpet in addition to drywall as they are both important reservoirs for human exposure [56]. Additionally, this reiterates concerns about the presence of carpet and similar materials in damp areas [57, 58].

Interestingly, carpet type and paint type on drywall did not strongly influence microbial species composition, while collection site did. There was no statistical difference when comparing the microbial communities in carpet A to those of carpet B across site location of dust origin. Both carpets were made of nylon carpet fibers, however carpet A was cut pile without any antimicrobial treatments and carpet B was loop pile with an antimicrobial treatment. Fiber type is known to influence the microbial composition in carpets [13] and may have influenced the similarity within microbial communities in these experiments. Collection site was associated with species composition when comparing samples of dust, carpet, and drywall from CA, OH, and FL. One factor that might influence this is geographic location [59]. The Chase (2016) study collected samples from different geographic locations over a period of one year, while our study utilized dust samples taken from multiple homes in different geographic locations at one time point. Previous work has shown that individual homes within a smaller geographic region of Ohio vary in taxonomic composition in response to excess moisture [19]. Because we sampled across multiple homes in a geographic location, we attribute variation across to be driven by geographic differences, but there may be additional variation due to home characteristics.

Despite this variation, the genera and species that we detected, such as *Penicillium*, *Aspergillus*, *Cladosporium*, and *Wallemia*, are common in the indoor environment [60–64]. The dependency of microbial communities on home of origin increases the challenge of identifying microbial indicators for dampness that are superior to visual inspection and detection of moldy odor as these indicators are qualitative at most [10].

### Understanding the emissions profile

Under elevated relative humidity conditions, the majority of the emissions from these samples were likely from the substrate: carpet, dust, and drywall, and not from growing microorganisms and their metabolic processes. Building materials have been recognized as a strong source of VOCs with sizeable emissions from carpets, textile materials, adhesives, wallboards, sealants, and urethane coatings [65]. Emissions of VOCs are highly dependent on substrate, with one study determining 70% of the total indoor VOCs attributed to different indoor sources such as household products, combustion processes, deodorizers and building materials [66]. C_5_H_10_H^+^ (cyclopentane/pentene), C_4_H_8_H^+^, (butanol/butene) and C_3_H_4_H^+^ (propyne) were abundantly emitted from samples of carpet without dust at 95% ERH, while CH_2_O_3_H^+^ (performic acid), C_2_H_5_NO_2_H^+^ (methyl carbamate), C_6_H_6_O_2_H^+^ (2-acetylfuran), C_6_H_6_H^+^ (benzene), and C_3_H_6_H^+^ (propene) were associated with autoclaved drywall at 95% ERH. These VOCs are all commonly associated with building materials [67]. Formaldehyde and acetaldehyde were also emitted from the carpet samples. One study found that carpet is the most important factor contributing to formaldehyde and acetaldehyde concentrations indoors [68]

We observed a lower emission factor from carpet with dust (500 – 2000 μg hr^-1^ g^-1^ carpet) than carpet without dust (800 – 3000 μg hr^-1^ g^-1^ carpet). Other studies have observed dust as a sink for semi-volatile organic compounds [69, 70]. We hypothesize that dust itself absorbs volatile emissions from the carpet, although further study is needed.

### VOCs with abiotic and biotic sources

Many compounds in this study were determined to have both biological and non-biological potential sources, a common obstacle when studying volatile chemicals in complex environments [71, 72]. The compound C_6_H_6_OH^+^, m/z 95.0468, was identified as phenol, and may be emitted from both a microbial and carpet source. Phenol is involved in the process of generating two intermediate products that are used in nylon production: caprolactam and bisphenol [73]. Phenol is also emitted by soil bacteria as a nematocidal volatile and by endophytic microorganisms in a symbiotic relationship with certain plant species [74, 75]. Other detected VOCs including acetic acid and acetaldehyde have both anthropogenic and microbial sources [37, 76–78]. It is difficult to distinguish, even at the high ERH where increased microbial growth was observed, the individual sources of the emissions from carpet, drywall, dust, or microbes. Delineating compounds in a complex system such as carpet is difficult without use of multivariate approaches which can determine the exact factor loadings for each compound. Another approach may be separately analyzing each component of a material to determine which compounds are emitted from each component. Future studies of isotopically labeled compounds could also elucidate sources.

Despite the difficulty in explicitly separating the building material emissions from the microbial emissions, utilizing the FDR analysis and the differences in observed chemical families at each ERH suggests that microbial emissions contribute to the overall VOC fingerprint. For homes without moisture damage, microbial emissions may not be an abundant source of emissions indoors relative to other sources. However, they are still an important part of the overall emissions profile, especially in regard to their reactivity, health concerns, and role as an odorant and irritant [11, 79, 80]. Limonene, a compound that has been identified as a common indoor mVOC, reacts quickly with ozone creating products that are active contributors to indoor chemistry [81, 82]. The products of the ozone-limonene reaction include several allergens, some of which pose a greater health concern than limonene itself [83]. Stable mVOCs like 1-butanol are also relevant to health concerns, as experiments where bronchoalveolar lavage cells were incubated with 1-butaonl lead to increased histamine production [84, 85]. mVOCs also play a substantial role in odor irritation indoors and the overall comfort of residents, as the odor threshold of VOCs is typically one to four orders of magnitude lower than that for irritation of the airways [79]. It is possible that longer incubation times at increased moisture conditions may have resulted in the microbial emissions dominating the emissions profile.

### Relationship between abiotic factors and VOCs

The relationship between VOCs and relative humidity is another factor that complicates interpretation of the overall emissions profile. The relationship between microbial growth and relative humidity is fairly well studied, with elevated relative humidity leading to more growth in microbial communities [18, 19]. One possibility is that as microbial growth increases so does the release of mVOCs in the indoor environment [61]. However, it is also possible that the mVOC profiles change, rather than increasing the total abundance increases, as the microbes transition through their life cycle [12, 86]. These ideas are not mutually exclusive. That is, certain mVOCS may be released at elevated relative humidity during primary metabolism, while others may be released when growth has ceased. The mVOC abundance is expected to change proportionally with the changes in microbial density that may span orders of magnitude [35].

Increasing relative humidity can either increase or decrease the emissions of VOCs and the sink effect of the material [87]. The impact of relative humidity level is dependent on both the material and the specific VOC, as multiple phenomena such as diffusion, adsorption, and condensation may alter results [88] and lead to non-linear emissions in response to moisture [87]. Indeed, in our study, the relative humidity level at which a compound had the highest abundance was dependent on processes such as the specific compound and type of sample (e.g. carpet without dust, carpet with dust) that simultaneously affected the emissions profile. For example, polar compounds such as acetate and acetic acid had higher abundance of emissions at lower ERHs (50%, 65%) then compared to 95% ERH. Wolkoff (1998) observed similar results and attributed it to desorption of specific compounds to the water vapor at high relative humidity. However, this result does not extend to all polar compounds as seen by Markowicz and Larsson (2015), which further emphasizes the compound specificity in regard to moisture.

New technology allows us to address an expanding set of questions pertaining to mVOCs [29, 89–91]. mVOC emissions are not constant and can be affected by substrate type, temperature, pH, moisture content, and potentially other factors, many of which are abiotic factors that change frequently in an indoor environment [92, 93]. This reality highlights the complexity in attempting to determine a single mVOC as an indicator of fungal growth indoors. We suggest that future work should not focus solely on separating microbial emissions from anthropogenic emissions, as mVOCs are inherently connected to their environment which is dominated by anthropogenic factors. Rather, the focus should be on characterizing the attributes of an overall emissions profile with a microbial contribution.

### Limitations

This research was conducted in a laboratory setting utilizing glass incubation chambers that do not fully represent the indoor environment. The indoor environment consists of varying factors that may disrupt changes in temperature and humidity that may not be represented through our chamber experiments. The major differences are the static incubation timescales where RH was not cycled and was conducted only for a relatively short period (two to four weeks) compared to real indoor environments. However, findings from this study emphasize the importance of maintaining proper relative humidity in homes to limit microbial growth, VOC, and mVOC emissions.

Multiple compounds with different structures may be associated with some chemical formulas. The compound specified in parentheses corresponds to the isomer that was most consistent with a formula based on relevant properties of the compound such as vapor pressure, boiling point, and knowledge of chemical composition of the source. However, in case of larger molecules or multiisomeric formulas, we cannot conclusively say that a certain chemical isomer was emitted from these materials, in which case we refer just to the chemical formula. Due to a technical issue with the PTR-TOF-MS (drift module failure) which occurred on the day of 85% ERH sample collection, emissions data collected from these samples were not used in the analysis.

All samples of carpet and drywall were initially autoclaved and baked overnight at 30°C to create “sterile non-microbial” conditions to be used as control samples. Autoclaving the carpet may have impacted thermally unstable compounds accelerating degradation and other transformations within the materials. Vacuumed particles on filters and swabs collected from samples of only carpet without dust and drywall (no inoculation) did amplify on qPCR, with carpet without dust quantities ranging from 10^2^ – 10^3^ spore equivalents/mg filter dust and autoclaved drywall ranging from 10^3^ – 10^5^ spore equivalents/cm^2^ drywall. When sequenced on the Illumina MiSeq, however, these samples were removed during quality filtering and no species were determined. Dust irradiated with a 10 kGy electron beam was plated on growth media and some microbial colonies were observed, though much less growth occurred when compared to that of the non-ionized dust. Therefore, these samples may have also had a minor contribution from residual microbial communities.

## Conclusions

Moisture plays an important role in regulating microbial growth and VOC/mVOCs emissions. This study demonstrates the importance of comparing indoor materials such as carpet and drywall as each material represents a distinct component of indoor chemical and microbial exposures. Growth in dust is also distinctly different than that of growth on drywall as we determined that fungal growth in dust requires a lower level of moisture than that of drywall. Understanding resuspension rates of dust in homes and clear knowledge of the chemical compounds from dust at humidity conditions above 75% are needed. Proper humidity conditions must also be maintained as different emissions may be released under different moisture conditions. We found that moisture plays a key role in the release of both VOC and mVOCs, though the relationship is nonlinear.

These results have broad implications for managing growth and emissions in the indoor environment to ensure proper human health. Current detection of mold growth indoors is constrained to visual inspection or moldy odor detection as we lack a quantitative means of mold measurement. Utilizing mVOC emissions in mold detection may have important implications for the future of mold level detection in housing. Continued work is necessary to determine mVOCs from common fungal species indoors. However, this may be difficult as fungal species in homes vary by home environment and collection site. Ultimately these results highlight the necessity for continued work and complex relationships between moisture, microbes, and chemicals in the indoor environment.

## Supplementary Information

**Additional file 1.**

**Additional file 2.**
