# Correction to: Microbial growth and volatile organic compound (VOC) emissions from carpet and drywall under elevated relative humidity conditions

**DOI:** 10.1186/s40168-021-01179-7

**Published:** 2021-11-07

**Authors:** Sarah R. Haines, Emma C. Hall, Katarzyna Marciniak, Pawel K. Misztal, Allen H. Goldstein, Rachel I. Adams, Karen C. Dannemiller

**Affiliations:** 1 Department of Civil & Mineral Engineering, University of Toronto, Toronto, Ontario M5S 1A4 Canada; 2 Department of Civil, Architectural and Environmental Engineering, University of Texas at Austin, Austin, TX 78712 USA; 3 School of Chemistry, The University of Edinburgh, Edinburgh, EH9 3FJ UK; 4 Department of Environmental Science, Policy and Management, University of California, Berkeley, CA 94720 USA; 5 Department of Plant and Microbial Biology, University of California, Berkeley, CA 94720 USA; 6 Department of Civil, Environmental & Geodetic Engineering, College of Engineering, Ohio State University, Columbus, OH 43210 USA; 7 Division of Environmental Health Sciences, College of Public Health, Ohio State University, Columbus, OH 43210 USA; 8 Sustainability Institute, Ohio State University, Columbus, OH 43210 USA; 9 Department of Civil, Environmental & Geodetic Engineering, Environmental Health Sciences, Ohio State University, 470 Hitchcock Hall, 2070 Neil Ave, Columbus, OH 43210 USA

**Correction to: Microbiome 9, 209 (2021)**

**https://doi.org/10.1186/s40168-021-01158-y**

Following the publication of the original article [1], the author reported that there is an error in Table 2. Three of the drywall sample are under the dust header.

The original article has been updated.
