# Supplementary material for: Microbial growth and volatile organic compound (VOC) emissions from carpet and drywall under elevated relative humidity conditions
Source: Microbiome. 2021 Oct 19;9:209. doi: 10.1186/s40168-021-01158-y (PMC8524935; doi:10.1186/s40168-021-01158-y)
Supplement: Supplementary file 2 — Additional file 1. [file 40168_2021_1158_MOESM2_ESM.docx]

Online supporting information for:

**Microbial growth and volatile organic compound (VOC) emissions from carpet and drywall under elevated relative humidity conditions**

Sarah R. Haines^1^, Emma C. Hall^2^, Katarzyna Marciniak^3^, Pawel K. Misztal^2^, Allen H. Goldstein^4^, Rachel I. Adams^5^, Karen C. Dannemiller^6,7,8^*

1. *Department of Civil & Mineral Engineering, University of Toronto, Toronto, Ontario M5S 1A4, Canada*
2. *Department of Civil, Architectural and Environmental Engineering, University of Texas at Austin, Austin, TX 78712*
3. *School of Chemistry, The University of Edinburgh, Edinburgh EH9 3FJ, UK*
4. *Department of Environmental Science, Policy and Management, University of California, Berkeley, CA 94720*
5. *Department of Plant and Microbial Biology, University of California, Berkeley, CA 94720*
6. *Department of Civil, Environmental & Geodetic Engineering, College of Engineering, Ohio State University, Columbus, OH 43210*
7. *Division of Environmental Health Sciences, College of Public Health, Ohio State University, Columbus, OH 43210*
8. *Sustainability Institute, Ohio State University, Columbus, OH 43210*

*Corresponding author: Karen C. Dannemiller, Department of Civil, Environmental & Geodetic Engineering, Environmental Health Sciences, Ohio State University, 470 Hitchcock Hall, 2070 Neil Ave, Columbus, OH 43210, [Dannemiller.70@osu.edu](mailto:Dannemiller.70@osu.edu), 614-292-4031

**Contents**

**Supplemental Methods**……………………………………………4 – 10

**Supplemental Results**…………………………………………….10

**Figures**

Figure S1……………………………………………………………………11

Figure S2……………………………………………………………………12 – 20

Figure S3……………………………………………………………………21

Figure S4……………………………………………………………………22 – 25

Figure S5……………………………………………………………………26

Figure S6……………………………………………………………………27 – 29

Figure S7……………………………………………………………………30

Figure S8……………………………………………………………………31

Figure S9……………………………………………………………………32

Figure S10..…………………………………………………………………33 – 34

Figure S11..…………………………………………………………………35 – 36

Figure S12..…………………………………………………………………37 – 38

Figure S13..…………………………………………………………………39

Figure S14..…………………………………………………………………40

Figure S15..…………………………………………………………………41

Figure S16..…………………………………………………………………42

Figure S17..…………………………………………………………………43

Figure S18..…………………………………………………………………44

Figure S19..…………………………………………………………………45

Figure S20..…………………………………………………………………46

**Table Headings**

*All tables are located in the “Online_Supporting_Information_Tables.xlsm”. The table descriptions can be found in this document.

Table S1..…………………………………………………………………47

Table S2..…………………………………………………………………47

Table S3..…………………………………………………………………47

Table S4..…………………………………………………………………47

Table S5..…………………………………………………………………47

Table S6..…………………………………………………………………47

Table S7..…………………………………………………………………47

Table S8..…………………………………………………………………47

Table S9..…………………………………………………………………47

Table S10..………………………………………………………………..48

Table S11..………………………………………………………………..48

Table S12..………………………………………………………………..48

Table S13..………………………………………………………………..48

Table S14..………………………………………………………………..48

Table S15..………………………………………………………………..48

Table S16..………………………………………………………………..48

Table S17..………………………………………………………………..48

Table S18..………………………………………………………………..48

Table S19..………………………………………………………………..48

**References**.………………………………………………………………49

**Supplemental Methods**

*Drywall sample inoculation*

In San Francisco, CA, home 1, 38 squares of Drywall A and 6 squares of Drywall B were left in a bedroom at a height of 71 cm, while in home 2, 11 squares of Drywall A and 6 squares of Drywall B were left in a front living room. In Columbus, Ohio 6 squares of Drywall A and 6 squares of Drywall B were left in a bedroom at a height of 183 cm at Ohio home 1 and in a living room at a height of 94 cm in Ohio home 2

*qPCR and DNA sequencing*

For fungal qPCR, forward primer, FF2, 5′-GGTTCTATTTTGTTGGT TTCTA-3′ and reverse primer, FR1, 5′-CTCTCAATCTGTCAATCCTTATT-3′ were used along with SYBR® Green[1]. For bacteria, forward primer 5'-TCCTACGGGAGGCAGCAGT-3', reverse primer 5' GGACTACCAGGGTATCT AATCCTGTT-3' and probe (6-FAM)-5'-CGTATTACCGCGGCTGCTGGCAC-3'-(BHQ) were used [1–3]. targeting the 16S rRNA gene along with TaqMan® master mix. Standards were created using *Bacillus atrophaeus* (ATCC® 49337™) for bacterial standards, and Aspergillus fumigatus (ATCC® MYA-4609™) for fungal standards. Methodology on creation of standards can be located in Haines et al 2020 [4]. The qPCR parameters used an initial hold stage set at 50°C for 2 minutes and then 95°C for 15 minutes with a PCR stage of 40 cycles of denaturation at 95°C for 15 seconds and annealing at 60°C for 1 minute. Results of qPCR were expressed as spore equivalents/mg filter dust. The term “spore equivalents” is utilized to indicate that DNA may originate from different types of fungal fragments and that gene copies may vary between different species. Calculation of the final fungal concentration accounted for the final DNA extraction elution volume (50 µL), the dilution factor of 10x, and the weight of the dust and filter used in the DNA extraction.

About 25 µL of each DNA extracted sample was sent to RTL Genomics in Lubbock, TX. DNA was sequenced on an Illumina MiSeq using bacterial 16S 515F (5'-GTGCCAGCMGCCGCGGTAA) and 806R (5'-GGACTACHVHHHTWTCTAAT) sequencing primers [5] and fungal ITS1F (CTTGGTCATTTAGAGGAAGTAA) and ITS2aR (GCTGCGTTCTT CATCGATGC) ribosomal DNA primers with 2x300 bp chemistry [6].

To analyze the sequencing data, the bioinformatics pipeline QIIME version 1.9 was utilized [7]. We received the sequencing data as raw .fastq files and trimmed the primers and spacers, joining the paired ends using the SeqPrep method. To determine taxonomy of the sequences, BLAST version 2.2.28 [8], the UNITE database 2019 [9] and FHiTINGS version 1.5 [10] were utilized. For determination of bacterial species identification, we used the GreenGenes Database version 13_5 [11]. The absolute abundance of each species in each sample was calculated by first determining the relative abundance of each species and multiplying the relative abundance by the quantity of spores or cells determined through qPCR.

Blank samples of both carpet and drywall were also processed alongside the incubations such that these samples should not have had any microbial growth. However, results from qPCR and DNA sequencing did reveal samples not fully devoid of microbes. The quantity of bacterial cells on samples of autoclaved painted drywall, ranged from 0 to 1.44 x 10^5^ cells/cm^2^ drywall. No microbial species were identified in these samples from sequencing. Samples of San Francisco, CA drywall B were removed from the analysis due to poor quality.

*Statistical analysis*

The beta diversity was determined utilizing the Bray-Curtis dissimilarity statistic in QIIME and a principal coordinate analysis (PCoA) was created. For bacteria, weighted and unweighted UniFrac analysis was utilized at it account for the phylogenetic relatedness of the operational taxonomic units (OTUs) and the abundance while the Bray-Curtis dissimilarity statistic only considers abundance. Again, PCoA plots were created to display differences within the bacterial taxonomy.

Using the FDR adjustment procedure comparisons were made such as location of sampling and type of sample (dust, carpet with dust, drywall). Similar tests were used to determine statistically significant chemical emission from the moisture availability samples, comparing dust embedded in carpet samples with just carpet without dust at low (50%-70%), medium (75%-85%) and high (95%) ERH conditions as well as inoculated drywall and autoclaved drywall at varying humidity levels.

Comparisons were also made across humidity levels within the dust embedded in carpet samples and inoculated drywall samples alone. For the collection site samples that included dust and drywall inoculated in Ohio, California and Florida, comparisons were made between location, material type and relative humidity level, either 50% or 85% ERH.

*VOC Measurement with PTR-TOF-MS*

The PTR-TOF-MS uses chemical ionization with a proton transfer reaction to detect, in real time, VOCs across a broad mass spectrum with high sensitivity [12]. The raw data were collected throughout the measurement campaign in the multidimensional hierarchical data format (HDF5) which were processed into the units of counts per second (cps) by the PTRwid set of routines on an IDL platform. The PTRwid routine also was used for pre-processing preliminary concentration data in parts per billion using the theoretical detector transmission and VOC default proton transfer reaction rate constant [13]. Both the raw cps data and the concentration data were separated using MATLAB routines, into the different, individual experiments preformed throughout the campaign, leading to 46 independent data sets. The valve data from the multi-port valve system was then used to further separate these 46 data sets down to the individual jars, corresponding to one singular sample. Each 7.5-minute data set was trimmed of the first 3-minutes and the final minute to prevent any overlap from signal to signal.

Valve data was also used to identify when calibration gas and Zero Air were flowing through the system and these two sets of data were separated from the sample concentration data. Zero Air concentration data was interpolated across the entire sample concentration data set, which then allowed for an overall background subtraction of the Zero Air.

*Calibration, Sensitivity, and Concentration Calculations*

The separated calibration data were then used to calculate the normal sensitivity, which is the slope between the normalized counts per second rates and the concentration data [14]. Sensitivity values are dependent and specific to the compound being measured and are used to calculate the limit of detection, which provides the theoretical minimum concentration of that ion that the instrument could detect at given time resolution [14]. The calibration gas standard was always diluted with Zero Air, and therefore the overall uncertainty can be considered to be 10% with uncertainty associated with the standard itself and the dilution of Zero Air [14]. Over 60 calibrations were successfully performed over the course of the campaign, so an average sensitivity value was identified for each compound in the calibration gas standard except for those select compounds noted in the paper.

For compounds present in the calibration standards, the average sensitivity values were then used to recalculate the concentration data for the compounds in the calibration standard using their corresponding counts-per-second data. For compounds not in the calibration gas standard, the concentration data was recalculated using the proton transfer reaction theory and reaction rate coefficients [14,15]. Known reaction rates were normalized by the standard reaction rate coefficient of k=2.5, and the correction factor was applied to the concentration data. While a large number of identified compounds had known reaction rate coefficients a multitude of compounds were neither in the calibration standard nor had known reaction rate coefficients . The concentration data for these compounds was calculated using the default reaction rate coefficient, which for most VOCs is up to 30% of uncertainty but can be larger for low-volatility compounds due to potential losses in the analytical system. Our analysis focused on relatively high volatility of VOCs which embrace part of the most volatile spectrum of semi-volatile organic compounds (SVOC) which in general had boiling points below 350 C. The less volatile compounds which may still have been detected are assumed a non-significant fraction of the overall sum of VOCs and therefore the larger uncertainty to this group of compounds does not affect the overall accuracy of our dataset. Due to the canceling effect of variability in proton transfer reaction rate constants the overall uncertainty for the sum of VOCs is likely below 30% [16,17].

*Formula, Compound, and Source Identification*

Overall, the post-processing routines that were performed in MATLAB included data separation, Zero Air background subtraction, calibration and sensitivity calculations, removal of data unfulfilling the quality control criteria, trimming of data to eliminate any residual from the switching of samples, averaging of data, applied abundance thresholds, and identifying the chemical formulas and most probable compound identities.

The high mass resolving power of the PTR-TOF-MS allows for the separation of isobaric compounds on their nominal mass [18]. With this high level of separation, the molecular formula of most compounds can be determined with high confidence. A variety of techniques were used to compile the final list of chemical formulas using a combination of automated and manual approaches. First, PTRwid pre-processing produces a list of possible chemical formulas and the fractional mass deviation from the mass corresponding to the mass-to-charge ratio [15]. PTRwid only considers C, ^13^C, H, N, and O when compiling this list [15]. A MATLAB routine is then used to determine the “Best Guess Formula” out of the possibilities identified by PTRwid based upon the mass deviation values. All compounds that had an m/z ratio <200 and any compounds that were used in analysis had their corresponding formula manually verified through a variety of resources including ChemSpider, ChemCalc, and relevant literature. All identified isotopies were also verified through correlation techniques and the comparison of the concentration data for the parent ion and isotope. In some cases, including when no formula was assigned, the “Best Guess Formula” was not feasible based on the boiling point, vapor pressure, or number of oxygens; or when a possible formula at an m/z ratio included elements other than those used in PTRwid (S, Cl, F, Se or Si) the intrinsic properties (deprotonated monoisotopic mass within 2 mDa) on ChemSpider were used to assign the formula. For some m/z ratios no formula was assigned either due to too many possible formulas, or in some rare cases because no formulas corresponded to that m/z ratio .

For one chemical formula numerous structures, and therefore compounds, are possible, and a tentative compound identification was assigned to most compounds. The same resources that assisted in the verification of the chemical formulas also helped to identify the tentative compound, and additionally the bioinformatic mVOC2.0 database was used extensively to determine if a compound had a possible microbial nature [19]. The most probable source of the VOC was distinguished between microbial, building material, or both for some compounds. Most compounds were seen to have both possible microbial and building material sources, but due to the extensive number of m/z ratios seen and the ever-evolving literature on mVOCs misidentification of sources is possible.

*Emission Rate Calculation*

Emission rates (µg/hr) were calculated for the 2-hour moisture availability samples 1 using the equation outlined below (Equation S1).

$E\left( t \right)=Concentration\left( ppb \right)*Molecular Weight \left( \frac{g}{mol} \right)*200\frac{cm^{3}}{min}*\frac{mol}{24.5 L}*60\frac{min}{hr}*\frac{1}{{10}^{6}}$(S1)

The concentration value used in the equation is the concentration of the sample (Jar 2-7) subtracted by the concentration of the empty jar that served as a negative control (Jar 1). The emissions rates were calculated using concentration data that was first trimmed to the final 3.5 minutes of each sampling cycle, as this was determined to be the amount of time necessary for equilibration. The value of $200\frac{cm^{3}}{min}$ is the flowrate that was flushed continuously through each jar and $24.5\frac{L}{mol}$ represents the volume of one mole of an ideal gas at 25 C and 101,325 Pa.

**Supplemental Results**

A total of 3,732,213 fungal DNA sequences and 5,122,449 bacterial DNA sequences were included in the analysis. Different fungal species were also associated with each collection location in both carpet with dust samples and drywall samples. In all samples (moisture availability and geographic samples) *Wallemia, Aspergillus* and *Penicillium* were the most abundant fungal genera while Streptococcus, Staphylococcus and Corynebacterium were the most abundant bacterial genera. Over 700 reported mass-to-charge ratios from the PTR-TOF-MS were measured in these experiments. Chemical emissions were mainly associated with the building materials themselves, though certain compounds were determined as likely sourced from microorganisms. C_5_H_10_H^+^ (consistent with cyclopentane/pentene), C_4_H_8_H^+^, (consistent with butanol/butene) and C_3_H_4_H^+^ (consistent with propyne) were abundantly emitted from samples of carpet with dust and carpet without dust.

**Figures**


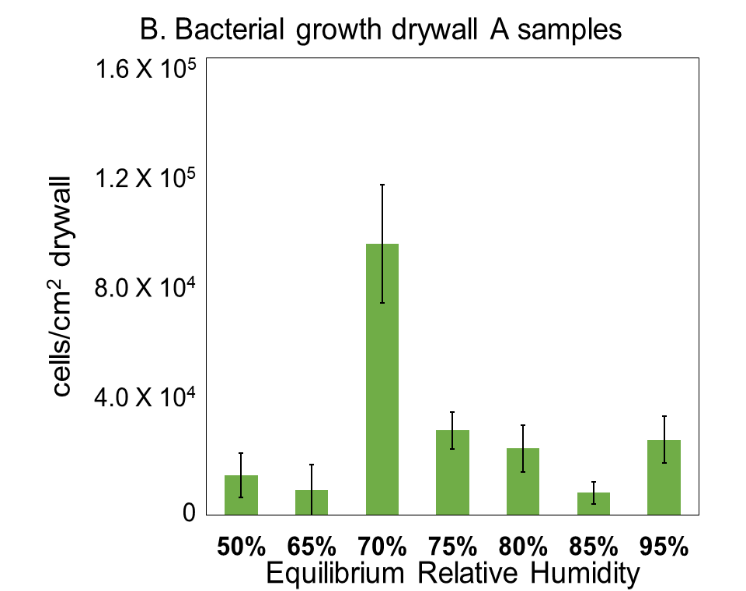

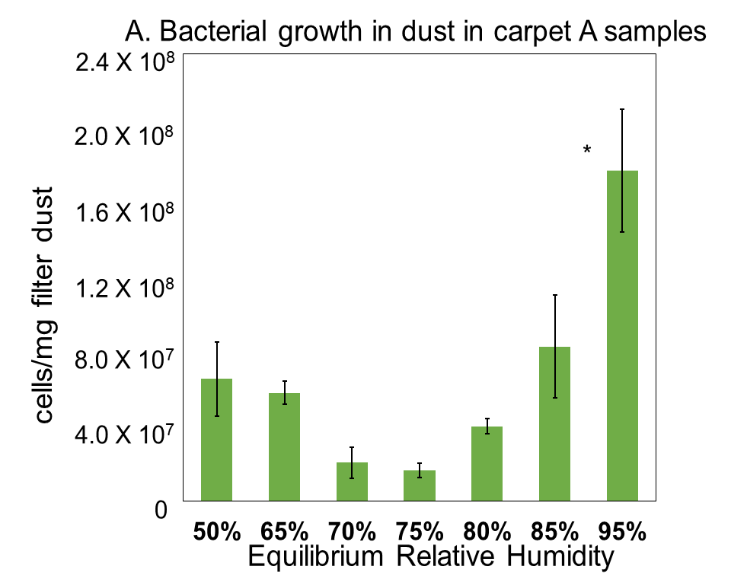


**Figure S1**. Measured values of bacterial cells/mg filter dust and cells/cm2 drywall determined from qPCR of the CA dust embedded in carpet A samples (A) and CA inoculated drywall samples (B) respectively at each relative humidity condition. These samples were part of the moisture availability samples that were incubated at 50%, 65%, 70%, 75%, 80%, 85% or 95% for 4 weeks continuously. *New samples of dust and carpet were incubated at 95% ERH for 4 weeks continuously at a later date to determine accurate quantities of fungal growth. Original samples were inconclusive at 95% as the carpet was too wet to collect

|  | **Low** | | | **Medium** | | | **High** |
| --- | --- | --- | --- | --- | --- | --- | --- |
| **Fungal Species** | **50%** | **65%** | **70%** | **75%** | **80%** | **85%** | **95%** |
| *Penicillium chrysogenum* | 2032 | #### | 7481 | 807 | 0 | #### | #### |
| *Aspergillus hongkongensis* | 1217 | 9041 | 1842 | 352 | 1005 | 4239 | #### |
| *Penicillium citrinum* | 24.42 | 250.4 | 49.94 | 0 | 594.5 | 1098 | 5E+05 |
| *Penicillium gladioli* | 305.7 | 1060 | 859.4 | 116.5 | 0 | 394 | 3E+05 |
| *Wallemia tropicalis* | #### | #### | #### | #### | #### | #### | #### |
| *Aspergillus sydowii* | 253 | 873.9 | 399.1 | 0 | 0 | 414 | 2E+05 |
| *Penicillium kongii* | 968 | 2343 | 707 | 276 | 440 | 2420 | #### |
| *Wallemia muriae* | #### | #### | #### | #### | #### | #### | #### |
| *Wallemia canadensis* | #### | #### | #### | #### | #### | #### | #### |
| *Aspergillus ruber* | #### | #### | #### | #### | #### | #### | #### |
| *Wallemia sebi* | 8149 | #### | #### | #### | #### | #### | #### |
| *Penicillium concentricum* | 0 | 41.05 | 0 | 0 | 0 | 187 | 11818 |
| *Cladosporium halotolerans* | 1258 | 2385 | 970.4 | 164.5 | 0 | 2066 | 11801 |
| *Aspergillus nidulans* | 4.223 | 165.6 | 13.7 | 0 | 0 | 207 | 11382 |
| *Penicillium expansum* | 164.6 | 386.8 | 200.7 | 116.5 | 0 | 601 | 7685 |
| *Alternaria terricola* | 2069 | 862.7 | 344.8 | 219.4 | 0 | 0 | 7664 |
| *Penicillium sclerotigenum* | 0 | 109.9 | 162.1 | 61.68 | 0 | 0 | 7628 |
| *Penicillium decumbens* | 284.9 | 1121 | 843.3 | 303.9 | 0 | 0 | 7455 |
| *Penicillium antarcticum* | 0 | 55.65 | 24.97 | 0 | 0 | 0 | 4160 |
| *Candida parapsilosis* | #### | #### | 5003 | #### | #### | #### | 4079 |
| *Penicillium herquei* | 0 | 441.1 | 214.4 | 61.68 | 0 | 601 | 3678 |
| *Cladosporium sphaerospermum* | 726.4 | 1042 | 1149 | 54.85 | 0 | 1202 | 3654 |
| *Penicillium brevicompactum* | 0 | 139.1 | 27.4 | 0 | 0 | 0 | 2924 |
| *Aspergillus austroafricanus* | 0 | 250.5 | 0 | 0 | 0 | 0 | 2790 |
| *Botryotrichum atrogriseum* | 45.54 | 0 | 13.7 | 0 | 0 | 0 | 2627 |
| *Schizothecium miniglutinans* | 253.6 | 262.3 | 156.8 | 0 | 0 | 0 | 2073 |
| *Cladosporium dominicanum* | 8431 | 7272 | 4453 | 370 | 595 | 2384 | 1848 |
| *Humicola nigrescens* | 672.5 | 55.65 | 369.3 | 123.4 | 0 | 0 | 1578 |
| *Aspergillus versicolor* | 0 | 181.6 | 68.51 | 0 | 0 | 207 | 1353 |
| *Disculoides calophyllae* | 257.2 | 83.48 | 0 | 0 | 0 | 0 | 953.2 |
| *Aspergillus niger* | 80.81 | 55.65 | 137.4 | 89.1 | 2648 | 2237 | 727 |
| *Aspergillus conicus* | 642 | 892 | 697 | 475 | 2522 | 9619 | 717 |
| *Exophiala dermatitidis* | 3663 | 480.1 | 510 | 0 | 0 | 414 | 717.4 |
| *Naganishia diffluens* | 900.4 | 678.3 | 335.8 | 246.7 | 0 | 0 | 691 |
| *Candida hyderabadensis* | 1339 | 224 | 192.7 | 0 | 0 | 0 | 691 |
| *Phaeotheca triangularis* | 36.79 | 68.88 | 0 | 175.9 | 0 | 561 | 691 |
| *Epicoccum nigrum* | #### | #### | #### | 2582 | 6660 | 9898 | 425 |
| *Talaromyces minioluteus* | 301.8 | 782.1 | 192.1 | 89.1 | 594.5 | 1115 | 424.8 |
| *Penicillium corylophilum* | 158.3 | 466.2 | 349.6 | 185 | 0 | 414 | 422.3 |
| *Thielavia terrestris* | 20.5 | 27.83 | 0 | 0 | 0 | 0 | 412.7 |
| *Alternaria nepalensis* | 151.7 | 219.9 | 126.6 | 0 | 0 | 0 | 374.3 |
| *Aspergillus restrictus* | 80.2 | 748.6 | 298.2 | 27.42 | 4108 | 4964 | 359.9 |
| *Figure S2 Continued* |  | | |  | | |  |
|  | **Low** | | | **Medium** | | | **High** |
| **Fungal Species** | **50%** | **65%** | **70%** | **75%** | **80%** | **85%** | **95%** |
| *Knufia marmoricola* | 1625 | 544.8 | 214.8 | 61.68 | 0 | 187 | 345.5 |
| *Naganishia antarctica* | 1046 | 828.7 | 187 | 0 | 0 | 0 | 132.1 |
| *Cryptococcus uniguttulatus* | 199 | 55.65 | 87.92 | 0 | 0 | 0 | 132.1 |
| *Penicillium polonicum* | 40.1 | 0 | 168.1 | 0 | 0 | 0 | 86.46 |
| *Cladosporium delicatulum* | #### | #### | #### | 1663 | 4151 | 3678 | 69.6 |
| *Nigrospora oryzae* | #### | #### | #### | 1567 | 595 | 1202 | 67.2 |
| *Debaryomyces hansenii* | 56.69 | 0 | 11.27 | 0 | 0 | 350.4 | 52.84 |
| *Mycosphaerella tassiana* | #### | #### | #### | 1071 | 4306 | 5821 | 26.4 |
| *Neoascochyta desmazieri* | 2493 | 288.7 | 247.2 | 0 | 0 | 414 | 26.42 |
| *Claviceps grohii* | 1236 | 580.2 | 1171 | 0 | 0 | 0 | 26.42 |
| *Kondoa changbaiensis* | 572 | 208 | 99.18 | 0 | 0 | 0 | 26.42 |
| *Stilbella byssiseda* | 131.8 | 0 | 143.1 | 0 | 0 | 207 | 26.42 |
| *Hamigera insecticola* | 56.38 | 292.9 | 203.9 | 116.5 | 2421 | 2504 | 21.61 |
| *Alternaria didymospora* | 53.68 | 199 | 43.96 | 0 | 0 | 0 | 21.61 |
| *Aureobasidium namibiae* | 5694 | 2838 | 2293 | 338.1 | 0 | 414 | 14.41 |
| *Naganishia albida* | 2875 | 2448 | 1126 | 214.8 | 1189 | 0 | 14.41 |
| *Phaeosphaeria podocarpi* | 1193 | 743.1 | 753 | 82.27 | 0 | 414 | 14.41 |
| *Preussia flanaganii* | 3726 | 344.4 | 291.2 | 212.5 | 0 | 0 | 14.41 |
| *Clavispora lusitaniae* | 40.1 | 0 | 27.4 | 114.2 | 410.7 | 0 | 14.41 |
| *Penicillium bilaiae* | 0 | 27.83 | 0 | 61.68 | 0 | 537.4 | 14.41 |
| *Pseudopithomyces chartarum* | #### | 6978 | 3339 | 713 | 1034 | 1178 | 7.2 |
| *Coniosporium apollinis* | #### | #### | 4357 | 635 | 1189 | 4276 | 7.2 |
| *Vishniacozyma victoriae* | 25881 | 16046 | 6252 | 833.9 | 1643 | 1115 | 7.205 |
| *Xenodidymella humicola* | 2931 | 2293 | 1227 | 598.6 | 0 | 1262 | 7.205 |
| *Candida tropicalis* | 648 | 514.2 | 161.1 | 52.54 | 0 | 0 | 7.205 |
| *Verrucaria elaeomelaena* | 622.4 | 345.8 | 157.5 | 0 | 0 | 207 | 7.205 |
| *Fumiglobus pieridicola* | 166.2 | 682.6 | 0 | 0 | 439.9 | 0 | 7.205 |
| *Schizothecium curvuloides* | 474.4 | 0 | 236.7 | 0 | 0 | 0 | 7.205 |
| *Periconia byssoides* | 54.29 | 27.83 | 219.8 | 0 | 0 | 0 | 7.205 |
| *Aspergillus penicillioides* | 1573 | 5619 | 2854 | 2666 | #### | #### | 0 |
| *Didymella gardeniae* | 6652 | 5336 | 16717 | 297 | 5481 | 2110 | 0 |
| *Vishniacozyma carnescens* | 8956 | 4616 | 3046 | 290.1 | 1189 | 1489 | 0 |
| *Aspergillus appendiculatus* | 200.5 | 1267 | 2454 | 1531 | 410.7 | 4885 | 0 |
| *Cladosporium ramotenellum* | 13649 | 8637 | 6040 | 274.1 | 0 | 2523 | 0 |
| *Aspergillus xerophilus* | 360.9 | 5930 | 824.7 | 413.5 | 1261 | 2424 | 0 |
| *Gibberella baccata* | 2271 | 1757 | 654.8 | 379.2 | 0 | 414 | 0 |
| *Microdochium phragmitis* | 2647 | 4045 | 1178 | 109.7 | 4577 | 700.8 | 0 |
| *Pyrenochaetopsis pratorum* | 1130 | 1229 | 396.7 | 27.42 | 594.5 | 1115 | 0 |
| *Lapidomyces hispanicus* | 2862 | 2922 | 1489 | 228.4 | 0 | 414 | 0 |
| *Papiliotrema laurentii* | 530.1 | 905.2 | 321.1 | 123.4 | 0 | 788 | 0 |
| *Alternaria rosae* | 2240 | 1471 | 780.4 | 171.4 | 0 | 350.4 | 0 |
| *Figure S2 Continued* |  |  |  |  |  |  |  |
|  | **Low** | | |  | **Medium** |  | **High** |
| **Fungal Species** | **50%** | **65%** | **70%** | **75%** | **80%** | **85%** | **95%** |
| *Neonectria major* | 405 | 711.8 | 352.7 | 116.5 | 0 | 350.4 | 0 |
| *Phoma saxea* | 6807 | 2622 | 1479 | 192 | 0 | 1162 | 0 |
| *Phaeococcomyces eucalypti* | 1359 | 1437 | 212.8 | 214.8 | 1189 | 0 | 0 |
| *Vishniacozyma heimaeyensis* | 3064 | 2021 | 858.9 | 159.9 | 1189 | 0 | 0 |
| *Phaeococcomyces mexicanus* | 2531 | 1465 | 1073 | 54.85 | 594.5 | 414 | 0 |
| *Alternaria alternata* | 9767 | 5897 | 4298 | 27.42 | 821.3 | 621.1 | 0 |
| *Protomyces inouyei* | 515.1 | 247.7 | 391.4 | 178.2 | 594.5 | 0 | 0 |
| *Epicoccum plurivorum* | 1459 | 558 | 503.6 | 178.2 | 0 | 0 | 0 |
| *Articulospora proliferata* | 1534 | 1827 | 740.5 | 123.4 | 0 | 207 | 0 |
| *Knufia perforans* | 344.1 | 464.1 | 151.2 | 123.4 | 0 | 187 | 0 |
| *Neodevriesia stirlingiae* | 1011 | 569.8 | 506.4 | 116.5 | 0 | 0 | 0 |
| *Buckleyzyma aurantiaca* | 1299 | 578.9 | 302.4 | 61.68 | 879.7 | 350.4 | 0 |
| *Saitozyma podzolica* | 185.1 | 896.9 | 203.9 | 61.68 | 439.9 | 0 | 0 |
| *Fusarium acutatum* | 518.4 | 1068 | 74.9 | 109.7 | 1189 | 3177 | 0 |
| *Cladosporium aphidis* | 4917 | 1799 | 1116 | 54.85 | 821.3 | 0 | 0 |
| *Knufia mediterranea* | 652.9 | 402.9 | 101.6 | 54.85 | 0 | 557.4 | 0 |
| *Plectosphaerella oratosquillae* | 446.6 | 776.5 | 341.9 | 79.96 | 821.3 | 0 | 0 |
| *Glyphium elatum* | 639.9 | 543.4 | 264.1 | 27.42 | 0 | 207 | 0 |
| *Pseudotaeniolina globosa* | 664.3 | 475.9 | 324.9 | 132.5 | 0 | 0 | 0 |
| *Gibberella intricans* | 551.2 | 796.7 | 332.6 | 0 | 410.7 | 414 | 0 |
| *Filobasidium magnum* | 2447 | 1060 | 749.2 | 0 | 0 | 907.8 | 0 |
| *Rhodotorula graminis* | 3076 | 2041 | 1078 | 0 | 0 | 887.8 | 0 |
| *Curvibasidium cygneicollum* | 2850 | 1265 | 546.5 | 246.7 | 0 | 350.4 | 0 |
| *Ramularia collo-cygni* | 196 | 282.5 | 96.33 | 123.4 | 410.7 | 0 | 0 |
| *Devriesia pseudoamericana* | 1329 | 1227 | 696.2 | 61.68 | 0 | 350.4 | 0 |
| *Phylliscum demangeonii* | 2161 | 783.5 | 327 | 164.5 | 0 | 0 | 0 |
| *Phoma herbarum* | 254.5 | 616.5 | 227.2 | 109.7 | 0 | 414 | 0 |
| *Filobasidium oeirense* | 255.1 | 684 | 248.6 | 54.85 | 0 | 414 | 0 |
| *Cutaneotrichosporon moniliiforme* | 1287 | 809.8 | 884.2 | 54.85 | 0 | 700.8 | 0 |
| *Pyrenochaetopsis leptospora* | 2560 | 1897 | 1659 | 54.85 | 0 | 0 | 0 |
| *Ascochyta manawaorae* | 535.3 | 572.6 | 371.4 | 54.85 | 0 | 374 | 0 |
| *Umbilicaria calvescens* | 1254 | 276.9 | 377 | 27.42 | 1189 | 0 | 0 |
| *Knufia tsunedae* | 1370 | 392.4 | 759.7 | 27.42 | 0 | 621.1 | 0 |
| *Aspergillus clavatus* | 48.24 | 109.9 | 57.66 | 79.96 | 0 | 1075 | 0 |
| *Myrothecium cinctum* | 1241 | 1372 | 485.6 | 27.42 | 0 | 0 | 0 |
| *Ramularia pratensis* | 891.1 | 934.4 | 288.7 | 27.42 | 0 | 0 | 0 |
| *Fusarium culmorum* | 899.5 | 922.6 | 249.9 | 0 | 410.7 | 0 | 0 |
| *Holtermanniella wattica* | 326.5 | 802.3 | 116.8 | 105.1 | 0 | 1449 | 0 |
| *Extremus antarcticus* | 703.8 | 331.2 | 157.5 | 0 | 0 | 1115 | 0 |
| *Neocatenulostroma abietis* | 2101 | 2146 | 838.9 | 0 | 0 | 414 | 0 |
| *Monographella nivalis* | 1293 | 1709 | 743.8 | 0 | 0 | 700.8 | 0 |
|  |  |  |  |  |  |  |  |
| *Figure S2 Continued* |  |  |  |  |  |  |  |
|  | **Low** | | |  | **Medium** |  | **High** |
| **Fungal Species** | **50%** | **65%** | **70%** | **75%** | **80%** | **85%** | **95%** |
| *Hortaea thailandica* | 1162 | 1031 | 449 | 0 | 0 | 350.4 | 0 |
| *Naganishia globosa* | 1781 | 208 | 774.1 | 105.1 | 0 | 187 | 0 |
| *Botrytis caroliniana* | 978.8 | 874.6 | 359.1 | 52.54 | 0 | 0 | 0 |
| *Teratosphaeria aurantia* | 665.5 | 983.9 | 508.9 | 0 | 0 | 374 | 0 |
| *Phaeotheca salicorniae* | 284.7 | 336.8 | 189.9 | 61.68 | 0 | 0 | 0 |
| *Acremonium tubakii* | 425.2 | 499.5 | 655.5 | 82.27 | 0 | 1051 | 0 |
| *Neophaeosphaeria quadriseptata* | 80.2 | 254.7 | 98.77 | 107.4 | 594.5 | 350.4 | 0 |
| *Filobasidium stepposum* | 1251 | 1188 | 670.7 | 54.85 | 0 | 0 | 0 |
| *Cystofilobasidium macerans* | 782.5 | 731.2 | 225.4 | 54.85 | 0 | 0 | 0 |
| *Coniochaeta polymorpha* | 320 | 219.9 | 321.8 | 54.85 | 0 | 0 | 0 |
| *Alternaria porri* | 56.69 | 68.88 | 99.18 | 79.96 | 0 | 0 | 0 |
| *Cystobasidium slooffiae* | 742.4 | 1012 | 408 | 0 | 879.7 | 0 | 0 |
| *Naganishia randhawae* | 465.6 | 364.6 | 83.73 | 0 | 0 | 414 | 0 |
| *Montagnula jonesii* | 290.1 | 68.88 | 147.8 | 0 | 0 | 207 | 0 |
| *Cystofilobasidium capitatum* | 842.5 | 850.9 | 78.1 | 0 | 0 | 700.8 | 0 |
| *Vermiconia calcicola* | 2259 | 1853 | 944.2 | 0 | 0 | 0 | 0 |
| *Saitozyma paraflava* | 197.5 | 487.7 | 190.6 | 0 | 0 | 0 | 0 |
| *Vishniacozyma dimennae* | 290.4 | 1209 | 132.6 | 0 | 0 | 0 | 0 |
| *Aureobasidium pullulans* | 376 | 464.1 | 165.3 | 0 | 0 | 0 | 0 |
| *Pleopsidium chlorophanum* | 322.9 | 517 | 137.9 | 0 | 0 | 0 | 0 |
| *Stemphylium vesicarium* | 844.9 | 502.4 | 439.9 | 0 | 0 | 0 | 0 |
| *Didymella calidophila* | 768.9 | 303.3 | 302.4 | 0 | 0 | 187 | 0 |
| *Phialocephala fluminis* | 630.5 | 531.6 | 135.4 | 0 | 0 | 0 | 0 |
| *Didymella glomerata* | 740.6 | 759.1 | 1422 | 123.4 | 0 | 0 | 0 |
| *Paradendryphiella arenariae* | 494.5 | 206.6 | 55.23 | 123.4 | 0 | 0 | 0 |
| *Microascus brevicaulis* | 20.5 | 27.83 | 13.7 | 89.1 | 1005 | 0 | 0 |
| *Verrucaria viridula* | 2620 | 1145 | 1218 | 61.68 | 0 | 0 | 0 |
| *Tremella lobariacearum* | 155.6 | 233.1 | 38.67 | 61.68 | 0 | 0 | 0 |
| *Alternaria armoraciae* | 308.8 | 55.65 | 143.1 | 159.9 | 0 | 0 | 0 |
| *Neoascochyta paspali* | 2194 | 877.3 | 83.39 | 54.85 | 0 | 0 | 0 |
| *Neophaeococcomyces catenatus* | 270.8 | 210.8 | 33.04 | 27.42 | 0 | 350.4 | 0 |
| *Meristemomyces frigidus* | 298.8 | 193.4 | 110 | 27.42 | 0 | 0 | 0 |
| *Rhodosporidiobolus colostri* | 965.2 | 518.3 | 45.06 | 0 | 821.3 | 0 | 0 |
| *Alternaria brassicae* | 294 | 137.8 | 167.7 | 0 | 410.7 | 0 | 0 |
| *Hispidoconidioma alpinum* | 162.5 | 210.8 | 175.8 | 52.54 | 410.7 | 0 | 0 |
| *Coniochaeta decumbens* | 1169 | 357.6 | 71.36 | 0 | 0 | 414 | 0 |
| *Cercophora acanthigera* | 633 | 249.1 | 777.9 | 0 | 0 | 207 | 0 |
| *Thanatephorus cucumeris* | 134.8 | 137.8 | 11.27 | 0 | 0 | 394 | 0 |
| *Kurtzmanomyces nectairei* | 424.6 | 490.5 | 120.9 | 0 | 0 | 1051 | 0 |
| *Kondoa aeria* | 943.2 | 178.8 | 253.6 | 0 | 0 | 350.4 | 0 |
|  |  |  |  |  |  |  |  |
| *Figure S2 Continued* |  |  |  |  |  |  |  |
|  | **Low** | | |  | **Medium** |  | **High** |
| **Fungal Species** | **50%** | **65%** | **70%** | **75%** | **80%** | **85%** | **95%** |
| *Wallemia hederae* | 1933 | 1188 | 1192 | 0 | 0 | 0 | 0 |
| *Symmetrospora vermiculata* | 996 | 247.7 | 824.6 | 0 | 0 | 0 | 0 |
| *Coniothyrium multiporum* | 813.6 | 583 | 343.3 | 0 | 0 | 0 | 0 |
| *Tremella exigua* | 193.3 | 496.8 | 118.9 | 0 | 0 | 0 | 0 |
| *Siphula fastigiata* | 111.6 | 336.8 | 140.3 | 0 | 0 | 0 | 0 |
| *Symmetrospora gracilis* | 343.5 | 506.5 | 49.94 | 0 | 0 | 0 | 0 |
| *Drechslera phlei* | 312.7 | 359 | 170.5 | 0 | 0 | 0 | 0 |
| *Exophiala xenobiotica* | 369.1 | 336.8 | 240.2 | 0 | 0 | 0 | 0 |
| *Pseudofusicoccum violaceum* | 118.8 | 152.4 | 156.8 | 0 | 0 | 0 | 0 |
| *Exophiala capensis* | 257.8 | 68.88 | 112.9 | 0 | 0 | 0 | 0 |
| *Papiliotrema nemorosus* | 438.4 | 429.3 | 107.3 | 0 | 0 | 0 | 0 |
| *Buckleyzyma kluyveri-nielii* | 920.6 | 836.3 | 121.7 | 0 | 0 | 0 | 0 |
| *Rhizocarpon lavatum* | 382.6 | 307.5 | 104.8 | 0 | 0 | 0 | 0 |
| *Rhinocladiella fasciculata* | 286.8 | 367.4 | 93.55 | 0 | 0 | 0 | 0 |
| *Unguicularia carestiana* | 515 | 364.6 | 43.96 | 52.54 | 0 | 0 | 0 |
| *Shiraia bambusicola* | 357.7 | 413.3 | 45.06 | 123.4 | 0 | 0 | 0 |
| *Austroafricana keanei* | 292.8 | 1207 | 93.55 | 61.68 | 0 | 0 | 0 |
| *Exophiala moniliae* | 300 | 240.1 | 5.633 | 61.68 | 0 | 0 | 0 |
| *Neoidriella desertorum* | 73.88 | 55.65 | 49.59 | 54.85 | 0 | 207 | 0 |
| *Symmetrospora coprosmae* | 435.7 | 386.9 | 33.8 | 27.42 | 0 | 0 | 0 |
| *Endophoma elongata* | 273.6 | 41.05 | 68.93 | 0 | 1189 | 0 | 0 |
| *Blumeria graminis* | 105.8 | 27.83 | 87.92 | 52.54 | 1189 | 0 | 0 |
| *Caloplaca ferrarii* | 105.2 | 82.11 | 49.59 | 0 | 0 | 207 | 0 |
| *Tremella moriformis* | 268.1 | 82.11 | 71.36 | 0 | 0 | 350.4 | 0 |
| *Arxiella dolichandrae* | 91.38 | 109.9 | 179.3 | 0 | 0 | 0 | 0 |
| *Erythrobasidium hasegawianum* | 454.1 | 278.3 | 292.6 | 0 | 0 | 0 | 0 |
| *Austroafricana parva* | 345 | 260.9 | 258.8 | 0 | 0 | 0 | 0 |
| *Acremonium charticola* | 255.1 | 287.4 | 192.3 | 0 | 0 | 0 | 0 |
| *Cladosporium grevilleae* | 180.6 | 181.6 | 230.6 | 0 | 0 | 0 | 0 |
| *Papiliotrema terrestris* | 24.73 | 401.5 | 52.37 | 0 | 0 | 0 | 0 |
| *Stagonosporopsis dorenboschii* | 884.1 | 900.9 | 61.2 | 0 | 0 | 0 | 0 |
| *Perusta inaequalis* | 291.3 | 587.3 | 33.04 | 0 | 0 | 0 | 0 |
| *Parathyridaria robiniae* | 191.8 | 249.1 | 291.2 | 0 | 0 | 0 | 0 |
| *Solicoccozyma aeria* | 220.4 | 281.1 | 115.3 | 0 | 0 | 0 | 0 |
| *Neodevriesia bulbillosa* | 509.6 | 306.1 | 156.8 | 0 | 0 | 0 | 0 |
| *Schizothecium glutinans* | 214.4 | 82.11 | 112.9 | 0 | 0 | 0 | 0 |
| *Rhizocarpon jemtlandicum* | 144.8 | 41.05 | 112.9 | 0 | 0 | 0 | 0 |
| *Scytinostromella heterogenea* | 397.5 | 183 | 145.6 | 0 | 0 | 0 | 0 |
| *Hypoxylon samuelsii* | 94.69 | 209.4 | 101.6 | 0 | 0 | 0 | 0 |
| *Darksidea epsilon* | 32.87 | 348.6 | 57.66 | 0 | 0 | 0 | 0 |
| *Figure S2 Continued* |  |  |  |  |  |  |  |
|  | **Low** | | |  | **Medium** |  | **High** |
| **Fungal Species** | **50%** | **65%** | **70%** | **75%** | **80%** | **85%** | **95%** |
| *Cupulina montana* | 224.6 | 152.4 | 57.66 | 0 | 0 | 0 | 0 |
| *Rhodotorula diobovata* | 483.4 | 393.8 | 204 | 0 | 0 | 0 | 0 |
| *Microdochium novae-zelandiae* | 411 | 260.9 | 286.3 | 0 | 0 | 0 | 0 |
| *Microdochium paspali* | 421.2 | 310.3 | 110.5 | 0 | 0 | 0 | 0 |
| *Catenulostroma elginense* | 463.8 | 151 | 104.8 | 0 | 0 | 0 | 0 |
| *Acremonium curvulum* | 281.1 | 127.3 | 99.18 | 105.1 | 0 | 0 | 0 |
| *Flavoplaca citrina* | 173.7 | 68.88 | 55.23 | 0 | 0 | 0 | 0 |
| *Dioszegia takashimae* | 200.2 | 251.9 | 11.27 | 0 | 0 | 0 | 0 |
| *Sporobolomyces salicinus* | 134.8 | 96.71 | 181.5 | 0 | 0 | 0 | 0 |
| *Rhodotorula mucilaginosa* | 111 | 168.4 | 175.8 | 0 | 0 | 0 | 0 |
| *Alternaria metachromatica* | 583.8 | 402.9 | 131.9 | 0 | 0 | 0 | 0 |
| *Macroventuria anomochaeta* | 242.7 | 574.8 | 153.6 | 123.4 | 0 | 0 | 0 |
| *Leptodontidium trabinellum* | 186.7 | 278.3 | 71.36 | 54.85 | 0 | 0 | 0 |
| *Cyphellophora vermispora* | 169.8 | 109.9 | 27.4 | 54.85 | 0 | 0 | 0 |
| *Cyberlindnera jadinii* | 278 | 219.9 | 0 | 54.85 | 0 | 0 | 0 |
| *Penicillium phoeniceum* | 40.1 | 41.05 | 43.96 | 27.42 | 0 | 394 | 0 |
| *Fusicolla violacea* | 365.5 | 0 | 24.97 | 27.42 | 0 | 0 | 0 |
| *Setophaeosphaeria badalingensis* | 185.8 | 124.5 | 5.633 | 27.42 | 0 | 0 | 0 |
| *Bryochiton monascus* | 113.7 | 99.51 | 43.96 | 0 | 410.7 | 0 | 0 |
| *Aspergillus candidus* | 80.2 | 109.9 | 57.66 | 0 | 594.5 | 0 | 0 |
| *Ramophialophora humicola* | 73.88 | 55.65 | 225.4 | 0 | 0 | 1242 | 0 |
| *Alfaria acaciae* | 46.14 | 274.1 | 27.4 | 0 | 0 | 414 | 0 |
| *Curvularia lunata* | 297.9 | 83.48 | 137.5 | 0 | 0 | 207 | 0 |
| *Ascochyta medicaginicola* | 127.6 | 111.3 | 192.2 | 0 | 0 | 0 | 0 |
| *Acarospora molybdina* | 177 | 68.88 | 139.9 | 0 | 0 | 0 | 0 |
| *Xenophacidiella pseudocatenata* | 211.7 | 151 | 93.48 | 0 | 0 | 0 | 0 |
| *Vishniacozyma tephrensis* | 327.8 | 55.65 | 93.48 | 0 | 0 | 0 | 0 |
| *Antennariella placitae* | 409.3 | 304.7 | 68.51 | 0 | 0 | 0 | 0 |
| *Podospora intestinacea* | 427 | 82.11 | 258.8 | 0 | 0 | 0 | 0 |
| *Verrucaria muralis* | 108.3 | 294.3 | 60.44 | 0 | 0 | 0 | 0 |
| *Rhodotorula kratochvilovae* | 494.2 | 55.65 | 60.44 | 0 | 0 | 0 | 0 |
| *Cladophialophora chaetospira* | 293.4 | 0 | 142.7 | 52.54 | 0 | 0 | 0 |
| *Drechslera poae* | 422.2 | 139.1 | 480.7 | 0 | 0 | 0 | 0 |
| *Verrucaria macrostoma* | 164.9 | 221.2 | 41.11 | 0 | 0 | 374 | 0 |
| *Candelaria pacifica* | 90.16 | 226.8 | 203.2 | 0 | 0 | 0 | 0 |
| *Bartalinia robillardoides* | 103.4 | 839 | 115.3 | 0 | 0 | 0 | 0 |
| *Penicillium catenatum* | 45.23 | 219.9 | 71.36 | 0 | 0 | 0 | 0 |
| *Verticillium dahliae* | 340.2 | 318 | 71.36 | 0 | 0 | 0 | 0 |
| *Amandinea punctata* | 166.8 | 55.65 | 107.3 | 0 | 0 | 0 | 0 |
| *Phaeophyscia imbricata* | 123.6 | 164.2 | 63.29 | 0 | 0 | 0 | 0 |
| *Pyrenochaeta inflorescentiae* | 273.5 | 55.65 | 101.6 | 0 | 0 | 0 | 0 |
| *Figure S2 Continued* |  |  |  |  |  |  |  |
|  | **Low** | | |  | **Medium** |  | **High** |
| **Fungal Species** | **50%** | **65%** | **70%** | **75%** | **80%** | **85%** | **95%** |
| *Sarcinomyces crustaceus* | 196.6 | 292.9 | 22.53 | 0 | 0 | 0 | 0 |
| *Rhodonia placenta* | 295.8 | 167 | 104.8 | 0 | 0 | 0 | 0 |
| *Cystobasidium lysinophilum* | 191.2 | 111.3 | 275 | 0 | 0 | 0 | 0 |
| *Peniophora nuda* | 1035 | 82.11 | 99.18 | 0 | 0 | 0 | 0 |
| *Cyphellophora laciniata* | 180.9 | 96.71 | 99.18 | 0 | 0 | 0 | 0 |
| *Filobasidium chernovii* | 189.1 | 99.51 | 55.23 | 0 | 0 | 0 | 0 |
| *Leptosphaeria maculans* | 82.02 | 266.5 | 49.59 | 0 | 0 | 0 | 0 |
| *Teratosphaeria zuluensis* | 89.55 | 99.51 | 49.59 | 0 | 0 | 0 | 0 |
| *Cladosporium adianticola* | 960.7 | 137.8 | 5.633 | 0 | 0 | 0 | 0 |
| *Kondoa miscanthi* | 126.6 | 124.5 | 5.633 | 0 | 0 | 0 | 0 |
| *Leptoxyphium madagascariense* | 199.3 | 292.9 | 131.9 | 0 | 0 | 0 | 0 |
| *Candida albicans* | 338 | 391.1 | 87.92 | 0 | 0 | 0 | 0 |
| *Lobothallia alphoplaca* | 50.37 | 151 | 87.92 | 105.1 | 0 | 0 | 0 |
| *Solicoccozyma phenolica* | 489.8 | 249.1 | 87.92 | 0 | 0 | 0 | 0 |
| *Ramimonilia apicalis* | 358.5 | 251.9 | 0 | 0 | 0 | 0 | 0 |
| *Scleroderma albidum* | 0 | 0 | 5.633 | 169.1 | 0 | 207 | 0 |
| *Myriangium citri* | 136.6 | 27.83 | 0 | 54.85 | 0 | 414 | 0 |
| *Trichosporon lactis* | 72.66 | 0 | 104.8 | 54.85 | 0 | 0 | 0 |
| *Dinemasporium japonicum* | 155.3 | 0 | 5.633 | 54.85 | 0 | 0 | 0 |
| *Penicillium cinnamopurpureum* | 52.46 | 0 | 0 | 27.42 | 0 | 621.1 | 0 |
| *Hypoxylon hypomiltum* | 347.7 | 41.05 | 13.7 | 27.42 | 0 | 0 | 0 |
| *Pseudoteratosphaeria ohnowa* | 65.74 | 0 | 181.5 | 27.42 | 0 | 0 | 0 |
| *Neophaeococcomyces aloes* | 171.9 | 55.65 | 5.633 | 27.42 | 0 | 0 | 0 |
| *Tremella indecorata* | 81.41 | 0 | 43.96 | 27.42 | 0 | 0 | 0 |
| *Aculeata aquatica* | 94.99 | 27.83 | 5.633 | 0 | 410.7 | 0 | 0 |
| *Alternaria eureka* | 170.1 | 0 | 41.11 | 0 | 594.5 | 0 | 0 |
| *Pectenia plumbea* | 151.7 | 27.83 | 43.96 | 0 | 0 | 414 | 0 |
| *Sarocladium subulatum* | 50.37 | 254.7 | 0 | 0 | 0 | 414 | 0 |
| *Angustimassarina acerina* | 553.7 | 0 | 27.4 | 0 | 0 | 207 | 0 |
| *Epicoccum henningsii* | 97.09 | 41.05 | 11.27 | 0 | 0 | 207 | 0 |
| *Alternaria pseudorostrata* | 201.5 | 68.88 | 54.81 | 0 | 0 | 0 | 0 |
| *Fusidium griseum* | 87.76 | 55.65 | 52.37 | 0 | 0 | 0 | 0 |
| *Umbilicaria lyngei* | 61.82 | 0 | 90.7 | 0 | 0 | 0 | 0 |
| *Phaeosaccardinula ficus* | 144.7 | 124.5 | 41.11 | 0 | 0 | 0 | 0 |
| *Pseudorobillarda phragmitis* | 107.4 | 0 | 82.63 | 0 | 0 | 0 | 0 |
| *Hormonema viticola* | 33.48 | 111.3 | 33.04 | 0 | 0 | 0 | 0 |
| *Bannoa ogasawarensis* | 103.1 | 82.11 | 159.3 | 0 | 0 | 0 | 0 |
| *Devriesia strelitziicola* | 286.8 | 109.9 | 27.4 | 0 | 0 | 0 | 0 |
| *Ampelomyces quisqualis* | 489.1 | 55.65 | 27.4 | 0 | 0 | 0 | 0 |
| *Naganishia friedmannii* | 410.7 | 55.65 | 27.4 | 0 | 0 | 0 | 0 |
| *Oliveonia pauxilla* | 205.3 | 99.51 | 57.66 | 0 | 0 | 0 | 0 |
| *Figure S2 Continued* |  |  |  |  |  |  |  |
|  | **Low** | | |  | **Medium** |  | **High** |
| **Fungal Species** | **50%** | **65%** | **70%** | **75%** | **80%** | **85%** | **95%** |
| *Devriesia fraserae* | 49.76 | 140.6 | 13.7 | 0 | 0 | 0 | 0 |
| *Neodevriesia lagerstroemiae* | 160.4 | 152.4 | 13.7 | 0 | 0 | 0 | 0 |
| *Cryptococcus saitoi* | 54.29 | 41.05 | 13.7 | 0 | 0 | 187 | 0 |
| *Schizangiella serpentis* | 83.84 | 127.3 | 50.7 | 0 | 0 | 0 | 0 |
| *Ceratobasidium anceps* | 1359 | 199 | 133 | 0 | 0 | 0 | 0 |
| *Tausonia pullulans* | 124.2 | 82.11 | 28.16 | 52.54 | 0 | 0 | 0 |
| *Rhizophlyctis rosea* | 28.95 | 27.83 | 192.7 | 0 | 0 | 0 | 0 |
| *Cutaneotrichosporon dermatis* | 41.31 | 139.1 | 104.8 | 0 | 0 | 0 | 0 |
| *Candida sake* | 36.79 | 155.2 | 16.9 | 0 | 0 | 0 | 0 |
| *Sakaguchia lamellibrachiae* | 130.9 | 55.65 | 231.1 | 0 | 0 | 0 | 0 |
| *Leucosporidium yakuticum* | 50.37 | 199 | 99.18 | 0 | 0 | 0 | 0 |
| *Endosporium aviarium* | 213.5 | 55.65 | 99.18 | 0 | 0 | 0 | 0 |
| *Dictyosporium heptasporum* | 65.74 | 123.2 | 55.23 | 0 | 0 | 0 | 0 |
| *Epicoccum brasiliense* | 1728 | 41.05 | 55.23 | 0 | 0 | 0 | 0 |
| *Rachicladosporium luculiae* | 24.73 | 55.65 | 55.23 | 0 | 0 | 0 | 0 |
| *Fenestella ostryae* | 137.8 | 82.11 | 11.27 | 0 | 0 | 0 | 0 |
| *Flavoplaca coronata* | 155.9 | 68.88 | 11.27 | 0 | 0 | 0 | 0 |
| *Septoria cretae* | 223.8 | 152.4 | 11.27 | 0 | 0 | 0 | 0 |
| *Buckleyzyma phyllomatis* | 125.2 | 82.11 | 49.59 | 0 | 0 | 0 | 0 |
| *Thelebolus spongiae* | 256.6 | 0 | 49.59 | 0 | 0 | 0 | 0 |
| *Knufia endospora* | 111 | 193.4 | 5.633 | 0 | 0 | 0 | 0 |
| *Pyrenochaeta nobilis* | 111 | 124.5 | 5.633 | 0 | 0 | 0 | 0 |
| *Rhizophydium planktonicum* | 72.97 | 41.05 | 5.633 | 0 | 0 | 0 | 0 |
| *Paraconiothyrium archidendri* | 111.3 | 27.83 | 5.633 | 0 | 0 | 0 | 0 |
| *Cystofilobasidium ferigula* | 57.29 | 302 | 1055 | 0 | 0 | 0 | 0 |
| *Sphaerellopsis filum* | 180.6 | 165.6 | 131.9 | 0 | 0 | 0 | 0 |
| *Caloplaca arcis* | 53.68 | 181.6 | 87.92 | 0 | 0 | 0 | 0 |
| *Dioszegia zsoltii* | 45.23 | 254.7 | 87.92 | 0 | 0 | 0 | 0 |
| *Elasticomyces elasticus* | 56.69 | 0 | 87.92 | 0 | 0 | 187 | 0 |
| *Knufia aspidiotus* | 600.4 | 369.5 | 43.96 | 0 | 0 | 0 | 0 |
| *Vishniacozyma psychrotolerans* | 100.7 | 82.11 | 43.96 | 0 | 0 | 0 | 0 |
| *Ceratobasidium cornigerum* | 418.2 | 41.05 | 43.96 | 0 | 0 | 0 | 0 |
| *Laetisaria fuciformis* | 231.6 | 96.71 | 43.96 | 0 | 0 | 0 | 0 |
| *Kurtzmanomyces insolitus* | 32.87 | 68.88 | 43.96 | 0 | 0 | 0 | 0 |
| *Mycoarthris corallina* | 1150 | 274.1 | 0 | 0 | 0 | 0 | 0 |
| *Rhodotorula dairenensis* | 90.77 | 250.5 | 0 | 0 | 0 | 0 | 0 |
| *Alternaria infectoria* | 202.9 | 151 | 0 | 0 | 0 | 0 | 0 |
| *Recurvomyces mirabilis* | 228.6 | 137.8 | 0 | 0 | 0 | 0 | 0 |
| *Septoriella phragmitis* | 111 | 221.2 | 0 | 0 | 0 | 0 | 0 |
| *Sarocladium summerbellii* | 98.3 | 789 | 0 | 0 | 0 | 0 | 0 |
| *Meyerozyma caribbica* | 94.38 | 196.2 | 0 | 0 | 0 | 0 | 0 |
| *Figure S2 Continued* |  |  |  |  |  |  |  |
|  | **Low** | | |  | **Medium** |  | **High** |
| **Fungal Species** | **50%** | **65%** | **70%** | **75%** | **80%** | **85%** | **95%** |
| *Filobasidium wieringae* | 213.2 | 481.5 | 0 | 0 | 0 | 0 | 0 |

**Figure S2.** Heatmap displaying abundant fungal species found in >20% of the CA dust embedded in carpet A samples from the moisture availability experiments where samples were incubated at 50%, 65%, 70%, 75%, 80%, 85%, and 95% continuously for 4 weeks. Darker colors indicate higher spore equivalent/mg filter dust determined from qPCR.

|  | **Low** | | | **Medium** | | | **High** |
| --- | --- | --- | --- | --- | --- | --- | --- |
| **Fungal Genus** | **50%** | **65%** | **70%** | **75%** | **80%** | **85%** | **95%** |
| *Penicillium* | 6557.1435 | 24655.816 | 14494.244 | 2602.4096 | 2479.4145 | 21344.975 | 4665531.2 |
| *Aspergillus* | 379086.77 | 2406253.7 | 1059108.6 | 859965.35 | 9320535.8 | 16214139 | 2897095.6 |
| *Wallemia* | 328756.36 | 845721.34 | 266194.77 | 4713041.6 | 9659881.4 | 13998052 | 351195.09 |
| *Cladosporium* | 81847.5369 | 52447.8733 | 32265.24 | 2609.23673 | 5567.13969 | 11853.9032 | 17372.1998 |
| *Alternaria* | 16785.679 | 10591.794 | 6614.048 | 685.5079 | 1826.5298 | 1385.5034 | 9875.9209 |
| *Candida* | 14029.1365 | 14450.9728 | 5484.70995 | 25565.123 | 70280.44 | 59930.6318 | 4777.56395 |
| *Acremonium* | 1295.72098 | 1261.4286 | 1181.64939 | 187.349188 | 0 | 1051.1459 | 1272.9474 |
| *Naganishia* | 7544.1002 | 4823.8444 | 2547.3992 | 593.99547 | 1189.0357 | 601.04321 | 837.4978 |
| *Exophiala* | 5352.0163 | 1682.5173 | 893.69263 | 61.680349 | 0 | 414.04856 | 717.40857 |
| *Phaeotheca* | 350.09837 | 405.65898 | 321.75929 | 237.57919 | 0 | 560.98395 | 690.98862 |
| *Epicoccum* | 30683.3126 | 28675.9901 | 14963.3874 | 2759.92673 | 6659.87845 | 10104.5618 | 424.754153 |
| *Talaromyces* | 326.259758 | 881.57283 | 255.384481 | 89.10465 | 594.517854 | 1321.83677 | 424.754153 |
| *Didymella* | 13623.289 | 9581.646 | 19770.139 | 893.35464 | 5481.1872 | 2296.8693 | 367.10847 |
| *Knufia* | 4835.8482 | 2632.19405 | 1414.46025 | 267.31395 | 0 | 1552.46838 | 345.494311 |
| *Nigrospora* | 16650.831 | 17516.7647 | 10020.9018 | 1567.41219 | 594.517854 | 1202.08642 | 67.249333 |
| *Phaeosphaeria* | 1340.9498 | 743.087 | 752.98037 | 82.272904 | 594.51785 | 414.04856 | 40.829386 |
| *Mycosphaerella* | 37836.235 | 23662.643 | 19872.461 | 1098.8886 | 4305.9443 | 6007.7132 | 26.419947 |
| *Ramularia* | 1208.9259 | 1395.6873 | 429.01238 | 150.785 | 410.67065 | 0 | 26.419947 |
| *Neodevriesia* | 1742.97656 | 1227.29817 | 715.642417 | 116.528951 | 0 | 0 | 26.4199474 |
| *Neoascochyta* | 4702.51477 | 1166.09496 | 330.589183 | 54.8486025 | 0 | 414.048556 | 26.4199474 |
| *Hypoxylon* | 529.50375 | 373.65706 | 140.28975 | 79.962443 | 0 | 0 | 26.419947 |
| *Hamigera* | 104.623972 | 292.924062 | 203.927065 | 116.528951 | 2421.04767 | 2503.89356 | 21.6141573 |
| *Aureobasidium* | 6183.6774 | 3565.8661 | 2530.2731 | 420.40709 | 0 | 621.07283 | 14.409438 |
| *Phoma* | 7164.0696 | 3266.0712 | 1750.5388 | 301.66731 | 0 | 1576.0757 | 14.409438 |
| *Myrothecium* | 1240.5684 | 1372.1396 | 485.61091 | 27.424301 | 0 | 0 | 14.409438 |
| *Vishniacozyma* | 38677.798 | 24113.001 | 10548.096 | 1283.9273 | 4020.754 | 3017.6628 | 7.2047191 |
| *Coniosporium* | 14205.8345 | 12974.5226 | 4357.1259 | 635.18058 | 1189.03571 | 4275.83303 | 7.2047191 |
| *Pseudopithomyces* | 10557.7181 | 6978.46647 | 3339.1234 | 712.834881 | 1034.37192 | 1178.47908 | 7.2047191 |
| *Xenodidymella* | 2930.71808 | 2293.26485 | 1226.63866 | 598.61639 | 0 | 1261.74789 | 7.2047191 |
| *Verrucaria* | 3640.17412 | 2158.1995 | 1491.15914 | 61.6803488 | 0 | 581.013581 | 7.2047191 |
| *Boeremia* | 629.30514 | 667.95529 | 264.78503 | 0 | 2053.3533 | 0 | 7.2047191 |
| *Fusarium* | 1940.4438 | 2223.7867 | 382.4688 | 109.6972 | 1599.7064 | 3527.427 | 0 |
| *Gibberella* | 2826.82823 | 2553.48495 | 987.438474 | 379.221981 | 410.670654 | 828.097112 | 0 |
| *Filobasidium* | 4848.73014 | 3540.94426 | 1895.0926 | 109.697205 | 439.854069 | 1321.83677 | 0 |
| *Pyrenochaetopsis* | 3932.1246 | 3167.3395 | 2061.0273 | 82.272904 | 594.51785 | 1114.8125 | 0 |
| *Microdochium* | 3503.8893 | 4616.5301 | 1574.8458 | 109.6972 | 4577.3418 | 700.76394 | 0 |
| *Phaeococcomyces* | 3894.99249 | 2929.13602 | 1285.6313 | 269.622092 | 1783.55356 | 414.048556 | 0 |
| *Tremella* | 821.391196 | 1510.60304 | 353.046706 | 89.10465 | 0 | 350.381968 | 0 |
| *Rhodotorula* | 6681.1918 | 4746.61403 | 2481.59046 | 27.4243012 | 0 | 1822.73185 | 0 |
| *Neonectria* | 404.972351 | 711.809515 | 352.703332 | 116.528951 | 0 | 350.381968 | 0 |
| *Papiliotrema* | 1083.98751 | 2137.40014 | 535.963973 | 123.360698 | 0 | 1823.15925 | 0 |
| *Lapidomyces* | 2861.9164 | 2922.2652 | 1488.8716 | 228.43698 | 0 | 414.04856 | 0 |
| *Buckleyzyma* | 2394.66606 | 1497.32427 | 473.732513 | 61.6803488 | 879.708138 | 350.381968 | 0 |
| *Teratosphaeria* | 944.44266 | 1471.6481 | 564.0868 | 0 | 439.85407 | 373.9893 | 0 |
| *Plectosphaerella* | 454.713784 | 776.515397 | 341.855059 | 79.9624434 | 821.341308 | 0 | 0 |
| *Articulospora* | 1533.7001 | 1827.0748 | 740.53592 | 123.3607 | 0 | 207.02428 | 0 |
| *Sarocladium* | 546.0985 | 1453.5448 | 126.58789 | 0 | 0 | 764.43052 | 0 |
| *Pseudotaeniolina* | 664.298824 | 475.913847 | 324.882017 | 132.500586 | 0 | 0 | 0 |
| *Glyphium* | 639.888787 | 543.420371 | 264.098281 | 27.4243012 | 0 | 207.024278 | 0 |
| *Saitozyma* | 382.622236 | 1468.07068 | 394.495648 | 61.6803488 | 439.854069 | 0 | 0 |

**Figure S3.** Heatmap displaying the 50 most abundant fungal genera in the CA dust embedded in carpet samples from the moisture availability experiments where samples were incubated at 50%, 65%, 70%, 75%, 80%, 85%, and 95% continuously for 4 weeks. Darker colors indicate higher spore equivalent/mg filter dust determined from qPCR.

|  | **Low** | | | | | **Medium** | **High** |
| --- | --- | --- | --- | --- | --- | --- | --- |
| **Fungal Species** | **50%** | **65%** | **70%** | **75%** | **80%** | **85%** | **95%** |
| *Penicillium citrinum* | 0 | 1.06122 | 44.2211 | 15.962 | 3.92684 | 2193.83 | 7923371 |
| *Cladosporium sphaerospermum* | 5.28864 | 10.5347 | 14.2719 | 2.21375 | 3.42676 | 0 | 1269194 |
| *Aspergillus hongkongensis* | 0 | 0.01701 | 10.1726 | 7.34676 | 2.83763 | 0 | 1193722 |
| *Cladosporium ramotenellum* | 0.28395 | 2.06808 | 189.787 | 55.815 | 42.6103 | 23.384 | 876239 |
| *Cladosporium dominicanum* | 0 | 6.419 | 19.1732 | 19.2485 | 20.7079 | 3650.49 | 526046 |
| *Mycosphaerella tassiana* | 84.2278 | 36.4957 | 475.18 | 191.975 | 260.136 | 3821.36 | 514105 |
| *Cladosporium delicatulum* | 146.343 | 75.585 | 846.677 | 347.185 | 375.209 | 5778.65 | 468148 |
| *Penicillium kongii* | 4.40128 | 1.7687 | 4.12364 | 2.05042 | 0.73204 | 957.297 | 315820 |
| *Cladosporium halotolerans* | 4.18832 | 3.45246 | 5.5642 | 8.91663 | 17.3727 | 2260.2 | 295859 |
| *Penicillium chrysogenum* | 17.3922 | 2.61592 | 9.40185 | 0.17475 | 3.45975 | 442.719 | 189980 |
| *Alternaria alternata* | 140.983 | 2.5819 | 205.034 | 45.0601 | 11.3373 | 14687.4 | 152064 |
| *Penicillium brevicompactum* | 0.92285 | 0.35374 | 8.99399 | 0.21608 | 0.96606 | 15.7829 | 68411.8 |
| *Penicillium gladioli* | 2.05866 | 0.23812 | 1.2704 | 0.0307 | 0.6719 | 38.835 | 30717.5 |
| *Stemphylium vesicarium* | 0 | 1.06122 | 18.3336 | 87.9292 | 29.9474 | 0 | 17017 |
| *Toxicocladosporium irritans* | 14.9431 | 0 | 30.9878 | 44.2191 | 38.5105 | 0 | 15170.1 |
| *Talaromyces minioluteus* | 0 | 0 | 8.13805 | 0.89502 | 0.93685 | 1871.85 | 14859.7 |
| *Penicillium polonicum* | 0 | 0 | 0.10173 | 0.04178 | 0.73901 | 0 | 12911.2 |
| *Penicillium bialowiezense* | 0 | 0 | 1.32818 | 0.14405 | 0 | 0 | 8649.98 |
| *Paradendryphiella arenariae* | 0 | 6.66747 | 42.8108 | 25.9638 | 23.6447 | 0 | 7879.17 |
| *Cladosporium fusiforme* | 0 | 0 | 1.55446 | 2.86659 | 1.26237 | 0 | 6054.24 |
| *Penicillium sclerotigenum* | 0 | 0.05103 | 0.52206 | 0.14405 | 0 | 0 | 6041.15 |
| *Alternaria brassicae* | 5.35963 | 0.05103 | 6.62172 | 1.24452 | 0.95835 | 442.719 | 6036.13 |
| *Alternaria metachromatica* | 0.03549 | 0 | 65.388 | 62.8654 | 38.8918 | 0 | 6011.97 |
| *Epicoccum nigrum* | 190.391 | 132.094 | 128.614 | 1009.87 | 629.825 | 10352.9 | 5377.72 |
| *Wallemia tropicalis* | 0 | 0 | 241.128 | 42.095 | 87.8928 | 2951.5 | 4937.46 |
| *Cladosporium aphidis* | 0.03549 | 0 | 5.94255 | 14.6926 | 2.55106 | 0 | 4491.17 |
| *Wallemia muriae* | 12.7069 | 0.47625 | 112.651 | 2.59298 | 38.715 | 1266.27 | 4456.57 |
| *Cladosporium grevilleae* | 0 | 0 | 2.81761 | 2.32372 | 2.75907 | 0 | 4287.88 |
| *Wallemia canadensis* | 0 | 0 | 82.3053 | 0.32228 | 46.2307 | 15.617 | 4033.08 |
| *Penicillium simplicissimum* | 0 | 0 | 0.30518 | 0 | 0 | 0 | 3804.08 |
| *Ramularia pratensis* | 0 | 0 | 33.4093 | 13.3018 | 32.4278 | 0.04149 | 3126.3 |
| *Candida parapsilosis* | 1.45526 | 8.90497 | 156.82 | 68.924 | 57.394 | 2043.34 | 2682.35 |
| *Periconia byssoides* | 0.14198 | 0 | 3.34528 | 7.66872 | 26.137 | 0 | 2535.17 |
| *Cladosporium velox* | 0 | 0.17687 | 0 | 0.14405 | 0 | 0 | 2487.94 |
| *Alternaria infectoria* | 0 | 0 | 18.608 | 13.4464 | 9.66608 | 0 | 2079.92 |
| *Penicillium decumbens* | 0 | 0 | 0.20345 | 0 | 0.07495 | 0 | 1528.61 |
| *Toxicocladosporium strelitziae* | 2.05866 | 0 | 8.55839 | 5.17182 | 3.26907 | 0 | 1363.63 |
| *Penicillium paczoskii* | 0 | 0 | 0.71208 | 0 | 0.26232 | 38.835 | 1262.53 |
| *Penicillium expansum* | 0 | 1.34025 | 2.00571 | 5.61811 | 0.45404 | 7.767 | 1178.05 |
| *Penicillium thymicola* | 0.03549 | 0 | 0 | 0.21608 | 0.24401 | 23.301 | 1135.84 |
| *Rachicladosporium cboliae* | 0 | 0 | 0.75793 | 0 | 3.85981 | 0 | 1102.59 |
| *Penicillium aurantiogriseum* | 0 | 0 | 0 | 0 | 1.2462 | 0 | 1102.59 |
| *Penicillium neocrassum* | 0 | 0 | 0 | 0 | 0 | 0.16595 | 1013.09 |
| *Vishniacozyma carnescens* | 0 | 0.01701 | 40.6562 | 11.4523 | 31.829 | 0 | 951.679 |
| *Aureobasidium namibiae* | 6.81489 | 1.52724 | 40.9058 | 23.812 | 36.9038 | 1.82549 | 892.849 |
| *Aspergillus ellipticus* | 0 | 0 | 0.20345 | 0 | 0.16692 | 0.08298 | 892.849 |
| *Penicillium glabrum* | 0 | 0 | 0.61035 | 0 | 0.11242 | 124.272 | 848.063 |
| *Alternaria terricola* | 0 | 0.01701 | 0 | 4.86178 | 7.75702 | 0 | 780.283 |
| *Xenodidymella humicola* | 0 | 0 | 0.4069 | 0.07203 | 235.21 | 3650.49 | 640.832 |
| *Kondoa aeria* | 0 | 0 | 49.001 | 30.4782 | 11.2324 | 0.04149 | 621.694 |
| *Curvularia neergaardii* | 0 | 0.35374 | 0.41245 | 0.52425 | 0.46322 | 31.068 | 582.002 |
| *Aspergillus ruber* | 45.2906 | 0.17687 | 34.5867 | 0.06396 | 15.469 | 357.904 | 582.002 |
| *Microdochium phragmitis* | 0 | 0 | 19.9382 | 8.71772 | 0.11242 | 0 | 537.216 |
| *Cladosporium cladosporioides* | 0.10648 | 0.27892 | 0.46448 | 0.17475 | 0.14641 | 0 | 524.464 |
| *Penicillium ochrochloron* | 0 | 0.17687 | 1.1516 | 0 | 0.03747 | 0 | 463.051 |
| *Cladosporium exasperatum* | 0 | 0.19388 | 0.11516 | 0.07203 | 0 | 0 | 431.089 |
| *Vishniacozyma victoriae* | 43.3384 | 30.5204 | 439.046 | 83.1422 | 130.679 | 1809.71 | 418.265 |
| *Didymella gardeniae* | 0 | 14.8827 | 56.9581 | 127.043 | 8.4691 | 0 | 414.463 |
| *Claviceps grohii* | 0 | 9.5174 | 255.559 | 78.2442 | 61.0406 | 0 | 358.144 |
| *Fumiglobus pieridicola* | 0 | 0 | 0.20345 | 6.36908 | 3.74645 | 7.80849 | 358.144 |
| *Alternaria pseudorostrata* | 0 | 0 | 5.41833 | 0.08311 | 0 | 0 | 358.144 |
| *Neocatenulostroma abietis* | 0 | 0 | 10.1726 | 50.121 | 9.33862 | 0 | 342.809 |
| *Cystofilobasidium macerans* | 0 | 2.12244 | 147.154 | 24.5429 | 189.699 | 2788.35 | 310.847 |
| *Alternaria eureka* | 0 | 0 | 0 | 25.3646 | 7.66197 | 7.767 | 300.606 |
| *Penicillium catenatum* | 0 | 0 | 12.6811 | 0 | 2.77639 | 0 | 240.485 |
| *Torula herbarum* | 0 | 13.9727 | 46.3427 | 17.406 | 7.69525 | 3666.02 | 207.231 |
| *Figure S4 Continued* |  |  |  |  |  |  |  |
|  | **Low** | | | | | **Medium** | **High** |
| **Fungal Species** | **50%** | **65%** | **70%** | **75%** | **80%** | **85%** | **95%** |
| *Rhodotorula graminis* | 44.9002 | 0 | 14.4294 | 31.6608 | 4.44777 | 0 | 179.072 |
| *Pleopsidium chlorophanum* | 0 | 0 | 2.47787 | 1.71432 | 4.76936 | 0 | 179.072 |
| *Wallemia sebi* | 0 | 0 | 1.11898 | 0.2562 | 0.44969 | 0 | 179.072 |
| *Phoma saxea* | 0.10648 | 0 | 0 | 0.13342 | 3.67244 | 0 | 179.072 |
| *Aspergillus niger* | 0 | 0 | 0.11516 | 0 | 0 | 31.1095 | 179.072 |
| *Alternaria mimicula* | 0 | 0 | 0 | 0.10318 | 0.03747 | 0 | 179.072 |
| *Alternaria oregonensis* | 0.03549 | 0 | 0.4069 | 0.21608 | 1.17871 | 0 | 163.737 |
| *Epicoccum plurivorum* | 0 | 0 | 13.67 | 4.35886 | 0 | 4551.46 | 120.242 |
| *Austroafricana keanei* | 0 | 0 | 0.42034 | 0.26684 | 0.13508 | 0 | 103.616 |
| *Phaeosphaeria caricicola* | 0 | 0 | 4.20721 | 2.03682 | 4.24581 | 0 | 103.616 |
| *Gibberella pulicaris* | 0.03549 | 0.17687 | 0.05758 | 0 | 0.16122 | 0 | 103.616 |
| *Hamigera insecticola* | 0 | 0.17687 | 0 | 0.01109 | 0 | 31.1095 | 103.616 |
| *Aspergillus penicillioides* | 0 | 0 | 13.6254 | 0 | 4.5639 | 0 | 103.616 |
| *Antennariella placitae* | 0 | 0 | 13.1637 | 0.14405 | 0.0488 | 0 | 103.616 |
| *Polymeridium subcinereum* | 0 | 0 | 0.10173 | 0.10272 | 0 | 0 | 103.616 |
| *Ganoderma australe* | 0 | 0 | 60.2197 | 17.5417 | 26.9453 | 0 | 60.1211 |
| *Venturia maculiformis* | 9.68992 | 0 | 22.4866 | 12.3225 | 3.1591 | 497.088 | 60.1211 |
| *Symmetrospora vermiculata* | 0 | 0 | 25.5386 | 14.2323 | 10.1601 | 0 | 60.1211 |
| *Ganoderma pfeifferi* | 0 | 0 | 104.301 | 49.4725 | 51.7335 | 0 | 60.1211 |
| *Itersonilia perplexans* | 0 | 0 | 26.7538 | 42.9819 | 9.68132 | 0 | 60.1211 |
| *Exidia recisa* | 0 | 0 | 6.43551 | 3.68354 | 0 | 0 | 60.1211 |
| *Botrytis caroliniana* | 220.313 | 94.3404 | 423.227 | 185.841 | 195.054 | 25204 | 0 |
| *Podosphaera leucotricha* | 97.0057 | 65.2277 | 1995.99 | 639.373 | 523.8 | 1571.54 | 0 |
| *Botrytis cinerea* | 0.49692 | 0.92526 | 32.8296 | 1.46543 | 4.05149 | 209.709 | 0 |
| *Coniosporium apollinis* | 0.49692 | 0 | 74.6818 | 14.8835 | 20.5201 | 240.777 | 0 |
| *Aureobasidium pullulans* | 0.53241 | 0.01701 | 4.86916 | 1.10989 | 8.64308 | 0.04149 | 0 |
| *Rhodotorula mucilaginosa* | 0.07099 | 0 | 17.6367 | 4.41099 | 5.01781 | 93.204 | 0 |
| *Rhodotorula dairenensis* | 0.03549 | 0 | 15.7864 | 5.73757 | 3.31837 | 124.272 | 0 |
| *Alternaria porri* | 0.10648 | 0.38776 | 0.41245 | 0.28811 | 0.66406 | 7.767 | 0 |
| *Filobasidium stepposum* | 47.1363 | 0.11906 | 122.094 | 65.0413 | 24.7482 | 0 | 0 |
| *Blumeria graminis* | 6.38896 | 4.21819 | 106.907 | 61.7016 | 115.838 | 0 | 0 |
| *Trichoderma rifaii* | 0 | 1.83673 | 4.15518 | 3.3947 | 0.84801 | 0 | 0 |
| *Taphrina deformans* | 42.7705 | 3.71427 | 30.9819 | 19.8166 | 94.2723 | 0 | 0 |
| *Umbilicaria calvescens* | 0.85186 | 0 | 24.9937 | 3.79715 | 8.68513 | 0 | 0 |
| *Glyphium elatum* | 8.09268 | 0 | 38.6575 | 1.45496 | 6.33969 | 0 | 0 |
| *Filobasidium oeirense* | 31.0219 | 2.61936 | 40.5633 | 14.3503 | 19.2898 | 0 | 0 |
| *Leptodontidium trabinellum* | 48.0592 | 0 | 88.6298 | 59.3401 | 7.71344 | 2733.98 | 0 |
| *Trametes versicolor* | 0 | 0.01701 | 78.6335 | 54.3179 | 34.1377 | 0.04149 | 0 |
| *Verrucocladosporium dirinae* | 0 | 0 | 0.72551 | 1.03282 | 2.03757 | 46.602 | 0 |
| *Grammothele lineata* | 0.03549 | 0 | 0.93067 | 0.44038 | 0.3508 | 0 | 0 |
| *Trichoderma virens* | 0 | 0.70748 | 2.18209 | 0.68837 | 0.3508 | 0 | 0 |
| *Cystotheca lanestris* | 0 | 4.47332 | 134.244 | 97.9038 | 71.5236 | 0 | 0 |
| *Erysiphe euonymicola* | 0 | 0.19388 | 10.6969 | 0.46165 | 4.96705 | 0 | 0 |
| *Peziza moseri* | 0 | 3.59554 | 0.31861 | 5.27756 | 1.9832 | 2508.74 | 0 |
| *Pseudopithomyces chartarum* | 0 | 0.70748 | 31.1548 | 125.554 | 74.3854 | 3324.27 | 0 |
| *Phaeococcomyces eucalypti* | 32.7612 | 0 | 33.7074 | 50.4303 | 9.51312 | 0 | 0 |
| *Rhinocladiella fasciculata* | 0 | 0 | 2.10743 | 0.17475 | 5.42978 | 62.136 | 0 |
| *Buckleyzyma kluyveri-nielii* | 0 | 0 | 35.151 | 45.2683 | 25.4927 | 7.767 | 0 |
| *Rhizocarpon jemtlandicum* | 0.03549 | 0 | 1.76195 | 0.86493 | 3.52576 | 0 | 0 |
| *Rhizocarpon lavatum* | 0 | 0 | 19.4008 | 0.82948 | 2.15005 | 0 | 0 |
| *Acanthophysellum lividocoeruleum* | 0 | 0 | 16.6867 | 9.65406 | 25.1432 | 0 | 0 |
| *Podosphaera pannosa* | 0 | 0 | 98.6194 | 37.0752 | 156.523 | 0 | 0 |
| *Venturia populina* | 0.39044 | 0 | 6.32035 | 3.13043 | 0.63792 | 85.437 | 0 |
| *Torula goaensis* | 0.03549 | 1.41496 | 52.118 | 26.9974 | 0.14641 | 4582.53 | 0 |
| *Claviceps zizaniae* | 0 | 0.38776 | 3.4431 | 0.64704 | 0.33384 | 0 | 0 |
| *Torula masonii* | 34.7843 | 0.53061 | 9.12459 | 0.2562 | 7.85718 | 147.573 | 0 |
| *Tricharina praecox* | 37.056 | 5.01761 | 12.0036 | 16.9264 | 53.2433 | 7471.85 | 0 |
| *Knufia marmoricola* | 0 | 0 | 28.5241 | 0.92089 | 3.846 | 0.95423 | 0 |
| *Knufia mediterranea* | 0 | 0.01701 | 6.19303 | 0 | 24.2016 | 0 | 0 |
| *Saitozyma podzolica* | 0 | 0.34018 | 4.86738 | 0.75097 | 0.20439 | 0 | 0 |
| *Neodevriesia stirlingiae* | 0 | 0.70748 | 67.3768 | 2.477 | 5.61404 | 0 | 0 |
| *Phialocephala fluminis* | 0 | 0 | 39.2403 | 0 | 2.20247 | 1079.61 | 0 |
| *Pseudotaeniolina globosa* | 0 | 0 | 89.0626 | 279.525 | 133.737 | 0 | 0 |
| *Neodevriesia lagerstroemiae* | 0 | 4.83051 | 1.52588 | 9.44664 | 3.89212 | 0 | 0 |
| *Sporobolomyces salicinus* | 0 | 0 | 4.99799 | 6.36357 | 1.88492 | 0 | 0 |
| *Epicoccum brasiliense* | 0.24846 | 0.01701 | 1.32243 | 0.3495 | 2.25278 | 0 | 0 |
| *Coprinellus radians* | 0 | 0 | 10.638 | 3.6236 | 4.87492 | 0 | 0 |
| *Figure S4 Continued* |  |  |  |  |  |  |  |
|  | **Low** | | | | | **Medium** | **High** |
| **Fungal Species** | **50%** | **65%** | **70%** | **75%** | **80%** | **85%** | **95%** |
| *Coprinellus micaceus* | 0 | 0 | 21.2355 | 18.9178 | 18.7264 | 0 | 0 |
| *Panaeolus fimicola* | 0 | 8.92198 | 10.2397 | 0 | 56.0461 | 0 | 0 |
| *Cystotheca wrightii* | 0 | 0 | 18.8652 | 8.58525 | 26.2678 | 0 | 0 |
| *Erysiphe nothofagi* | 0.07099 | 0.01701 | 0.31861 | 0.21608 | 0.59836 | 0 | 0 |
| *Agrocybe praecox* | 0 | 0 | 3.49835 | 4.09008 | 4.01837 | 0 | 0 |
| *Alternaria rosae* | 13.4168 | 0 | 11.6984 | 4.77143 | 0.55868 | 0 | 0 |
| *Vishniacozyma dimennae* | 0 | 0 | 70.3825 | 31.2644 | 0.61661 | 0 | 0 |
| *Phaeococcomyces mexicanus* | 0 | 0 | 35.3409 | 21.3529 | 22.1669 | 0 | 0 |
| *Buckleyzyma aurantiaca* | 0 | 0 | 14.0209 | 18.7291 | 23.2531 | 0 | 0 |
| *Knufia perforans* | 0 | 0 | 4.8098 | 0.15514 | 5.84593 | 7.89146 | 0 |
| *Devriesia pseudoamericana* | 0 | 0.15308 | 7.88844 | 0.14405 | 9.82762 | 7.767 | 0 |
| *Austroafricana parva* | 0 | 0 | 2.64487 | 0.79275 | 0.24187 | 0 | 0 |
| *Thanatephorus cucumeris* | 0 | 0.01701 | 4.14575 | 7.70401 | 6.44687 | 0 | 0 |
| *Exophiala moniliae* | 0 | 0 | 25.5535 | 7.08935 | 5.17399 | 0 | 0 |
| *Drechslera poae* | 17.4632 | 1.7687 | 0 | 25.0437 | 43.7672 | 0 | 0 |
| *Filobasidium chernovii* | 0.14198 | 26.0235 | 1.20343 | 0.14405 | 6.34431 | 0 | 0 |
| *Sporobolomyces roseus* | 0 | 0 | 26.6681 | 3.60135 | 8.14568 | 0 | 0 |
| *Aspergillus ochraceopetaliformis* | 0.10648 | 0 | 0.21689 | 0.72027 | 0.80055 | 0 | 0 |
| *Phellinus gilvus* | 0 | 0 | 15.4469 | 3.94096 | 6.64273 | 0 | 0 |
| *Trechispora nivea* | 0 | 0 | 3.00762 | 2.78416 | 2.86096 | 0 | 0 |
| *Psathyrella candolleana* | 0 | 0 | 5.0421 | 2.53957 | 5.78104 | 0 | 0 |
| *Torula hollandica* | 0 | 0 | 14.2992 | 10.8882 | 7.17753 | 6151.46 | 0 |
| *Psathyrella trinitatensis* | 0 | 0 | 0.97311 | 0.29754 | 0.54166 | 0 | 0 |
| *Mycopappus alni* | 0.10648 | 0 | 0.15931 | 0.28811 | 0.0488 | 7.767 | 0 |
| *Monilia mumeicola* | 1.31329 | 0.01701 | 1.09402 | 0.07248 | 0.33384 | 0 | 0 |
| *Naganishia diffluens* | 0 | 0 | 15.0554 | 5.99831 | 14.6889 | 0.04149 | 0 |
| *Pyrenochaetopsis pratorum* | 0 | 0 | 6.85201 | 1.5846 | 14.6272 | 0 | 0 |
| *Aspergillus clavatus* | 0 | 0 | 0.21689 | 0.0307 | 0 | 7.80849 | 0 |
| *Vishniacozyma heimaeyensis* | 0 | 0 | 6.27621 | 11.4523 | 8.94648 | 0 | 0 |
| *Curvibasidium cygneicollum* | 0 | 0 | 2.27833 | 2.97052 | 4.21046 | 0 | 0 |
| *Verrucaria elaeomelaena* | 0 | 0 | 12.3203 | 0.95158 | 15.0602 | 0 | 0 |
| *Neophaeococcomyces catenatus* | 0 | 0 | 22.9707 | 1.36851 | 2.89765 | 0 | 0 |
| *Neoascochyta paspali* | 0 | 0 | 33.3791 | 9.72366 | 11.5924 | 0 | 0 |
| *Holtermanniella wattica* | 0 | 0 | 5.73694 | 0.07203 | 22.7873 | 0 | 0 |
| *Exophiala xenobiotica* | 0 | 0 | 0 | 5.76217 | 33.901 | 36660.2 | 0 |
| *Symmetrospora gracilis* | 0 | 0 | 1.023 | 13.0262 | 0.0488 | 0 | 0 |
| *Meristemomyces frigidus* | 0 | 0 | 58.024 | 6.96804 | 3.97223 | 0 | 0 |
| *Pseudoteratosphaeria ohnowa* | 0 | 0 | 0.10173 | 1.42184 | 0.50076 | 0 | 0 |
| *Cupulina montana* | 0 | 2.09209 | 0.84622 | 0.64824 | 0 | 0 | 0 |
| *Ochrolechia yasudae* | 0 | 0 | 1.42606 | 0.07203 | 1.05032 | 0 | 0 |
| *Hypoxylon hypomiltum* | 0 | 0 | 1.79247 | 0 | 0.48373 | 0 | 0 |
| *Devriesia fraserae* | 0 | 0.17687 | 3.86557 | 0.0887 | 0.89326 | 0 | 0 |
| *Lanzia luteovirescens* | 0 | 0.03402 | 0.27447 | 0 | 0.16692 | 15.534 | 0 |
| *Cylindroseptoria pistaciae* | 0 | 0 | 1.72165 | 0.61392 | 1.38826 | 0 | 0 |
| *Gelidatrema spencermartinsiae* | 0 | 0 | 4.7811 | 2.28239 | 32.1324 | 0 | 0 |
| *Trechispora mollusca* | 0 | 0 | 1.57578 | 0.94336 | 0.0976 | 0 | 0 |
| *Schaereria cinereorufa* | 0.03549 | 0.17687 | 0.21689 | 0 | 0.16692 | 0 | 0 |
| *Alternaria botryospora* | 6.38896 | 0 | 44.1236 | 0 | 6.84507 | 0 | 0 |
| *Trichoderma atroviride* | 0 | 0.35374 | 0.15931 | 0.21608 | 0.0488 | 0 | 0 |
| *Exophiala cancerae* | 0 | 0 | 0.27447 | 2.37689 | 0.31681 | 0 | 0 |
| *Hemileucoglossum littorale* | 0 | 0 | 8.18063 | 0.27627 | 2.21096 | 0 | 0 |
| *Podosphaera xanthii* | 0 | 3.89502 | 10.6322 | 0 | 9.48997 | 0 | 0 |
| *Cladophialophora modesta* | 0 | 0 | 1.37617 | 0.21608 | 0.20439 | 0 | 0 |
| *Pyrenophora japonica* | 9.37047 | 2.82992 | 0 | 11.5243 | 39.5492 | 0 | 0 |
| *Alternaria oxytropis* | 0.10648 | 0 | 0.10173 | 0.21653 | 0 | 0 | 0 |
| *Staurosphaeria lycii* | 0 | 0 | 18.3283 | 0.10272 | 0.03747 | 0 | 0 |
| *Venturia viennotii* | 0.17747 | 0 | 0.15931 | 0.21608 | 0 | 7.767 | 0 |
| *Claviceps loudetiae* | 0 | 0.01701 | 0.70824 | 0.14405 | 0.16692 | 0 | 0 |
| *Hasegawazyma lactosa* | 0 | 0 | 0.21689 | 1.28027 | 0 | 0 | 0 |
| *Knufia tsunedae* | 0 | 0 | 5.28236 | 4.60973 | 3.17147 | 0 | 0 |
| *Naganishia globosa* | 0 | 0 | 14.967 | 0 | 16.3241 | 0 | 0 |
| *Lapidomyces hispanicus* | 0 | 0 | 40.4157 | 6.50759 | 1.87369 | 0 | 0 |
| *Candida hyderabadensis* | 0.03549 | 0 | 0.25315 | 0 | 18.1644 | 0 | 0 |
| *Phylliscum demangeonii* | 0 | 0 | 4.67487 | 7.77893 | 2.24843 | 0 | 0 |
| *Vermiconia calcicola* | 0 | 0 | 7.02475 | 1.68732 | 3.41617 | 0 | 0 |
| *Phaeotheca salicorniae* | 0 | 0 | 7.15531 | 4.82582 | 0.48716 | 0 | 0 |
| *Pseudofusicoccum violaceum* | 0.03549 | 0 | 0.23032 | 0.49355 | 0 | 0 | 0 |
| *Figure S4 Continued* |  |  |  |  |  |  |  |
|  | **Low** | | | | | **Medium** | **High** |
| **Fungal Species** | **50%** | **65%** | **70%** | **75%** | **80%** | **85%** | **95%** |
| *Hortaea thailandica* | 0 | 0 | 5.18801 | 14.3965 | 0.0488 | 3130.1 | 0 |
| *Didymella glomerata* | 0 | 0 | 6.56224 | 10.1297 | 4.49687 | 0 | 0 |
| *Peniophora nuda* | 0 | 0 | 22.3968 | 3.13101 | 5.24634 | 0 | 0 |
| *Tremella exigua* | 0 | 0 | 0.10173 | 2.07791 | 0.0488 | 0 | 0 |
| *Teratosphaeria zuluensis* | 0 | 0.01701 | 0.92128 | 0 | 1.2863 | 0 | 0 |
| *Phaeosaccardinula ficus* | 0 | 0 | 2.64487 | 0.10272 | 3.26023 | 0 | 0 |
| *Catenulostroma elginense* | 0 | 0 | 23.6127 | 0.35479 | 0 | 0 | 0 |
| *Ceratobasidium anceps* | 0 | 0 | 0 | 12.9054 | 1.98252 | 0 | 0 |
| *Kondoa miscanthi* | 0 | 0 | 0.8138 | 0.32145 | 0.712 | 0 | 0 |
| *Knufia aspidiotus* | 0 | 0 | 2.79395 | 0 | 10.131 | 0 | 0 |
| *Sarcinomyces crustaceus* | 0 | 0 | 0.11516 | 32.5687 | 5.21754 | 0 | 0 |
| *Cinereomyces lindbladii* | 0 | 0 | 97.9167 | 0 | 5.51467 | 0 | 0 |
| *Tremella indecorata* | 0 | 0 | 0.05758 | 0.49355 | 0.03747 | 0 | 0 |
| *Rhodonia placenta* | 17.0727 | 0 | 14.5851 | 0 | 4.00606 | 0 | 0 |
| *Teratosphaeria hortaea* | 0 | 0 | 6.51044 | 0.47101 | 0.43922 | 0 | 0 |
| *Mrakiella aquatica* | 0 | 0 | 0.10173 | 2.48639 | 7.3649 | 0 | 0 |
| *Umbilicaria lyngei* | 0 | 0.17687 | 2.03451 | 0 | 0.68672 | 0 | 0 |
| *Stereum sanguinolentum* | 0 | 0 | 7.78101 | 0 | 2.3469 | 0 | 0 |
| *Taphrina carpini* | 0 | 0 | 4.83569 | 3.43797 | 1.61138 | 0 | 0 |
| *Homortomyces tamaricis* | 0.07099 | 0 | 0 | 0.50419 | 0.83575 | 0 | 0 |
| *Epicoccum huancayense* | 0 | 0 | 0.10173 | 0.06139 | 0.0976 | 7.767 | 0 |
| *Mycosphaerella punctiformis* | 0 | 0 | 15.1571 | 0.3188 | 0.0976 | 0 | 0 |
| *Polycephalomyces cuboideus* | 0 | 0.53061 | 0.10173 | 0.0307 | 0.14641 | 0 | 0 |
| *Saxophila tyrrhenica* | 0 | 0.01701 | 0 | 0.07203 | 1.30634 | 0 | 0 |
| *Phoma crystallifera* | 0 | 0.03402 | 1.01726 | 0.06139 | 0.14641 | 0 | 0 |
| *Emarcea eucalyptigena* | 0 | 0.17687 | 0 | 0.22717 | 2.33687 | 0 | 0 |
| *Heterobasidion occidentale* | 0 | 0 | 13.8999 | 3.67338 | 0 | 7.767 | 0 |
| *Robillarda sessilis* | 0 | 0 | 0.67752 | 0.57622 | 0.0488 | 0 | 0 |
| *Pseudoseptoria collariana* | 0 | 0 | 0.15931 | 0.46044 | 0.11242 | 0 | 0 |
| *Sistotrema oblongisporum* | 0 | 0 | 20.4468 | 3.63544 | 0.8346 | 0 | 0 |
| *Sarea resinae* | 0.10648 | 0 | 0.37619 | 0 | 0.0488 | 0 | 0 |
| *Amauroderma calcigenum* | 0.14198 | 0 | 0.15931 | 0 | 0.53683 | 0 | 0 |
| *Peniophora lycii* | 0 | 0 | 0.15931 | 0.0307 | 0.07495 | 0 | 0 |
| *Hypholoma fasciculare* | 0 | 0 | 4.38379 | 0.07203 | 4.48982 | 0 | 0 |
| *Davidiellomyces australiensis* | 0 | 0 | 1.91741 | 0.14405 | 1.66919 | 0 | 0 |

**Figure S4.** Heatmap displaying abundant fungal species found in in >20% of all CA house 1 painted drywall samples from the moisture availability experiments where samples were incubated at 50%, 65%, 70%, 75%, 80%, 85%, and 95% continuously for 4 weeks. Darker colors indicate higher spore equivalent/cm^2^ drywall determined from qPCR

|  | **Low** | | | | | **Medium** | **High** |
| --- | --- | --- | --- | --- | --- | --- | --- |
| **Fungal Genus** | **50%** | **65%** | **70%** | **75%** | **80%** | **85%** | **95%** |
| *Penicillium* | *28.5018547* | *10.7386991* | *117.760276* | *45.3484175* | *31.394353* | *4580.712* | *8693886.41* |
| *Cladosporium* | *156.245535* | *98.9027717* | *1088.91074* | *453.755588* | *492.045911* | *11712.7143* | *3455964.26* |
| *Aspergillus* | *99.8452269* | *22.2315784* | *313.93489* | *30.921734* | *102.930945* | *177471.941* | *1260753.56* |
| *Mycosphaerella* | *84.2277723* | *36.4956536* | *496.624298* | *192.510761* | *260.394502* | *3821.40395* | *514208.267* |
| *Alternaria* | *170.01729* | *3.15675262* | *370.559655* | *164.01658* | *90.4709853* | *15471.8578* | *171990.502* |
| *Stemphylium* | *0* | *1.06121955* | *18.3336348* | *87.9292181* | *29.9474313* | *0* | *17017.0055* |
| *Toxicocladosporium* | *17.001729* | *3.89113834* | *53.7493485* | *51.0062341* | *41.9465052* | *0* | *16533.6821* |
| *Talaromyces* | *0* | *0* | *9.43551785* | *1.39921108* | *2.77296025* | *1871.84625* | *14859.6914* |
| *Wallemia* | *12.706929* | *0.67012628* | *437.304404* | *45.2664288* | *173.288128* | *4233.39166* | *13709.803* |
| *Didymella* | *36.4880531* | *14.8827348* | *101.793031* | *162.341367* | *42.6171291* | *6648.54933* | *11188.4857* |
| *Paradendryphiella* | *0* | *6.66746519* | *42.810839* | *25.9637964* | *23.6446718* | *0* | *7879.16787* |
| *Epicoccum* | *190.639429* | *132.288077* | *160.869235* | *1014.64414* | *635.395936* | *14919.9081* | *5497.96132* |
| *Rhodotorula* | *45.5390779* | *0* | *264.399147* | *105.056302* | *88.2104326* | *3200.00272* | *4297.72793* |
| *Ramularia* | *0* | *0* | *33.6127388* | *19.0427243* | *47.4879739* | *0.04148839* | *3246.54201* |
| *Candida* | *1.49075703* | *8.90497015* | *157.821771* | *71.3008786* | *76.5118661* | *2043.34251* | *2742.47029* |
| *Periconia* | *23.5681588* | *0* | *45.2265848* | *7.66871906* | *41.9494482* | *0* | *2535.16724* |
| *Vishniacozyma* | *43.3384366* | *30.5374288* | *560.050295* | *138.868121* | *172.50811* | *1809.71027* | *1369.9437* |
| *Aureobasidium* | *7.38279673* | *2.07485836* | *46.6463126* | *25.1273207* | *47.8658691* | *1.86697747* | *892.848855* |
| *Kondoa* | *0* | *0* | *50.4827022* | *31.1597563* | *14.4926284* | *0.04148839* | *621.693846* |
| *Curvularia* | *0* | *0.35373985* | *0.41245276* | *0.59627828* | *0.51202444* | *31.0679875* | *582.001932* |
| *Phaeosphaeria* | *0* | *1.76869925* | *7.25894327* | *9.84476177* | *9.41885929* | *0* | *521.880783* |
| *Hypoxylon* | *0.14197686* | *0.54761861* | *15.1621495* | *1.53263057* | *2.37924918* | *0* | *370.968071* |
| *Claviceps* | *0* | *9.93917363* | *260.493463* | *79.2100572* | *61.8921456* | *0* | *358.143994* |
| *Cystofilobasidium* | *0* | *2.12243909* | *153.868366* | *24.5429199* | *189.698754* | *2788.35188* | *310.846923* |
| *Torula* | *34.8198249* | *15.9182932* | *122.203066* | *55.6504864* | *22.8763758* | *14570.8862* | *207.231282* |
| *Filobasidium* | *78.5841921* | *30.9524225* | *180.55711* | *85.4205887* | *51.7597271* | *0* | *179.071997* |
| *Phoma* | *0.10648265* | *0.03401768* | *1.0172561* | *0.19481188* | *22.2924004* | *7.76699688* | *179.071997* |
| *Ganoderma* | *0* | *0* | *172.358187* | *70.1089456* | *84.9336948* | *0* | *120.242297* |
| *Austroafricana* | *0* | *0* | *3.06520245* | *1.05958967* | *0.37694579* | *0* | *103.615641* |
| *Venturia* | *10.5772761* | *0* | *30.4806484* | *16.4335893* | *3.88329528* | *613.592754* | *60.1211484* |
| *Symmetrospora* | *0* | *0* | *26.6767651* | *30.235378* | *18.9402969* | *0* | *60.1211484* |
| *Botrytis* | *223.542566* | *97.789721* | *466.298229* | *192.2008* | *203.651331* | *26159.3285* | *0* |
| *Podosphaera* | *97.0056897* | *69.1737408* | *2123.27722* | *678.360943* | *691.475541* | *1579.30547* | *0* |
| *Knufia* | *0* | *0.01700884* | *49.0992935* | *5.90063504* | *47.4378476* | *16.7786454* | *0* |
| *Peziza* | *0* | *3.59553667* | *1.52778862* | *8.11828403* | *2.05814475* | *2508.73999* | *0* |
| *Coniosporium* | *0.49691901* | *0* | *74.6818411* | *14.8834636* | *20.5200874* | *240.776903* | *0* |
| *Erysiphe* | *0.07098843* | *0.58163629* | *11.0155243* | *9.79957031* | *12.5441855* | *0* | *0* |
| *Trichoderma* | *0* | *3.44557277* | *6.91691241* | *4.64864938* | *1.48029411* | *0* | *0* |
| *Neodevriesia* | *0* | *5.53799019* | *79.0545058* | *11.9458153* | *16.7631435* | *0* | *0* |
| *Taphrina* | *42.7705291* | *3.71426842* | *40.3861546* | *25.4154291* | *152.173019* | *0* | *0* |
| *Umbilicaria* | *0.85186116* | *0.17686992* | *27.1875033* | *3.79714986* | *12.5501195* | *0* | *0* |
| *Blumeria* | *6.38895871* | *4.21819226* | *106.907052* | *61.7016479* | *115.837856* | *0* | *0* |
| *Coprinopsis* | *0* | *0.01700884* | *10.1993265* | *2.29520367* | *9.65428097* | *44.5170406* | *0* |
| *Cystotheca* | *0* | *4.47332486* | *153.53002* | *106.561052* | *97.8889921* | *0* | *0* |
| *Leptodontidium* | *48.0591672* | *0* | *88.6298457* | *59.3400508* | *7.71343652* | *2733.9829* | *0* |
| *Grammothele* | *0.03549422* | *0* | *0.93067174* | *0.44038143* | *0.3508004* | *0* | *0* |
| *Rhizocarpon* | *0.03549422* | *0* | *21.1627015* | *1.69441121* | *5.67581101* | *0* | *0* |
| *Verrucocladosporium* | *0* | *0* | *0.72551343* | *1.03281701* | *2.03757036* | *46.6019813* | *0* |
| *Trametes* | *0* | *0.01700884* | *78.6334507* | *54.389911* | *34.1377036* | *0.04148839* | *0* |
| *Exophiala* | *0* | *0* | *36.4507426* | *15.8967195* | *48.9328541* | *36660.2253* | *0* |

**Figure S5.** Heatmap displaying the 50 most abundant fungal genera found in the CA house 1 drywall A samples from the moisture availability experiments where samples were incubated at 50%, 65%, 70%, 75%, 80%, 85%, and 95% continuously for 4 weeks. Darker colors indicate higher spore equivalent/cm^2^ drywall determined from qPCR.

|  | **Relative Humidity Condition** | | | | | | |
| --- | --- | --- | --- | --- | --- | --- | --- |
| **Bacterial Species** | **50%** | **65%** | **70%** | **75%** | **80%** | **85%** | **95%** |
| *Brevundimonas diminuta* | 9.50E+03 | 8.98E+03 | 2.34E+03 | 4.70E+02 | 1.26E+03 | 6.32E+03 | 1.09E+05 |
| *Paenibacillus lautus* | 1.85E+02 | 1.34E+03 | 0.00E+00 | 5.41E+02 | 0.00E+00 | 8.00E+02 | 1.05E+05 |
| *Bosea genosp.* | 1.61E+04 | 1.37E+04 | 8.93E+03 | 4.43E+03 | 1.04E+04 | 3.02E+04 | 1.74E+04 |
| *Serratia marcescens* | 4.60E+05 | 5.32E+05 | 1.18E+05 | 1.60E+05 | 3.52E+05 | 4.98E+05 | 1.36E+04 |
| *Brachybacterium conglomeratum* | 4.62E+04 | 4.83E+04 | 5.34E+03 | 1.50E+04 | 3.24E+04 | 6.17E+04 | 1.15E+04 |
| *Kocuria palustris* | 1.00E+05 | 9.49E+04 | 2.32E+04 | 2.40E+04 | 6.49E+04 | 1.91E+05 | 1.14E+04 |
| *Pseudomonas stutzeri* | 6.00E+03 | 1.95E+03 | 1.36E+03 | 5.42E+02 | 7.94E+03 | 1.53E+04 | 9.49E+03 |
| *Cellulomonas xylanilytica* | 8.88E+04 | 6.91E+04 | 1.80E+04 | 1.47E+04 | 3.53E+04 | 9.02E+04 | 9.28E+03 |
| *Sphingomonas wittichii* | 8.95E+04 | 1.12E+05 | 3.29E+04 | 2.92E+04 | 9.47E+04 | 1.57E+05 | 6.12E+03 |
| *Brevibacillus reuszeri* | 5.18E+03 | 4.33E+03 | 1.64E+03 | 2.43E+03 | 6.58E+03 | 1.09E+04 | 5.93E+03 |
| *Bacillus firmus* | 2.85E+03 | 1.08E+03 | 7.23E+02 | 1.18E+03 | 9.43E+02 | 6.18E+02 | 5.57E+03 |
| *Roseomonas mucosa* | 3.41E+04 | 2.84E+04 | 4.72E+03 | 5.20E+03 | 1.00E+04 | 4.11E+04 | 5.44E+03 |
| *Methylobacterium adhaesivum* | 1.69E+05 | 2.08E+05 | 5.16E+04 | 4.44E+04 | 1.50E+05 | 2.56E+05 | 5.01E+03 |
| *Actinomadura vinacea* | 3.80E+03 | 2.81E+03 | 3.50E+02 | 6.36E+02 | 0.00E+00 | 7.59E+03 | 4.89E+03 |
| *Streptomyces aculeolatus* | 1.85E+02 | 3.13E+03 | 2.57E+02 | 8.71E+02 | 1.78E+03 | 4.51E+03 | 4.35E+03 |
| *Nitrosovibrio tenuis* | 2.37E+03 | 2.51E+03 | 9.38E+02 | 1.41E+03 | 9.72E+03 | 1.35E+04 | 4.25E+03 |
| *Caulobacter henricii* | 6.65E+03 | 6.70E+03 | 1.24E+03 | 3.63E+03 | 3.14E+03 | 8.97E+03 | 3.66E+03 |
| *Pseudoclavibacter bifida* | 1.72E+04 | 6.68E+03 | 4.10E+03 | 3.37E+03 | 5.12E+03 | 1.67E+04 | 3.21E+03 |
| *Rathayibacter caricis* | 1.79E+04 | 2.85E+04 | 8.05E+03 | 1.16E+04 | 2.77E+04 | 5.09E+04 | 2.83E+03 |
| *Microbispora rosea* | 9.23E+03 | 8.52E+03 | 1.50E+03 | 2.19E+03 | 3.14E+03 | 1.43E+04 | 2.77E+03 |
| *Bacillus muralis* | 1.62E+04 | 1.46E+04 | 4.57E+03 | 4.71E+03 | 9.09E+03 | 1.31E+04 | 2.61E+03 |
| *Paenibacillus barengoltzii* | 1.85E+02 | 0.00E+00 | 0.00E+00 | 9.47E+01 | 0.00E+00 | 1.24E+03 | 1.56E+03 |
| *Pseudomonas viridiflava* | 1.08E+05 | 1.18E+05 | 2.72E+04 | 2.63E+04 | 1.23E+05 | 2.08E+05 | 1.24E+03 |
| *Actinoallomurus iriomotensis* | 7.02E+03 | 2.90E+03 | 2.15E+03 | 2.00E+03 | 9.43E+02 | 1.68E+04 | 9.04E+02 |
| *Rhodococcus fascians* | 1.19E+04 | 1.43E+04 | 1.74E+03 | 6.61E+03 | 1.26E+03 | 3.07E+04 | 8.29E+02 |
| *Pseudomonas fragi* | 2.96E+04 | 3.20E+04 | 1.40E+04 | 1.03E+04 | 2.23E+04 | 4.53E+04 | 5.54E+02 |
| *Bacillus flexus* | 2.14E+04 | 1.57E+05 | 2.40E+03 | 3.34E+03 | 5.44E+03 | 2.11E+04 | 5.47E+02 |
| *Kocuria rhizophila* | 1.83E+04 | 2.81E+04 | 5.33E+03 | 9.61E+03 | 2.47E+04 | 1.04E+05 | 5.33E+02 |
| *Erwinia dispersa* | 2.90E+04 | 3.01E+04 | 4.12E+03 | 5.96E+03 | 2.21E+04 | 3.52E+04 | 5.28E+02 |
| *Sphingomonas azotifigens* | 1.24E+04 | 2.30E+04 | 6.87E+03 | 6.57E+03 | 2.34E+04 | 1.62E+04 | 5.20E+02 |
| *Sphingomonas echinoides* | 3.30E+04 | 2.96E+04 | 1.13E+04 | 1.09E+04 | 2.92E+04 | 3.66E+04 | 4.94E+02 |
| *Bacillus cereus* | 2.67E+05 | 1.00E+04 | 9.90E+03 | 3.36E+03 | 3.27E+04 | 1.56E+04 | 4.45E+02 |
| *Sphingomonas yabuuchiae* | 1.09E+04 | 1.02E+04 | 6.58E+03 | 4.69E+03 | 4.28E+03 | 2.32E+04 | 4.45E+02 |
| *Bacillus thermoamylovorans* | 1.91E+03 | 1.46E+03 | 7.46E+01 | 8.94E+02 | 3.55E+03 | 1.58E+03 | 4.13E+02 |
| *Paracoccus marcusii* | 1.08E+04 | 1.56E+04 | 3.80E+03 | 4.57E+03 | 1.84E+04 | 4.08E+04 | 4.09E+02 |
| *Lactobacillus zeae* | 1.38E+05 | 1.02E+05 | 3.14E+04 | 4.02E+04 | 4.63E+04 | 1.25E+05 | 3.01E+02 |
| *Agrococcus jenensis* | 1.43E+03 | 8.72E+02 | 1.82E+02 | 2.35E+02 | 9.43E+02 | 1.58E+03 | 2.59E+02 |
| *Bacillus humi* | 3.80E+03 | 1.69E+03 | 0.00E+00 | 2.12E+02 | 6.29E+02 | 8.00E+02 | 2.56E+02 |
| *Candidatus Nitrososphaera SCA1145* | 2.86E+04 | 2.69E+04 | 8.67E+03 | 7.60E+03 | 1.41E+04 | 3.04E+04 | 2.26E+02 |
| *Luteibacter rhizovicinus* | 6.08E+03 | 8.11E+03 | 6.63E+02 | 6.31E+03 | 2.82E+03 | 2.64E+04 | 2.10E+02 |
| *Acinetobacter johnsonii* | 1.78E+05 | 1.76E+05 | 1.01E+05 | 6.81E+04 | 1.61E+05 | 2.37E+05 | 2.02E+02 |
| *Lysinibacillus boronitolerans* | 6.23E+03 | 3.82E+03 | 5.32E+02 | 1.72E+03 | 4.08E+03 | 8.19E+03 | 1.93E+02 |
| *Bacillus coagulans* | 3.22E+03 | 4.93E+03 | 1.36E+03 | 2.92E+03 | 6.90E+03 | 6.18E+03 | 1.86E+02 |
| *Brevibacterium paucivorans* | 8.65E+04 | 4.07E+04 | 4.34E+03 | 8.36E+03 | 2.86E+04 | 3.88E+04 | 1.51E+02 |
| *Paracoccus aminovorans* | 2.49E+04 | 5.37E+04 | 8.57E+03 | 1.87E+04 | 2.46E+04 | 9.04E+04 | 1.47E+02 |
| *Staphylococcus epidermidis* | 9.23E+03 | 1.15E+04 | 2.94E+03 | 2.64E+03 | 8.79E+03 | 2.81E+04 | 1.11E+02 |
| *Bacillus foraminis* | 0.00E+00 | 1.62E+03 | 4.58E+02 | 1.74E+03 | 0.00E+00 | 0.00E+00 | 1.11E+02 |
| *Corynebacterium kroppenstedtii* | 1.27E+03 | 2.36E+03 | 1.37E+03 | 1.20E+03 | 5.02E+03 | 6.89E+03 | 1.08E+02 |
| *Candidatus Nitrososphaera SCA1170* | 3.20E+03 | 3.08E+03 | 1.95E+03 | 4.70E+02 | 0.00E+00 | 3.16E+03 | 8.41E+01 |
| *Staphylococcus aureus* | 6.52E+03 | 1.27E+04 | 3.14E+03 | 2.64E+03 | 1.59E+04 | 1.44E+04 | 7.54E+01 |
| *Staphylococcus sciuri* | 4.86E+03 | 1.40E+04 | 6.48E+03 | 3.65E+03 | 6.70E+03 | 2.07E+04 | 7.54E+01 |
| *Methylobacterium organophilum* | 2.44E+03 | 4.97E+03 | 6.48E+02 | 2.68E+03 | 6.29E+02 | 3.02E+03 | 7.54E+01 |
| *Pantoea ananatis* | 2.74E+03 | 1.48E+03 | 8.49E+02 | 2.12E+02 | 1.88E+03 | 4.58E+03 | 7.54E+01 |
| *Sphingobacterium faecium* | 3.78E+03 | 4.36E+03 | 1.79E+03 | 1.89E+02 | 6.29E+02 | 1.27E+04 | 7.54E+01 |
| *Bacillus selenatarsenatis* | 3.08E+03 | 4.21E+02 | 0.00E+00 | 2.35E+02 | 0.00E+00 | 0.00E+00 | 7.54E+01 |
| *Rhizobium leguminosarum* | 8.27E+02 | 2.54E+03 | 9.15E+02 | 1.27E+03 | 3.14E+02 | 6.16E+03 | 7.21E+01 |
| *Janthinobacterium lividum* | 3.53E+04 | 3.22E+04 | 8.18E+03 | 9.14E+03 | 3.88E+04 | 6.40E+04 | 4.21E+01 |
| *Salana multivorans* | 9.73E+03 | 1.16E+04 | 2.08E+03 | 1.93E+03 | 3.14E+02 | 3.82E+03 | 4.21E+01 |
| *Figure S6 Continued* |  |  |  |  |  |  |  |
|  | **Relative Humidity Conditions** | | | | | | |
| **Bacterial Species** | **50%** | **65%** | **70%** | **75%** | **80%** | **85%** | **95%** |
| *Mycobacterium celatum* | 2.14E+03 | 2.11E+02 | 1.49E+02 | 1.30E+03 | 1.26E+03 | 0.00E+00 | 4.21E+01 |
| *Agrobacterium vitis* | 7.14E+02 | 4.21E+02 | 1.82E+02 | 0.00E+00 | 0.00E+00 | 2.38E+03 | 4.21E+01 |
| *Rhodobacter sphaeroides* | 1.01E+03 | 6.61E+02 | 5.89E+02 | 9.47E+01 | 0.00E+00 | 6.18E+02 | 3.60E+01 |
| *Bifidobacterium adolescentis* | 4.78E+04 | 4.48E+04 | 7.63E+03 | 7.19E+03 | 2.03E+04 | 9.03E+04 | 0.00E+00 |
| *Streptococcus agalactiae* | 5.94E+03 | 6.25E+03 | 8.42E+03 | 2.97E+03 | 8.37E+03 | 1.10E+04 | 0.00E+00 |
| *Pedobacter cryoconitis* | 1.77E+04 | 1.62E+04 | 7.20E+03 | 4.38E+03 | 7.95E+03 | 1.43E+04 | 0.00E+00 |
| *Rothia dentocariosa* | 4.11E+04 | 3.33E+04 | 1.68E+03 | 1.24E+04 | 2.84E+04 | 7.08E+04 | 0.00E+00 |
| *[Ruminococcus] gnavus* | 3.23E+04 | 1.49E+04 | 2.45E+04 | 6.41E+03 | 1.57E+04 | 3.80E+04 | 0.00E+00 |
| *Haemophilus influenzae* | 3.74E+04 | 1.35E+05 | 8.99E+03 | 9.02E+03 | 1.57E+04 | 3.14E+04 | 0.00E+00 |
| *Acinetobacter lwoffii* | 1.98E+04 | 3.84E+04 | 2.61E+04 | 8.70E+03 | 2.53E+04 | 3.11E+04 | 0.00E+00 |
| *Rothia mucilaginosa* | 1.18E+05 | 1.41E+05 | 6.30E+04 | 3.07E+04 | 7.48E+04 | 1.99E+05 | 0.00E+00 |
| *Haemophilus parainfluenzae* | 2.97E+05 | 2.49E+05 | 8.23E+04 | 6.75E+04 | 1.50E+05 | 3.77E+05 | 0.00E+00 |
| *Veillonella parvula* | 4.71E+04 | 5.40E+04 | 1.63E+04 | 1.80E+04 | 3.68E+04 | 8.00E+04 | 0.00E+00 |
| *Nocardioides plantarum* | 1.51E+04 | 1.80E+04 | 4.68E+03 | 3.62E+03 | 7.32E+03 | 2.97E+04 | 0.00E+00 |
| *Faecalibacterium prausnitzii* | 2.00E+05 | 1.45E+05 | 4.16E+04 | 3.28E+04 | 9.60E+04 | 1.81E+05 | 0.00E+00 |
| *Acinetobacter rhizosphaerae* | 7.48E+03 | 1.70E+04 | 4.17E+03 | 3.04E+03 | 8.26E+03 | 1.39E+04 | 0.00E+00 |
| *Neisseria subflava* | 8.32E+04 | 1.07E+05 | 6.76E+04 | 1.44E+04 | 6.68E+04 | 1.40E+05 | 0.00E+00 |
| *Flavobacterium succinicans* | 6.38E+04 | 4.86E+04 | 1.74E+04 | 3.19E+04 | 2.75E+04 | 6.16E+04 | 0.00E+00 |
| *Rothia aeria* | 4.06E+04 | 1.49E+04 | 4.69E+03 | 6.92E+03 | 1.63E+04 | 3.44E+04 | 0.00E+00 |
| *Macrococcus caseolyticus* | 2.39E+03 | 9.44E+03 | 3.92E+03 | 4.08E+03 | 5.12E+03 | 8.03E+03 | 0.00E+00 |
| *Prevotella copri* | 1.03E+05 | 3.72E+04 | 5.35E+03 | 2.00E+04 | 8.47E+03 | 2.62E+04 | 0.00E+00 |
| *Veillonella dispar* | 1.24E+04 | 3.25E+04 | 9.17E+03 | 9.03E+03 | 1.02E+04 | 3.98E+04 | 0.00E+00 |
| *Corynebacterium durum* | 6.39E+04 | 3.69E+04 | 9.17E+03 | 1.01E+04 | 1.59E+04 | 6.21E+04 | 0.00E+00 |
| *Coprococcus eutactus* | 2.69E+04 | 1.97E+04 | 6.02E+03 | 9.94E+03 | 5.12E+03 | 2.66E+04 | 0.00E+00 |
| *Pasteurella multocida* | 5.96E+03 | 9.21E+03 | 1.24E+03 | 2.52E+03 | 5.01E+03 | 1.10E+04 | 0.00E+00 |
| *Bacteroides ovatus* | 1.24E+04 | 1.03E+04 | 4.83E+03 | 1.48E+03 | 4.18E+03 | 1.57E+04 | 0.00E+00 |
| *Blautia producta* | 1.76E+04 | 1.57E+04 | 7.99E+03 | 4.95E+03 | 1.49E+04 | 2.07E+04 | 0.00E+00 |
| *Peptostreptococcus anaerobius* | 2.90E+04 | 1.16E+04 | 9.07E+03 | 1.94E+03 | 6.68E+03 | 7.12E+03 | 0.00E+00 |
| *Streptococcus anginosus* | 1.54E+04 | 1.03E+04 | 2.35E+03 | 3.75E+03 | 4.08E+03 | 1.45E+04 | 0.00E+00 |
| *Deinococcus aquatilis* | 3.00E+04 | 1.49E+04 | 3.55E+03 | 7.60E+03 | 4.72E+03 | 2.31E+04 | 0.00E+00 |
| *Bacteroides fragilis* | 9.78E+03 | 1.09E+04 | 3.96E+03 | 4.02E+02 | 4.70E+03 | 9.61E+03 | 0.00E+00 |
| *Lactobacillus iners* | 2.16E+04 | 5.91E+03 | 1.64E+03 | 5.79E+03 | 4.08E+03 | 7.57E+03 | 0.00E+00 |
| *Prevotella melaninogenica* | 1.33E+04 | 1.86E+04 | 2.02E+03 | 4.92E+03 | 7.63E+03 | 9.13E+03 | 0.00E+00 |
| *Prevotella nanceiensis* | 1.09E+04 | 9.89E+03 | 4.03E+03 | 4.38E+03 | 1.14E+04 | 1.12E+04 | 0.00E+00 |
| *Psychrobacter sanguinis* | 4.53E+03 | 6.26E+03 | 2.52E+03 | 4.90E+03 | 9.43E+02 | 1.44E+04 | 0.00E+00 |
| *Aggregatibacter segnis* | 1.14E+04 | 8.75E+03 | 2.23E+03 | 1.89E+03 | 4.49E+03 | 3.18E+03 | 0.00E+00 |
| *Plesiomonas shigelloides* | 2.35E+03 | 9.34E+03 | 1.62E+03 | 1.46E+03 | 7.31E+03 | 8.49E+03 | 0.00E+00 |
| *Bacteroides uniformis* | 3.53E+04 | 2.58E+04 | 6.93E+03 | 5.47E+03 | 4.09E+03 | 2.95E+04 | 0.00E+00 |
| *Methylotenera mobilis* | 1.24E+04 | 8.38E+03 | 1.19E+03 | 2.10E+03 | 4.81E+03 | 1.97E+04 | 0.00E+00 |
| *Leuconostoc mesenteroides* | 3.11E+03 | 1.79E+03 | 1.31E+03 | 9.66E+02 | 0.00E+00 | 4.05E+03 | 0.00E+00 |
| *Edaphobacter modestum* | 2.44E+03 | 8.21E+03 | 1.36E+03 | 1.06E+03 | 2.92E+03 | 2.22E+03 | 0.00E+00 |
| *Kushneria aurantia* | 3.78E+03 | 4.35E+03 | 1.49E+02 | 2.26E+03 | 3.35E+03 | 2.38E+03 | 0.00E+00 |
| *Asticcacaulis biprosthecium* | 7.13E+03 | 8.62E+03 | 1.03E+03 | 2.35E+02 | 3.87E+03 | 1.11E+04 | 0.00E+00 |
| *Bifidobacterium longum* | 3.57E+03 | 4.78E+03 | 1.19E+03 | 1.81E+03 | 2.09E+03 | 1.44E+04 | 0.00E+00 |
| *Geodermatophilus obscurus* | 2.55E+03 | 2.03E+03 | 1.49E+02 | 1.03E+03 | 2.82E+03 | 1.42E+03 | 0.00E+00 |
| *Alloiococcus otitis* | 4.69E+03 | 7.72E+03 | 8.12E+02 | 1.20E+03 | 2.51E+03 | 0.00E+00 | 0.00E+00 |
| *[Eubacterium] biforme* | 5.62E+03 | 2.42E+03 | 7.46E+01 | 3.06E+03 | 0.00E+00 | 5.98E+03 | 0.00E+00 |
| *Ruminococcus bromii* | 3.75E+03 | 1.50E+03 | 6.30E+02 | 3.30E+02 | 2.09E+03 | 2.65E+03 | 0.00E+00 |
| *Sphingobacterium multivorum* | 3.22E+03 | 9.38E+03 | 2.60E+03 | 8.49E+02 | 2.52E+03 | 6.04E+03 | 0.00E+00 |
| *Prosthecobacter debontii* | 4.05E+03 | 1.83E+03 | 7.46E+01 | 4.96E+02 | 6.29E+02 | 3.78E+03 | 0.00E+00 |
| *Porphyromonas endodontalis* | 2.85E+03 | 6.22E+03 | 1.87E+03 | 2.24E+03 | 3.55E+03 | 3.20E+03 | 0.00E+00 |
| *Actinomyces europaeus* | 5.06E+03 | 2.81E+03 | 3.65E+02 | 7.99E+02 | 0.00E+00 | 1.07E+04 | 0.00E+00 |
| *Clostridium hiranonis* | 2.62E+03 | 5.06E+03 | 3.01E+03 | 3.52E+02 | 0.00E+00 | 2.40E+03 | 0.00E+00 |
| *Lactobacillus ruminis* | 2.44E+03 | 2.09E+03 | 4.39E+02 | 3.79E+02 | 3.14E+02 | 6.18E+02 | 0.00E+00 |
| *Wolbachia Wolbachia endosymbiont* | 3.23E+03 | 2.94E+03 | 1.24E+03 | 1.20E+03 | 3.14E+02 | 4.95E+03 | 0.00E+00 |
| *Sphingopyxis alaskensis* | 5.52E+03 | 2.38E+03 | 3.73E+02 | 1.51E+03 | 0.00E+00 | 2.20E+03 | 0.00E+00 |
| *Deinococcus alpinitundrae* | 1.80E+03 | 1.46E+03 | 9.00E+02 | 6.59E+02 | 9.43E+02 | 0.00E+00 | 0.00E+00 |
| *Bacteroides caccae* | 5.92E+03 | 5.27E+03 | 1.18E+03 | 0.00E+00 | 9.43E+02 | 5.01E+03 | 0.00E+00 |
| *Pseudomonas citronellolis* | 2.08E+04 | 9.90E+02 | 4.48E+02 | 3.98E+03 | 4.08E+03 | 1.60E+03 | 0.00E+00 |
| *Figure S6 Continued* |  |  |  |  |  |  |  |
|  | **Relative Humidity Conditions** | | | | | | |
| **Bacterial Species** | **50%** | **65%** | **70%** | **75%** | **80%** | **85%** | **95%** |
| *Bacteroides coprophilus* | 5.39E+03 | 2.90E+03 | 1.49E+02 | 1.18E+03 | 5.64E+03 | 8.00E+02 | 0.00E+00 |
| *Anoxybacillus kestanbolensis* | 2.55E+03 | 2.45E+03 | 4.24E+02 | 2.12E+02 | 0.00E+00 | 0.00E+00 | 0.00E+00 |
| *Capnocytophaga ochracea* | 8.27E+02 | 2.45E+03 | 3.65E+02 | 5.19E+02 | 2.92E+03 | 5.98E+03 | 0.00E+00 |
| *Actinobacillus porcinus* | 1.65E+03 | 1.59E+03 | 4.06E+02 | 4.74E+02 | 9.43E+02 | 4.42E+03 | 0.00E+00 |
| *Jeotgalicoccus psychrophilus* | 1.43E+03 | 9.90E+02 | 7.89E+02 | 6.36E+02 | 2.19E+03 | 6.18E+02 | 0.00E+00 |
| *Virgisporangium ochraceum* | 1.84E+03 | 1.34E+03 | 3.73E+02 | 1.89E+02 | 3.14E+02 | 9.13E+03 | 0.00E+00 |
| *Collinsella stercoris* | 2.33E+03 | 1.37E+03 | 7.46E+01 | 3.30E+02 | 3.14E+02 | 0.00E+00 | 0.00E+00 |
| *Collinsella aerofaciens* | 8.27E+02 | 1.58E+03 | 0.00E+00 | 1.89E+02 | 0.00E+00 | 1.02E+04 | 0.00E+00 |
| *Burkholderia andropogonis* | 1.85E+02 | 2.37E+03 | 7.46E+01 | 5.87E+02 | 3.14E+02 | 3.71E+03 | 0.00E+00 |
| *Brevibacterium aureum* | 1.01E+03 | 9.63E+02 | 1.49E+02 | 0.00E+00 | 0.00E+00 | 3.80E+03 | 0.00E+00 |
| *[Eubacterium] cylindroides* | 4.99E+03 | 2.99E+03 | 0.00E+00 | 2.00E+03 | 0.00E+00 | 1.42E+04 | 0.00E+00 |
| *Staphylococcus equorum* | 3.70E+02 | 2.19E+03 | 2.57E+02 | 0.00E+00 | 3.14E+02 | 3.18E+03 | 0.00E+00 |
| *Roseburia faecis* | 8.98E+02 | 9.63E+02 | 0.00E+00 | 0.00E+00 | 2.19E+03 | 2.20E+03 | 0.00E+00 |
| *Ochrobactrum intermedium* | 8.27E+02 | 8.26E+02 | 7.46E+01 | 2.12E+02 | 1.46E+03 | 8.00E+02 | 0.00E+00 |
| *Pseudoxanthomonas mexicana* | 1.43E+03 | 2.49E+03 | 2.57E+02 | 0.00E+00 | 0.00E+00 | 4.27E+04 | 0.00E+00 |
| *Akkermansia muciniphila* | 3.70E+02 | 5.29E+03 | 0.00E+00 | 1.89E+02 | 3.14E+02 | 1.17E+04 | 0.00E+00 |
| *Selenomonas noxia* | 8.98E+02 | 2.57E+03 | 0.00E+00 | 0.00E+00 | 6.29E+02 | 2.20E+03 | 0.00E+00 |
| *Clostridium perfringens* | 3.70E+02 | 0.00E+00 | 2.61E+03 | 1.60E+03 | 0.00E+00 | 4.74E+03 | 0.00E+00 |
| *Chlamydomonas reinhardtii* | 1.08E+03 | 8.72E+02 | 1.82E+02 | 0.00E+00 | 6.29E+02 | 3.02E+03 | 0.00E+00 |
| *Bdellovibrio bacteriovorus* | 1.80E+03 | 6.32E+02 | 9.12E+02 | 1.89E+02 | 6.29E+02 | 1.24E+03 | 0.00E+00 |
| *Parabacteroides distasonis* | 2.37E+03 | 2.11E+02 | 2.98E+02 | 1.08E+03 | 1.46E+03 | 8.00E+02 | 0.00E+00 |
| *Sphingobacterium mizutaii* | 1.11E+03 | 1.08E+03 | 5.14E+02 | 3.52E+02 | 6.29E+02 | 0.00E+00 | 0.00E+00 |
| *Psychrobacter pacificensis* | 8.98E+02 | 1.62E+03 | 2.75E+02 | 1.17E+02 | 6.29E+02 | 0.00E+00 | 0.00E+00 |
| *Marinilactibacillus psychrotolerans* | 2.37E+03 | 1.70E+03 | 0.00E+00 | 2.35E+02 | 0.00E+00 | 3.16E+03 | 0.00E+00 |
| *Enterococcus cecorum* | 7.14E+02 | 6.61E+02 | 7.00E+02 | 0.00E+00 | 3.14E+02 | 1.58E+03 | 0.00E+00 |
| *Leptolyngbya frigida* | 0.00E+00 | 5.62E+03 | 0.00E+00 | 2.12E+02 | 4.38E+03 | 3.16E+03 | 0.00E+00 |
| *Pseudonocardia halophobica* | 7.14E+02 | 1.98E+03 | 7.46E+01 | 0.00E+00 | 3.14E+02 | 3.16E+03 | 0.00E+00 |
| *Conchiformibius kuhniae* | 8.98E+02 | 2.11E+02 | 0.00E+00 | 2.28E+03 | 0.00E+00 | 1.58E+03 | 0.00E+00 |
| *Vibrio rumoiensis* | 2.93E+03 | 4.21E+02 | 0.00E+00 | 7.31E+02 | 3.14E+02 | 0.00E+00 | 0.00E+00 |
| *Acinetobacter schindleri* | 7.14E+02 | 1.16E+03 | 2.75E+02 | 6.36E+02 | 0.00E+00 | 0.00E+00 | 0.00E+00 |
| *Corynebacterium variabile* | 1.43E+03 | 9.16E+02 | 0.00E+00 | 0.00E+00 | 1.78E+03 | 1.24E+03 | 0.00E+00 |
| *Pseudomonas veronii* | 1.84E+03 | 2.38E+03 | 2.75E+02 | 0.00E+00 | 0.00E+00 | 1.24E+03 | 0.00E+00 |
| *Phormidium animale* | 0.00E+00 | 1.53E+03 | 1.49E+02 | 0.00E+00 | 2.92E+03 | 1.58E+03 | 0.00E+00 |
| *Sphingomonas changbaiensis* | 1.54E+03 | 4.95E+02 | 2.98E+02 | 1.89E+02 | 0.00E+00 | 0.00E+00 | 0.00E+00 |
| *Trabulsiella farmeri* | 0.00E+00 | 7.05E+02 | 7.46E+01 | 1.08E+03 | 0.00E+00 | 3.16E+03 | 0.00E+00 |
| *Lactococcus garvieae* | 1.43E+03 | 5.61E+03 | 0.00E+00 | 5.41E+02 | 0.00E+00 | 1.24E+03 | 0.00E+00 |
| *Streptococcus minor* | 8.98E+02 | 1.04E+03 | 0.00E+00 | 0.00E+00 | 0.00E+00 | 0.00E+00 | 0.00E+00 |
| *Variovorax paradoxus* | 7.14E+02 | 4.21E+02 | 0.00E+00 | 9.47E+01 | 0.00E+00 | 2.20E+03 | 0.00E+00 |
| *Pseudomonas umsongensis* | 1.85E+02 | 5.41E+02 | 0.00E+00 | 3.52E+02 | 6.29E+02 | 0.00E+00 | 0.00E+00 |

**Figure S6.** Heatmap displaying abundant bacterial species found in >20% of the CA dust embedded in carpet A samples from the moisture availability experiments where samples were incubated at 50%, 65%, 70%, 75%, 80%, 85%, and 95% ERH continuously for 4 weeks. Darker colors indicate higher cells/mg filter dust determined from qPCR.

|  | **Relative Humidity Conditions** | | | | | | |
| --- | --- | --- | --- | --- | --- | --- | --- |
| **Bacterial Species** | **50%** | **65%** | **70%** | **75%** | **80%** | **85%** | **95%** |
| *Rothia mucilaginosa* | 20.01511 | 2.241393 | 77.63747 | 387.8113 | 42.585 | 9.784687 | 44.95933 |
| *Methylobacterium adhaesivum* | 62.90463 | 17.93114 | 204.4427 | 59.80441 | 47.29096 | 34.3147 | 42.15339 |
| *Rothia dentocariosa* | 125.8093 | 22.7875 | 12.99857 | 31.64032 | 4.572061 | 0 | 33.23893 |
| *Veillonella parvula* | 0 | 22.41393 | 38.03757 | 2.095106 | 20.18507 | 9.55738 | 31.37586 |
| *Paracoccus aminovorans* | 11.4372 | 16.43688 | 93.58192 | 31.96576 | 16.35757 | 13.1414 | 24.55933 |
| *Lactobacillus zeae* | 0 | 0 | 0 | 2.400528 | 3.443771 | 0 | 21.85255 |
| *Cellulomonas xylanilytica* | 0 | 4.10922 | 26.24052 | 8.250222 | 30.26424 | 0.238934 | 18.94745 |
| *Serratia marcescens* | 0 | 28.76454 | 27.85478 | 0.300066 | 4.226098 | 0.238934 | 17.89609 |
| *Kocuria palustris* | 38.60057 | 8.218441 | 393.0498 | 81.36621 | 105.3212 | 19.20254 | 16.29025 |
| *Pseudomonas viridiflava* | 40.03022 | 0 | 23.55121 | 2.700594 | 21.29576 | 0.238934 | 15.70379 |
| *Staphylococcus epidermidis* | 0 | 0 | 3.177538 | 0.29471 | 0.658795 | 0 | 15.26608 |
| *Rathayibacter caricis* | 0 | 0 | 8.54778 | 3.707146 | 1.641097 | 5.256559 | 14.93686 |
| *Brevibacterium paucivorans* | 0 | 12.70123 | 636.4706 | 3.268591 | 2.647866 | 0 | 14.88728 |
| *Veillonella dispar* | 0 | 17.93114 | 8.366699 | 2.389816 | 5.76151 | 0.955738 | 12.27967 |
| *Roseomonas mucosa* | 24.30406 | 5.977048 | 81.11909 | 11.08688 | 11.86082 | 12.15078 | 10.92628 |
| *Kocuria rhizophila* | 20.01511 | 0.747131 | 9.801893 | 8.521086 | 23.52462 | 2.389345 | 10.92628 |
| *Acinetobacter lwoffii* | 0 | 0 | 9.884736 | 9.310841 | 0.047416 | 9.796314 | 10.92628 |
| *Pseudoclavibacter bifida* | 0 | 0 | 62.96662 | 0.531775 | 5.317894 | 0.477869 | 9.473724 |
| *Sphingomonas echinoides* | 0 | 0 | 39.58478 | 21.0995 | 3.068427 | 0 | 6.816527 |
| *Haemophilus parainfluenzae* | 204.44 | 42.96003 | 48.96891 | 17.79035 | 132.855 | 18.59038 | 4.060167 |
| *Sphingomonas wittichii* | 1.429651 | 6.724179 | 8.906929 | 1.219263 | 11.12481 | 181.9121 | 4.060167 |
| *Bifidobacterium adolescentis* | 55.75637 | 0.373565 | 8.762721 | 8.380424 | 2.005519 | 2.628279 | 2.706778 |
| *Peptostreptococcus anaerobius* | 0 | 0 | 141.9527 | 1.200264 | 4.928336 | 0 | 2.706778 |
| *Microbispora rosea* | 0 | 0.373565 | 5.704899 | 3.282235 | 2.603283 | 2.115529 | 1.353389 |
| *Neisseria subflava* | 0 | 0.747131 | 15.0286 | 1.639327 | 22.62254 | 0 | 1.353389 |
| *Streptococcus agalactiae* | 0 | 2.241393 | 129.9075 | 0.300066 | 1.238888 | 0 | 1.353389 |
| *Bacillus cereus* | 0 | 0 | 0.701502 | 2.100462 | 0.437233 | 0 | 1.353389 |
| *Paracoccus marcusii* | 2.859301 | 0 | 50.46673 | 23.0338 | 33.51395 | 5.571646 | 0 |
| *Brachybacterium conglomeratum* | 0 | 10.45983 | 13.83451 | 22.98316 | 7.251683 | 1.194672 | 0 |
| *Acinetobacter johnsonii* | 0 | 1.867827 | 43.79145 | 9.727973 | 24.87558 | 0 | 0 |
| *Faecalibacterium prausnitzii* | 14.29651 | 10.45983 | 74.00249 | 8.401847 | 13.15952 | 6.376229 | 0 |
| *Coprococcus eutactus* | 0 | 0 | 34.33928 | 2.689882 | 15.11659 | 0 | 0 |
| *Rothia aeria* | 2.859301 | 3.362089 | 4.910515 | 5.970961 | 0.351419 | 0 | 0 |
| *Corynebacterium durum* | 50.03777 | 10.45983 | 7.716523 | 0.884131 | 4.174379 | 0 | 0 |
| *Rhodococcus fascians* | 0 | 29.51167 | 15.43305 | 0.300066 | 4.033424 | 7.406969 | 0 |
| *Haemophilus influenzae* | 1.429651 | 0 | 14.53601 | 1.494974 | 7.564361 | 0 | 0 |
| *Corynebacterium variabile* | 1.429651 | 0 | 1.588769 | 0 | 0.478148 | 0.466242 | 0 |
| *Bifidobacterium longum* | 7.148253 | 0 | 3.786157 | 2.389816 | 0.053918 | 0 | 0 |
| *Lysinibacillus boronitolerans* | 0 | 0.747131 | 2.898891 | 0 | 0.155252 | 0 | 0 |
| *Ruminococcus bromii* | 0 | 0.373565 | 3.971922 | 0.600132 | 0.142249 | 0.238934 | 0 |
| *Pseudomonas fragi* | 0 | 0.373565 | 0.794384 | 0.29471 | 0.161754 | 0.227307 | 0 |
| *Prevotella nanceiensis* | 0 | 0 | 7.716523 | 4.147366 | 8.237452 | 0 | 0 |
| *Bacteroides uniformis* | 0 | 0 | 11.54959 | 4.801055 | 5.929154 | 41.09673 | 0 |
| *Staphylococcus aureus* | 0 | 0 | 15.58221 | 0 | 0.376814 | 0 | 0 |
| *Sphingomonas azotifigens* | 2.859301 | 0 | 0 | 0.58942 | 0.598987 | 0 | 0 |
| *Bosea genosp.* | 0 | 0 | 6.355747 | 0.600132 | 0.868576 | 0 | 0 |
| *Ochrobactrum intermedium* | 0 | 1.494262 | 1.466711 | 12.7626 | 0.616411 | 0 | 0 |
| *Streptococcus minor* | 0 | 0.747131 | 0.701502 | 0 | 3.312551 | 0 | 0 |
| *Bacillus muralis* | 0 | 30.63237 | 9.82103 | 0 | 0.974331 | 0 | 0 |
| *Methylobacterium organophilum* | 0 | 0.373565 | 1.403004 | 2.700594 | 0.658795 | 0 | 0 |
| *Staphylococcus sciuri* | 0 | 1.494262 | 0 | 0.300066 | 0.856184 | 0 | 0 |
| *Aggregatibacter segnis* | 1.429651 | 0 | 0.701502 | 0 | 0.471646 | 0 | 0 |
| *Pseudomonas stutzeri* | 0 | 0 | 0 | 3.053458 | 0.053918 | 0.955738 | 0 |

**Figure S7**. Heatmap displaying abundant bacterial species found in >20% of the CA house 1 drywall A samples from the moisture availability experiments where samples were incubated at 50%, 65%, 70%, 75%, 80%, 85%, and 95% ERH continuously for 4 weeks. Darker colors indicate higher cells/mg filter dust determined from qPCR.

**
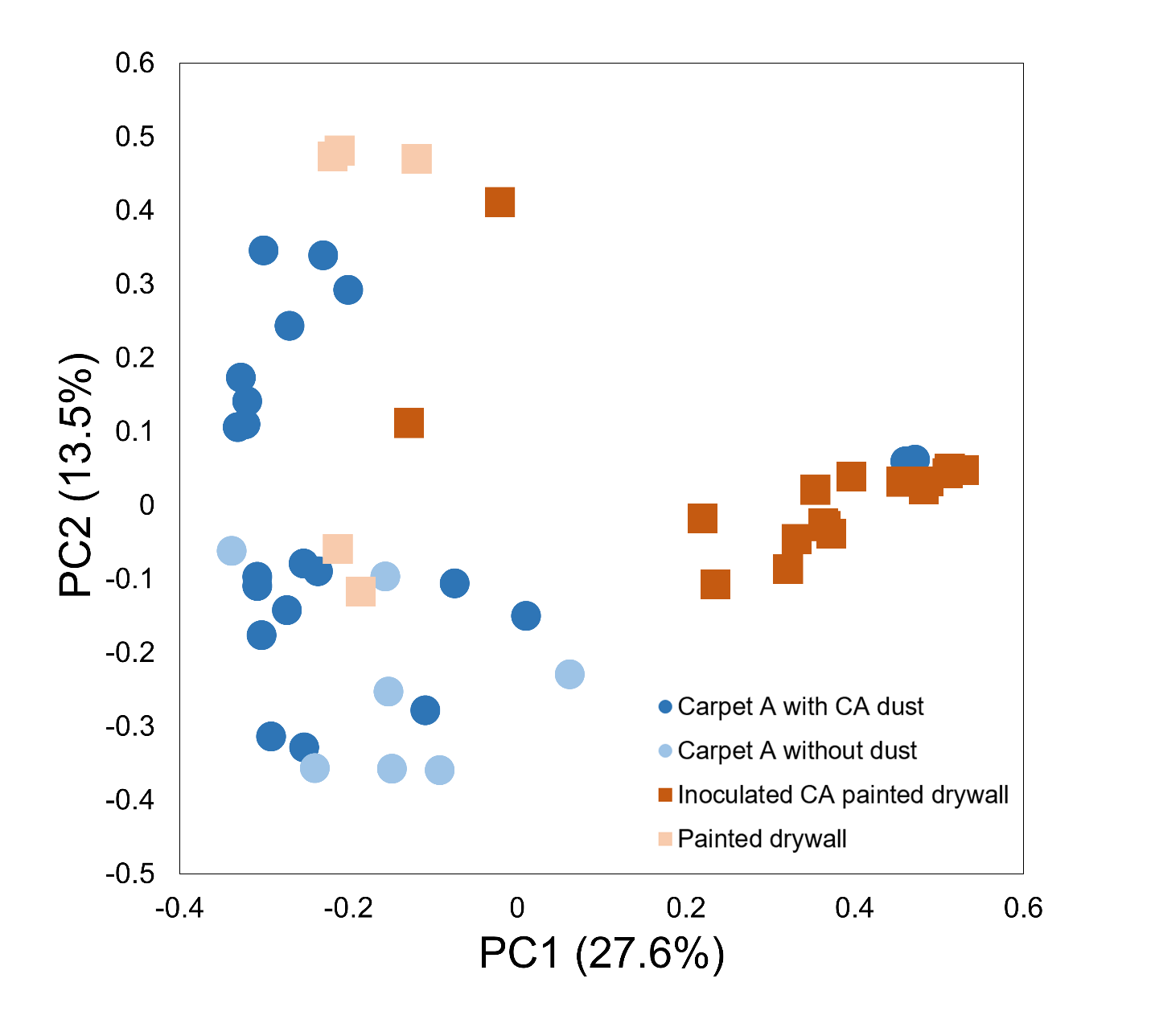
**

**Figure S8.** Bray-Curtis principal coordinate analysis (PCoA) displaying CA dust embedded in carpet A with dust, carpet A without dust, CA painted drywall A and painted drywall A from the moisture availability experiments. The results indicate the fungal species composition varied between the different sample groupings (R^2^ = 0.26, p=0.001).


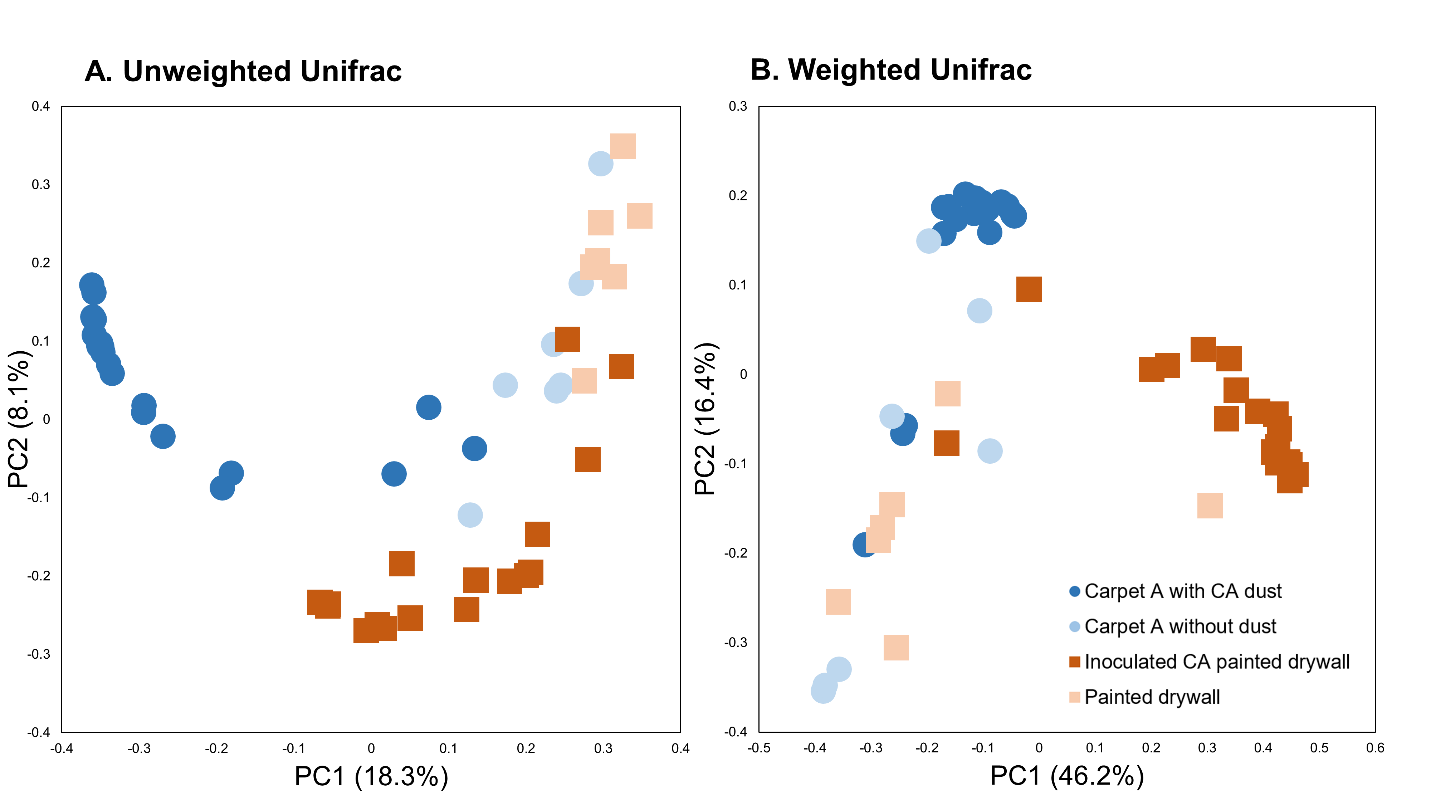


**Figure S9.** Unweighted Unifrac (A) and Weighted Unifrac (B) principal coordinate analysis (PCoA) of all the moisture availability samples. The results indicate the bacterial species composition varied between samples of carpet with dust (SFDCA), carpet without dust (CAS) inoculated painted drywall (SFAPDA), and painted drywall (PDAS) for both the Unweighted (R^2^ = 0.23, p=0.001) and Weighted (R^2^ = 0.48, p=0.001).

| **Abundant fungal species from Gainesville, FL dust and carpet samples** | | | | | | | | |
| --- | --- | --- | --- | --- | --- | --- | --- | --- |
|  | **Sterile Dust** | | **Dust** | | **Dust+CarpetA** | | **Dust+CarpetB** | |
| **Fungal species** | **50%** | **85%** | **50%** | **85%** | **50%** | **85%** | **50%** | **85%** |
| *Aspergillus sydowii* | 2.911968 | 0.896422 | 9.967157 | 20256.08 | 0.50677 | 184.7047 | 2.487039 | 3127.151 |
| *Aspergillus penicillioides* | 4.439274 | 1.053138 | 9.949315 | 1951.395 | 1.860633 | 622.6576 | 0.485869 | 1518.459 |
| *Penicillium chrysogenum* | 0.094863 | 0.017856 | 0 | 75.65812 | 0.053429 | 1.551609 | 0.801428 | 1285.166 |
| *Aspergillus hongkongensis* | 1.212518 | 0.1607 | 2.611678 | 1358.773 | 0.69491 | 22.15009 | 0.23711 | 1095.094 |
| *Aspergillus austroafricanus* | 0.804442 | 0.096906 | 3.589524 | 77.84713 | 0.104916 | 6.083527 | 0.054367 | 566.4877 |
| *Aspergillus ruber* | 2.415959 | 1.509415 | 1.769892 | 402.3043 | 0.540448 | 586.8417 | 1.447298 | 559.8784 |
| *Penicillium gladioli* | 0 | 0 | 0 | 3.67862 | 0 | 0.003724 | 0.214033 | 138.7354 |
| *Xenodidymella humicola* | 41.7748 | 29.65673 | 160.6054 | 13.67162 | 54.98534 | 10.95736 | 44.85153 | 73.18347 |
| *Aspergillus restrictus* | 0.257204 | 0.043339 | 0.692567 | 44.19509 | 0.104916 | 25.87827 | 0.23345 | 49.62623 |
| *Aspergillus gracilis* | 2.561143 | 0.334057 | 6.07451 | 32.50029 | 0.910566 | 1.045828 | 0.552222 | 45.29116 |
| *Candida parapsilosis* | 36.37126 | 48.70934 | 83.12314 | 86.465 | 21.36116 | 26.70287 | 13.64325 | 32.46548 |
| *Penicillium citrinum* | 4.806348 | 48.97942 | 17.0871 | 6.039359 | 6.291751 | 1.31399 | 1.520524 | 28.50651 |
| *Epicoccum nigrum* | 52.75107 | 30.07207 | 124.9156 | 45.35243 | 17.57861 | 22.95682 | 12.80584 | 23.72333 |
| *Curvularia eragrostidis* | 27.8248 | 13.42039 | 85.63533 | 7.248349 | 19.67829 | 6.709985 | 12.89996 | 23.20963 |
| *Pestalotiopsis coffeae-arabicae* | 16.11232 | 7.592308 | 35.14357 | 3.953645 | 5.079688 | 2.151248 | 4.772837 | 15.02712 |
| *Eupenidiella venezuelensis* | 29.0627 | 15.52488 | 89.06248 | 7.35724 | 14.97935 | 12.62666 | 6.387989 | 14.03682 |
| *Parengyodontium album* | 180.0791 | 37.23987 | 488.9284 | 19.4932 | 55.6566 | 49.86085 | 23.0557 | 13.82039 |
| *Sterigmatomyces halophilus* | 41.63121 | 29.87191 | 121.2228 | 14.88061 | 16.29371 | 15.45204 | 23.16453 | 13.757 |
| *Neodevriesia lagerstroemiae* | 40.34628 | 13.16623 | 122.0233 | 18.00918 | 30.40461 | 20.1672 | 18.35714 | 13.69141 |
| *Humicola olivacea* | 5.527063 | 5.121969 | 8.432805 | 0 | 2.84632 | 3.743826 | 0.664393 | 12.55248 |
| *Curvularia caricae-papayae* | 9.666558 | 5.420684 | 29.97374 | 4.612586 | 4.596551 | 0.398518 | 3.765031 | 11.71303 |
| *Aspergillus nidulans* | 0 | 0 | 0 | 72.41506 | 0 | 0 | 0.050596 | 11.30424 |
| *Coniosporium apollinis* | 40.95974 | 19.57809 | 76.33353 | 10.37692 | 18.69353 | 26.87053 | 4.841845 | 11.04408 |
| *Neodevriesia strelitziae* | 17.00887 | 7.091645 | 54.99156 | 10.37692 | 15.7996 | 7.377413 | 7.296055 | 10.78612 |
| *Curvularia lunata* | 17.62998 | 8.005076 | 63.38778 | 6.589409 | 12.56924 | 4.410506 | 7.142171 | 9.902941 |
| *Pyrenochaetopsis leptospora* | 13.61121 | 11.05351 | 61.861 | 11.96983 | 5.507117 | 7.643332 | 3.820069 | 8.418602 |
| *Devriesia strelitziicola* | 6.795592 | 8.131618 | 23.36525 | 3.294704 | 8.990108 | 5.203071 | 2.208441 | 8.095057 |
| *Pyrenochaeta nobilis* | 7.351058 | 4.255891 | 20.83989 | 0 | 8.645903 | 1.633547 | 3.844597 | 7.856761 |
| *Penicillium herquei* | 0.077706 | 0.150472 | 0.076952 | 3.67862 | 0.069297 | 0.014898 | 0.050596 | 6.739696 |
| *Hamigera insecticola* | 0.031923 | 0 | 0 | 5.380418 | 0.069297 | 0.011173 | 0 | 6.566991 |
| *Epicoccum brasiliense* | 4.550627 | 2.18445 | 18.09215 | 1.317882 | 4.722483 | 4.46935 | 2.073527 | 6.5014 |
| *Curvularia spicifera* | 4.565968 | 1.166684 | 12.51972 | 0 | 2.034846 | 0.986237 | 2.647486 | 6.41615 |
| *Aspergillus versicolor* | 0.090657 | 0.017856 | 0 | 47.65033 | 0 | 0.011173 | 0.050596 | 6.287173 |
| *Nigrospora oryzae* | 87.86462 | 9.261577 | 151.9642 | 11.14475 | 23.53359 | 5.100281 | 9.031504 | 6.221582 |
| *Zopfiella karachiensis* | 0.557466 | 1.590357 | 8.541654 | 0 | 0.2257 | 1.540435 | 1.206193 | 5.898037 |
| *Cladosporium adianticola* | 6.657762 | 1.783831 | 11.45177 | 1.976823 | 2.85282 | 1.258123 | 1.038538 | 5.425856 |
| *Wallemia tropicalis* | 34.86599 | 14.04841 | 38.08648 | 152.9203 | 6.133657 | 26.83323 | 16.37312 | 5.382129 |
| *Cladosporium halotolerans* | 7.232776 | 2.654092 | 12.42493 | 5.271527 | 4.351438 | 0.770965 | 2.551295 | 5.296879 |
| *Cladosporium delicatulum* | 36.05697 | 12.30728 | 68.17664 | 8.675122 | 22.45337 | 7.031038 | 15.78372 | 5.231288 |
| *Arxiella dolichandrae* | 2.691319 | 3.510111 | 9.229536 | 0.658941 | 11.47931 | 0.055867 | 1.178563 | 5.058584 |
| *Penicillium concentricum* | 0 | 0.02167 | 0 | 1.701798 | 0 | 0 | 0.007767 | 5.017062 |
| *Basidiobolus ranarum* | 2.577392 | 2.162273 | 16.66665 | 2.635763 | 2.89941 | 7.661207 | 2.692101 | 4.929607 |
| *Penicillium expansum* | 0.012951 | 0 | 0 | 4.062536 | 0 | 0.018622 | 0.003883 | 4.586403 |
| *Curvularia sorghina* | 2.818588 | 1.555354 | 12.14434 | 1.317882 | 0.700734 | 0.122907 | 0.976404 | 4.542676 |
| *Plectosphaerella oratosquillae* | 45.13675 | 0.622175 | 3.123128 | 0 | 0.471151 | 0.007449 | 0.350174 | 4.542676 |
| *Aspergillus unguis* | 0.314121 | 0.104534 | 0.89621 | 14.33056 | 0.192022 | 9.915993 | 0.948997 | 4.435562 |
| *Myxospora crassiseta* | 2.591492 | 0.373582 | 3.15034 | 0 | 12.82963 | 0.204846 | 0.680708 | 4.435562 |
| *Myrothecium gramineum* | 11.30201 | 4.248263 | 30.98348 | 6.80719 | 9.162969 | 5.345348 | 4.855393 | 4.240994 |
| *Microascus niger* | 2.127165 | 1.735124 | 8.455332 | 0 | 0.352308 | 0.859605 | 1.206082 | 4.155745 |
| *Exophiala xenobiotica* | 0.630058 | 1.0287 | 16.46769 | 0 | 5.256518 | 0.100561 | 0.416192 | 3.961176 |

**Figure S10.** Heatmap of the top 50 most abundant fungal species found in >20% of the Gainesville, FL carpet and dust samples from the collection location sample experiments where samples were incubated at 50% or 85% ERH for four weeks.

| **Abundant fungal species from San Francisco, CA dust and carpet samples** | | | | | | | | |
| --- | --- | --- | --- | --- | --- | --- | --- | --- |
|  | **Sterile Dust** | | **Dust** | | **Dust+CarpetA** | | **Dust+CarpetB** | |
| **Fungal species** | **50%** | **85%** | **50%** | **85%** | **50%** | **85%** | **50%** | **85%** |
| *Penicillium chrysogenum* | 4.99E+03 | 7.45E+01 | 2.84E+05 | 5.51E+04 | 1.89E+05 | 6.81E+04 | 2.00E+05 | 2.45E+08 |
| *Penicillium gladioli* | 0.00E+00 | 1.05E+01 | 1.46E+04 | 4.51E+03 | 1.42E+04 | 6.60E+03 | 1.17E+04 | 2.24E+07 |
| *Wallemia tropicalis* | 4.44E+03 | 1.90E+02 | 1.24E+07 | 3.78E+07 | 2.61E+06 | 3.61E+07 | 8.82E+06 | 4.46E+06 |
| *Wallemia canadensis* | 4.87E+04 | 6.45E+02 | 2.04E+07 | 3.67E+07 | 3.06E+06 | 4.12E+07 | 9.42E+06 | 1.35E+06 |
| *Wallemia muriae* | 7.43E+03 | 1.19E+03 | 2.18E+07 | 3.96E+07 | 3.62E+06 | 5.28E+07 | 1.03E+07 | 1.32E+06 |
| *Fusarium acutatum* | 7.56E+04 | 1.25E+04 | 2.05E+03 | 0.00E+00 | 3.99E+03 | 1.99E+03 | 0.00E+00 | 1.04E+06 |
| *Penicillium concentricum* | 0.00E+00 | 0.00E+00 | 0.00E+00 | 0.00E+00 | 2.17E+03 | 0.00E+00 | 5.86E+03 | 1.03E+06 |
| *Disculoides calophyllae* | 8.42E+03 | 5.74E+02 | 1.31E+04 | 0.00E+00 | 6.50E+03 | 0.00E+00 | 0.00E+00 | 7.75E+05 |
| *Wallemia sebi* | 1.76E+04 | 3.60E+02 | 1.30E+06 | 1.53E+06 | 3.28E+05 | 3.73E+06 | 5.40E+05 | 5.39E+05 |
| *Penicillium kongii* | 6.80E+03 | 4.26E+02 | 3.67E+04 | 2.71E+04 | 3.64E+04 | 1.65E+04 | 5.86E+03 | 5.35E+05 |
| *Aspergillus hongkongensis* | 1.03E+04 | 2.56E+02 | 4.97E+04 | 7.30E+03 | 8.98E+04 | 3.31E+04 | 0.00E+00 | 4.69E+05 |
| *Didymella calidophila* | 7.34E+03 | 9.03E+02 | 0.00E+00 | 0.00E+00 | 0.00E+00 | 0.00E+00 | 0.00E+00 | 4.14E+05 |
| *Fusarium culmorum* | 7.97E+04 | 6.70E+03 | 4.53E+03 | 3.65E+03 | 1.99E+04 | 0.00E+00 | 0.00E+00 | 2.81E+05 |
| *Aspergillus ruber* | 8.24E+03 | 2.11E+01 | 8.33E+06 | 2.61E+06 | 8.00E+06 | 2.37E+06 | 3.04E+05 | 2.54E+05 |
| *Humicola nigrescens* | 5.34E+03 | 2.75E+02 | 1.53E+03 | 0.00E+00 | 8.66E+03 | 0.00E+00 | 0.00E+00 | 2.40E+05 |
| *Gibberella intricans* | 4.77E+04 | 2.56E+03 | 3.51E+03 | 0.00E+00 | 4.33E+03 | 0.00E+00 | 0.00E+00 | 2.01E+05 |
| *Penicillium citrinum* | 2.35E+03 | 1.38E+02 | 3.51E+04 | 0.00E+00 | 3.21E+04 | 6.60E+03 | 0.00E+00 | 1.34E+05 |
| *Cryptococcus uniguttulatus* | 6.87E+04 | 1.81E+03 | 1.62E+04 | 0.00E+00 | 0.00E+00 | 0.00E+00 | 2.74E+03 | 1.34E+05 |
| *Debaryomyces hansenii* | 1.63E+04 | 1.28E+02 | 4.09E+03 | 0.00E+00 | 1.59E+04 | 0.00E+00 | 7.69E+03 | 1.20E+05 |
| *Naganishia albida* | 3.74E+05 | 1.24E+04 | 3.83E+04 | 4.51E+03 | 4.00E+04 | 0.00E+00 | 2.27E+04 | 6.76E+04 |
| *Penicillium expansum* | 3.53E+03 | 1.48E+02 | 7.02E+03 | 0.00E+00 | 8.87E+03 | 5.29E+03 | 0.00E+00 | 6.68E+04 |
| *Naganishia antarctica* | 1.08E+05 | 3.81E+03 | 1.02E+04 | 0.00E+00 | 3.51E+04 | 0.00E+00 | 5.48E+03 | 5.42E+04 |
| *Naganishia randhawae* | 8.60E+04 | 2.59E+03 | 4.60E+03 | 0.00E+00 | 2.55E+03 | 6.60E+03 | 1.37E+03 | 5.34E+04 |
| *Cladosporium sphaerospermum* | 4.20E+04 | 1.14E+03 | 1.66E+04 | 0.00E+00 | 2.89E+04 | 9.90E+03 | 9.13E+02 | 4.06E+04 |
| *Coniochaeta polymorpha* | 1.79E+04 | 4.64E+02 | 3.51E+03 | 0.00E+00 | 1.30E+04 | 3.30E+03 | 0.00E+00 | 4.03E+04 |
| *Cystobasidium slooffiae* | 4.58E+05 | 4.16E+03 | 1.07E+04 | 0.00E+00 | 3.99E+03 | 1.52E+04 | 1.74E+04 | 4.01E+04 |
| *Acremonium tubakii* | 6.08E+04 | 7.07E+03 | 1.51E+04 | 0.00E+00 | 1.89E+04 | 0.00E+00 | 0.00E+00 | 4.01E+04 |
| *Fusarium solani* | 6.97E+03 | 2.97E+02 | 1.02E+03 | 0.00E+00 | 0.00E+00 | 0.00E+00 | 0.00E+00 | 4.01E+04 |
| *Didymella gardeniae* | 7.22E+05 | 6.90E+04 | 1.59E+05 | 0.00E+00 | 8.59E+05 | 1.66E+04 | 7.69E+03 | 3.32E+04 |
| *Candida parapsilosis* | 1.65E+06 | 4.02E+04 | 4.23E+05 | 5.03E+05 | 1.86E+05 | 7.88E+05 | 2.30E+05 | 2.97E+04 |
| *Gibberella baccata* | 1.36E+05 | 1.05E+04 | 8.05E+03 | 0.00E+00 | 3.16E+04 | 6.60E+03 | 4.57E+02 | 2.75E+04 |
| *Verticillium dahliae* | 2.61E+04 | 1.55E+03 | 0.00E+00 | 0.00E+00 | 4.71E+03 | 0.00E+00 | 0.00E+00 | 2.75E+04 |
| *Penicillium brevicompactum* | 4.54E+02 | 4.21E+01 | 0.00E+00 | 0.00E+00 | 2.72E+03 | 3.30E+03 | 0.00E+00 | 2.67E+04 |
| *Rhodotorula diobovata* | 5.60E+04 | 1.76E+03 | 9.07E+03 | 0.00E+00 | 1.05E+04 | 0.00E+00 | 6.77E+03 | 2.67E+04 |
| *Exophiala dermatitidis* | 0.00E+00 | 3.16E+01 | 2.11E+04 | 0.00E+00 | 4.80E+04 | 0.00E+00 | 0.00E+00 | 2.67E+04 |
| *Endophoma elongata* | 4.54E+03 | 1.56E+03 | 0.00E+00 | 0.00E+00 | 0.00E+00 | 0.00E+00 | 0.00E+00 | 2.67E+04 |
| *Epicoccum nigrum* | 2.17E+06 | 3.24E+05 | 1.67E+05 | 1.54E+04 | 2.59E+05 | 6.42E+04 | 8.91E+04 | 1.66E+04 |
| *Coniosporium apollinis* | 1.77E+06 | 7.41E+04 | 1.44E+05 | 9.02E+03 | 1.71E+05 | 3.04E+04 | 1.45E+04 | 1.64E+04 |
| *Coniochaeta decumbens* | 1.32E+04 | 1.30E+03 | 0.00E+00 | 0.00E+00 | 1.73E+04 | 2.92E+04 | 0.00E+00 | 1.36E+04 |
| *Aspergillus niger* | 3.53E+03 | 1.05E+01 | 3.51E+03 | 1.08E+04 | 1.99E+03 | 5.98E+03 | 0.00E+00 | 1.35E+04 |
| *Didymella glomerata* | 1.16E+05 | 5.99E+03 | 1.18E+04 | 0.00E+00 | 1.92E+04 | 3.99E+03 | 3.65E+03 | 1.34E+04 |
| *Rhodotorula dairenensis* | 1.30E+04 | 7.98E+02 | 2.56E+03 | 0.00E+00 | 7.22E+02 | 0.00E+00 | 1.37E+03 | 1.34E+04 |
| *Alternaria nepalensis* | 1.22E+04 | 6.91E+02 | 1.02E+03 | 0.00E+00 | 2.89E+03 | 0.00E+00 | 0.00E+00 | 1.34E+04 |
| *Candida hyderabadensis* | 1.08E+05 | 3.34E+03 | 0.00E+00 | 0.00E+00 | 6.50E+03 | 0.00E+00 | 1.37E+03 | 1.34E+04 |
| *Fusarium proliferatum* | 4.71E+03 | 1.16E+02 | 0.00E+00 | 0.00E+00 | 7.22E+02 | 0.00E+00 | 0.00E+00 | 1.34E+04 |
| *Cladosporium delicatulum* | 3.30E+06 | 1.60E+05 | 3.29E+05 | 0.00E+00 | 2.90E+05 | 3.44E+04 | 3.30E+04 | 8.76E+03 |
| *Vishniacozyma victoriae* | 2.45E+06 | 1.03E+05 | 1.89E+05 | 0.00E+00 | 1.20E+05 | 2.32E+04 | 9.92E+04 | 8.76E+03 |
| *Mycosphaerella tassiana* | 2.43E+06 | 1.32E+05 | 2.24E+05 | 1.08E+04 | 2.43E+05 | 3.58E+04 | 6.85E+03 | 7.73E+03 |
| *Pseudopithomyces chartarum* | 5.71E+05 | 6.24E+04 | 4.78E+04 | 1.35E+04 | 6.14E+04 | 2.06E+04 | 1.35E+04 | 3.99E+03 |
| *Nigrospora oryzae* | 2.18E+06 | 4.58E+04 | 3.37E+05 | 1.08E+04 | 2.26E+05 | 1.39E+04 | 4.92E+04 | 3.86E+03 |

**Figure S11.** Heatmap of the top 50 most abundant fungal species found in >20% of the San Francisco, CA carpet and dust from the collection location sample experiments where samples were incubated at 50% or 85% ERH for four weeks.

| **Abundant fungal species from Columbus, OH dust and carpet samples** | | | | | | | | |
| --- | --- | --- | --- | --- | --- | --- | --- | --- |
|  | **Sterile Dust** | | **Dust** | | **Dust+CarpetA** | | **Dust+CarpetB** | |
| **Fungal species** | **50%** | **85%** | **50%** | **85%** | **50%** | **85%** | **50%** | **85%** |
| *Epicoccum nigrum* | 8.73E+04 | 3.87E+05 | 5.31E+05 | 6.27E+05 | 8.10E+05 | 5.97E+05 | 8.59E+04 | 1.72E+04 |
| *Xenodidymella humicola* | 3.28E+04 | 2.99E+05 | 3.81E+05 | 3.36E+05 | 6.31E+05 | 2.94E+05 | 4.16E+04 | 8.49E+03 |
| *Phaeotheca triangularis* | 2.20E+04 | 7.85E+04 | 1.74E+05 | 1.43E+05 | 3.02E+05 | 1.29E+05 | 3.80E+04 | 4.16E+03 |
| *Alternaria alternata* | 1.94E+04 | 7.94E+04 | 1.72E+05 | 7.59E+04 | 1.51E+05 | 1.04E+05 | 3.46E+04 | 2.60E+03 |
| *Nigrospora oryzae* | 3.17E+04 | 2.25E+05 | 3.24E+05 | 2.30E+05 | 1.41E+05 | 2.03E+05 | 2.49E+04 | 3.63E+03 |
| *Caloplaca ferrarii* | 2.79E+03 | 2.11E+04 | 4.37E+04 | 7.20E+04 | 6.58E+04 | 7.55E+03 | 2.39E+04 | 2.34E+03 |
| *Knufia marmoricola* | 1.71E+04 | 1.78E+05 | 1.68E+05 | 1.22E+05 | 3.07E+05 | 1.11E+05 | 2.14E+04 | 5.65E+03 |
| *Pseudopithomyces chartarum* | 2.01E+04 | 9.39E+04 | 1.23E+05 | 9.03E+04 | 2.89E+05 | 1.01E+05 | 2.13E+04 | 3.33E+03 |
| *Laetisaria arvalis* | 1.04E+03 | 8.41E+02 | 2.45E+04 | 4.22E+03 | 3.58E+03 | 2.67E+03 | 1.65E+04 | 2.09E+01 |
| *Neurospora terricola* | 1.13E+03 | 2.18E+03 | 1.03E+04 | 3.96E+03 | 3.46E+03 | 7.97E+03 | 1.50E+04 | 8.12E+01 |
| *Penicillium chrysogenum* | 4.74E+01 | 3.82E+02 | 2.53E+02 | 2.25E+02 | 0.00E+00 | 3.00E+03 | 1.31E+04 | 1.55E+03 |
| *Vermiconia calcicola* | 1.05E+04 | 5.27E+04 | 5.59E+04 | 8.84E+04 | 1.32E+05 | 6.85E+04 | 1.28E+04 | 1.73E+03 |
| *Mycoarthris corallina* | 9.21E+03 | 5.33E+04 | 6.44E+04 | 8.43E+04 | 1.15E+05 | 5.28E+04 | 1.26E+04 | 1.66E+03 |
| *Cladosporium delicatulum* | 8.34E+03 | 4.04E+04 | 4.94E+04 | 3.76E+04 | 2.13E+05 | 5.70E+04 | 1.24E+04 | 2.34E+03 |
| *Phaeophyscia imbricata* | 6.90E+03 | 6.09E+04 | 6.24E+04 | 5.81E+04 | 4.02E+04 | 6.00E+04 | 1.08E+04 | 1.71E+03 |
| *Candida parapsilosis* | 6.91E+03 | 3.52E+04 | 4.99E+04 | 3.93E+04 | 1.22E+05 | 5.59E+04 | 9.77E+03 | 1.18E+03 |
| *Coniosporium apollinis* | 1.07E+04 | 3.41E+04 | 7.70E+04 | 3.23E+04 | 7.67E+04 | 5.42E+04 | 9.34E+03 | 1.01E+03 |
| *Preussia flanaganii* | 9.00E+03 | 1.71E+04 | 3.78E+04 | 2.67E+04 | 1.10E+05 | 2.85E+04 | 8.53E+03 | 2.21E+03 |
| *Fusarium culmorum* | 7.74E+03 | 3.52E+04 | 3.87E+04 | 3.04E+04 | 6.69E+04 | 4.80E+04 | 7.61E+03 | 1.35E+03 |
| *Phoma bulgarica* | 3.97E+03 | 3.72E+04 | 3.02E+04 | 2.95E+04 | 7.97E+04 | 2.13E+04 | 7.57E+03 | 4.89E+02 |
| *Neodevriesia lagerstroemiae* | 3.20E+03 | 3.40E+04 | 2.79E+04 | 2.59E+04 | 5.55E+04 | 2.13E+04 | 7.17E+03 | 6.29E+02 |
| *Gibberella baccata* | 1.28E+03 | 1.02E+04 | 6.95E+03 | 9.99E+03 | 4.73E+04 | 1.89E+04 | 6.88E+03 | 3.66E+02 |
| *Helicodendron luteoalbum* | 6.88E+03 | 2.77E+04 | 1.66E+04 | 5.27E+04 | 8.84E+04 | 2.65E+04 | 6.06E+03 | 2.18E+02 |
| *Neoascochyta desmazieri* | 6.43E+03 | 2.67E+04 | 3.12E+04 | 3.14E+04 | 4.18E+04 | 1.07E+05 | 5.31E+03 | 6.00E+02 |
| *Symmetrospora foliicola* | 4.18E+03 | 2.45E+04 | 3.52E+04 | 1.55E+04 | 7.33E+04 | 2.35E+04 | 5.09E+03 | 5.78E+02 |
| *Gibberella intricans* | 4.70E+03 | 2.36E+04 | 2.25E+04 | 2.40E+04 | 6.17E+04 | 3.34E+04 | 4.73E+03 | 6.86E+02 |
| *Wallemia tropicalis* | 1.36E+03 | 3.66E+05 | 1.35E+04 | 1.01E+04 | 4.13E+03 | 1.52E+05 | 4.16E+03 | 1.85E+03 |
| *Epicoccum brasiliense* | 3.07E+03 | 1.05E+04 | 2.00E+04 | 4.02E+04 | 9.39E+04 | 1.61E+05 | 4.08E+03 | 1.10E+03 |
| *Plectosphaerella oratosquillae* | 2.69E+03 | 9.53E+03 | 1.07E+04 | 7.77E+03 | 1.46E+04 | 9.16E+03 | 3.90E+03 | 2.10E+02 |
| *Aspergillus hongkongensis* | 3.84E+03 | 1.54E+04 | 1.69E+04 | 1.05E+04 | 2.23E+04 | 1.98E+05 | 3.77E+03 | 8.34E+02 |
| *Pyrenochaetopsis leptospora* | 1.06E+04 | 2.26E+04 | 1.90E+04 | 3.74E+04 | 3.81E+04 | 4.48E+04 | 3.55E+03 | 2.28E+03 |
| *Monodictys castaneae* | 3.57E+03 | 4.61E+03 | 2.68E+04 | 1.25E+04 | 2.41E+04 | 1.78E+04 | 3.31E+03 | 3.15E+02 |
| *Cystofilobasidium capitatum* | 2.29E+03 | 2.15E+04 | 2.79E+04 | 1.45E+04 | 2.68E+04 | 9.35E+03 | 3.28E+03 | 2.64E+02 |
| *Phaeophyscia constipata* | 1.63E+03 | 1.66E+04 | 1.69E+04 | 1.52E+04 | 2.98E+04 | 1.61E+04 | 3.09E+03 | 4.44E+02 |
| *Exserohilum turcicum* | 1.01E+03 | 1.10E+04 | 1.84E+04 | 7.40E+03 | 1.85E+04 | 6.84E+03 | 2.89E+03 | 3.99E+02 |
| *Saitozyma flava* | 3.40E+02 | 3.32E+03 | 9.40E+03 | 1.07E+04 | 7.10E+01 | 1.52E+04 | 2.76E+03 | 2.04E+02 |
| *Articulospora proliferata* | 1.80E+03 | 3.74E+03 | 8.74E+03 | 5.29E+03 | 3.89E+03 | 8.58E+03 | 2.66E+03 | 4.40E+02 |
| *Cyberlindnera jadinii* | 3.18E+03 | 4.14E+04 | 1.48E+04 | 2.99E+04 | 7.25E+04 | 1.27E+04 | 2.61E+03 | 6.76E+02 |
| *Cystofilobasidium macerans* | 6.63E+02 | 2.58E+03 | 6.56E+03 | 2.91E+03 | 7.45E+03 | 2.30E+03 | 2.20E+03 | 5.01E+01 |
| *Phaeomycocentrospora cantuariensis* | 1.08E+03 | 3.44E+04 | 4.27E+03 | 2.05E+04 | 3.09E+04 | 4.84E+04 | 2.13E+03 | 8.12E+02 |
| *Trichosporon lactis* | 2.04E+03 | 1.44E+04 | 1.50E+04 | 7.22E+03 | 5.21E+04 | 1.75E+04 | 1.96E+03 | 2.30E+02 |
| *Cystofilobasidium infirmominiatum* | 5.29E+02 | 8.65E+03 | 1.33E+04 | 1.18E+04 | 2.00E+04 | 1.15E+04 | 1.89E+03 | 1.04E+02 |
| *Candida hyderabadensis* | 1.47E+03 | 1.56E+04 | 1.70E+04 | 5.53E+03 | 6.36E+04 | 5.18E+03 | 1.85E+03 | 1.87E+02 |
| *Naganishia diffluens* | 4.61E+02 | 7.14E+03 | 3.59E+03 | 5.90E+03 | 8.30E+05 | 2.00E+03 | 1.84E+03 | 1.51E+02 |
| *Naganishia albida* | 1.12E+03 | 4.43E+03 | 4.35E+03 | 4.49E+03 | 1.96E+04 | 5.69E+03 | 1.79E+03 | 5.67E+02 |
| *Collophora paarla* | 9.68E+02 | 4.13E+03 | 2.15E+04 | 2.34E+03 | 3.77E+04 | 5.18E+03 | 1.78E+03 | 7.63E+01 |
| *Pyrenochaeta keratinophila* | 2.79E+03 | 1.27E+04 | 1.20E+04 | 1.02E+04 | 1.34E+04 | 1.03E+04 | 1.74E+03 | 1.49E+03 |
| *Vishniacozyma carnescens* | 1.64E+03 | 1.00E+04 | 1.16E+04 | 9.69E+03 | 1.81E+04 | 8.05E+03 | 1.74E+03 | 2.87E+02 |
| *Verticillium dahliae* | 1.11E+03 | 9.20E+03 | 7.42E+03 | 8.79E+03 | 3.37E+04 | 1.21E+04 | 1.70E+03 | 1.67E+02 |
| *Candida tropicalis* | 1.77E+03 | 1.45E+04 | 8.00E+03 | 1.52E+04 | 1.42E+04 | 4.10E+04 | 1.51E+03 | 3.23E+02 |

**Figure S12.** Heatmap of the top 50 most abundant fungal species found in >20% of the Columbus, OH carpet and dust from the collection location sample experiments where samples were incubated at 50% or 85% ERH for four weeks.

**Figure S13.** Bray-Curtis principal coordinate analysis (PCoA) of the carpet and dust used in the collection location experiment where samples were incubated at 50% or 85% ERH for weeks continuously. Samples of dust were collected from San Francisco, CA (A), Columbus, OH (B), and Gainesville, FL (C). Statistical differences between sample type or incubation ERH level did not occur in the San Francisco, CA (A) and Columbus, OH samples (B), R^2^ = 0.05, p=0.86 and R^2^ = 0.08, p=0.25 respectively, however there was a clear distinction in the Gainesville, FL (C) samples of samples incubated at 50% ERH compared to 85% ERH, R2 = 0.22, p=0.021.


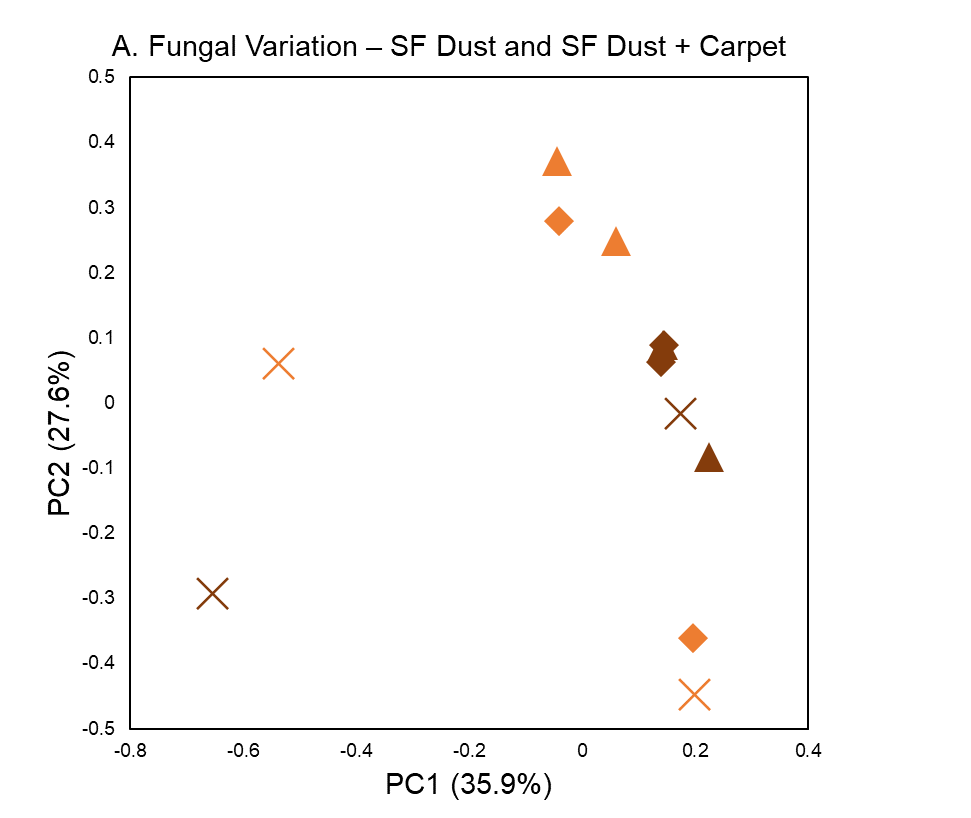

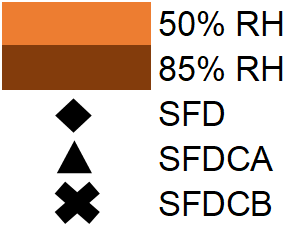

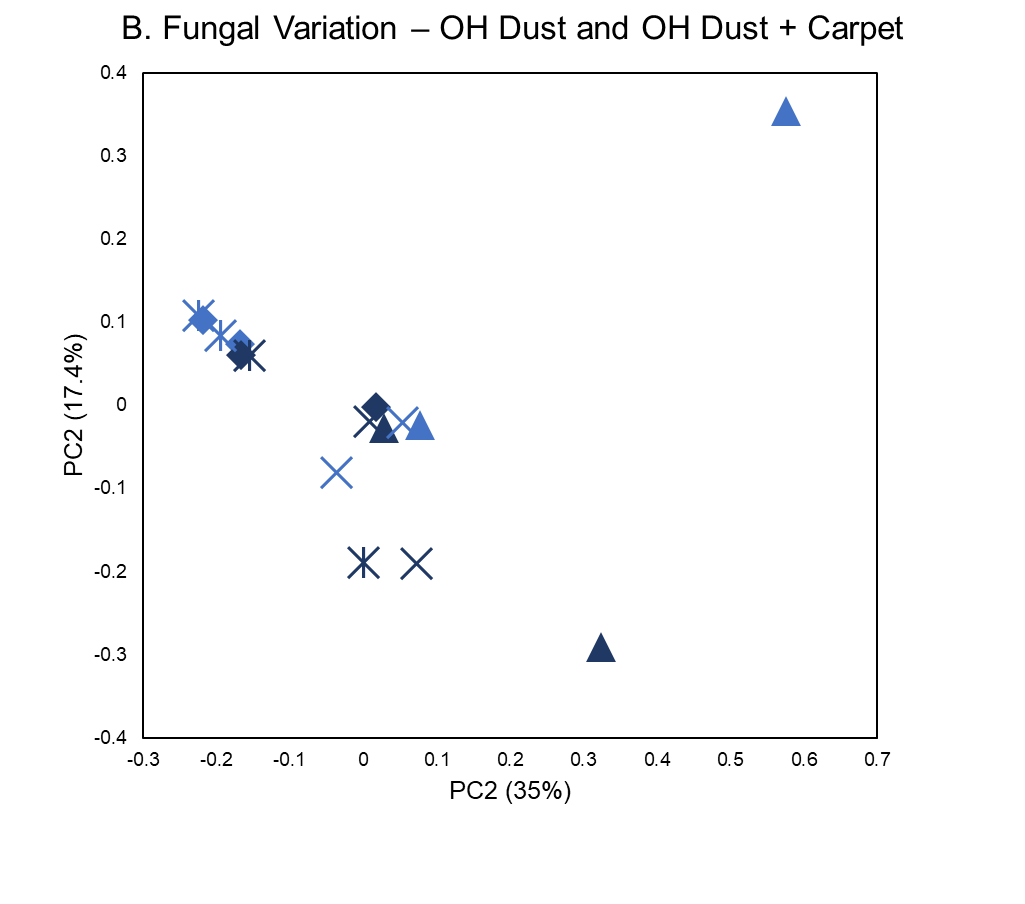

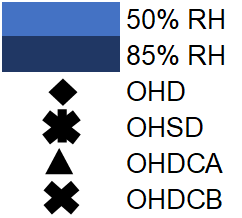

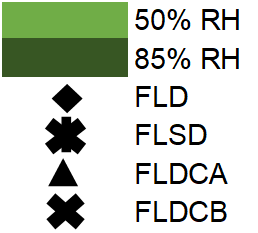

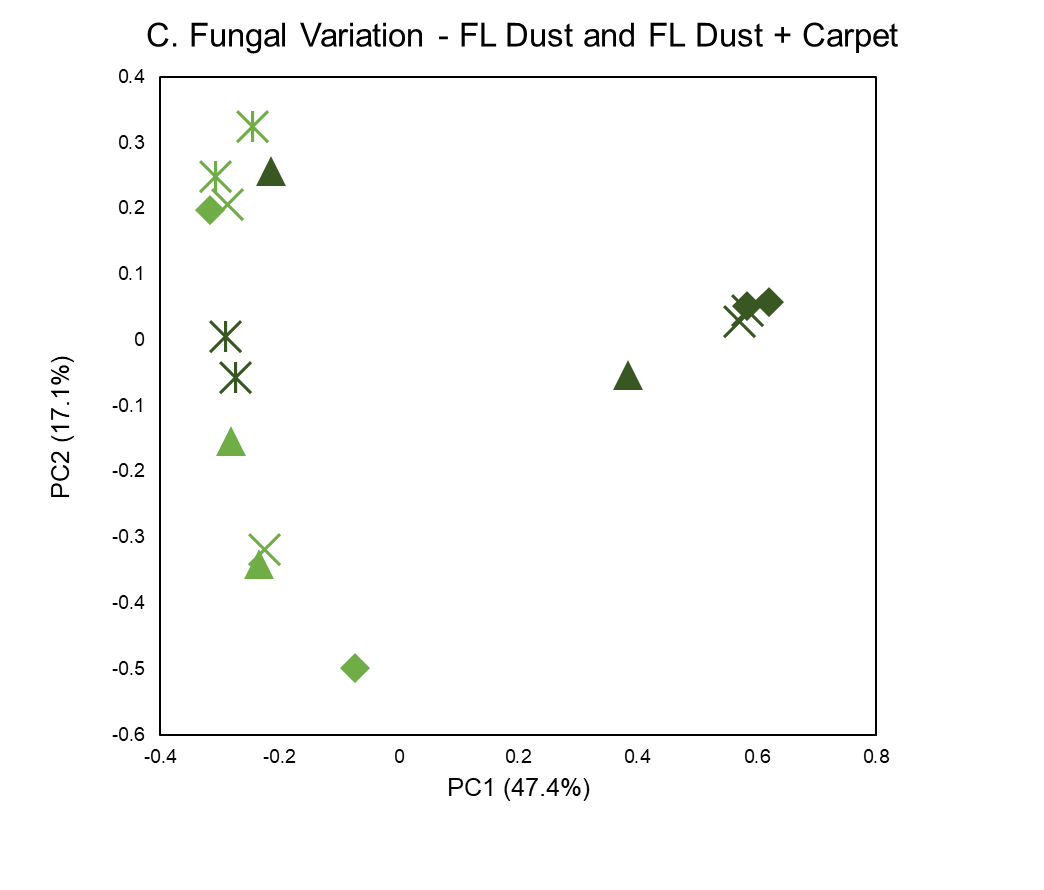

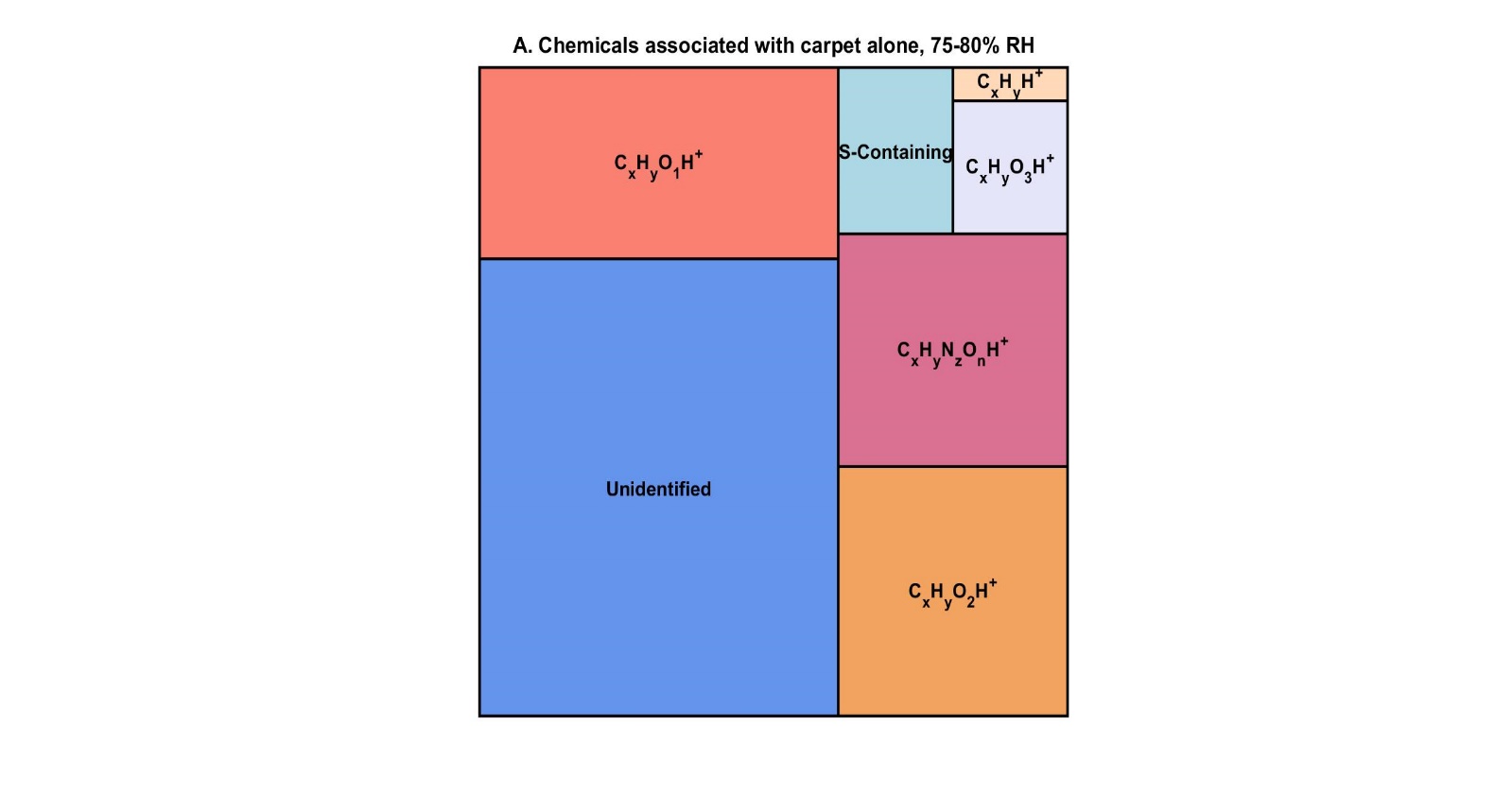


**Figure S14.** Proportion of chemical families emitted from the moisture availability carpet A samples, incubated at 75 and 80% ERH (A) and 95% ERH (B) for four weeks continuously. Measurements were taken over a period of around 2 hours.


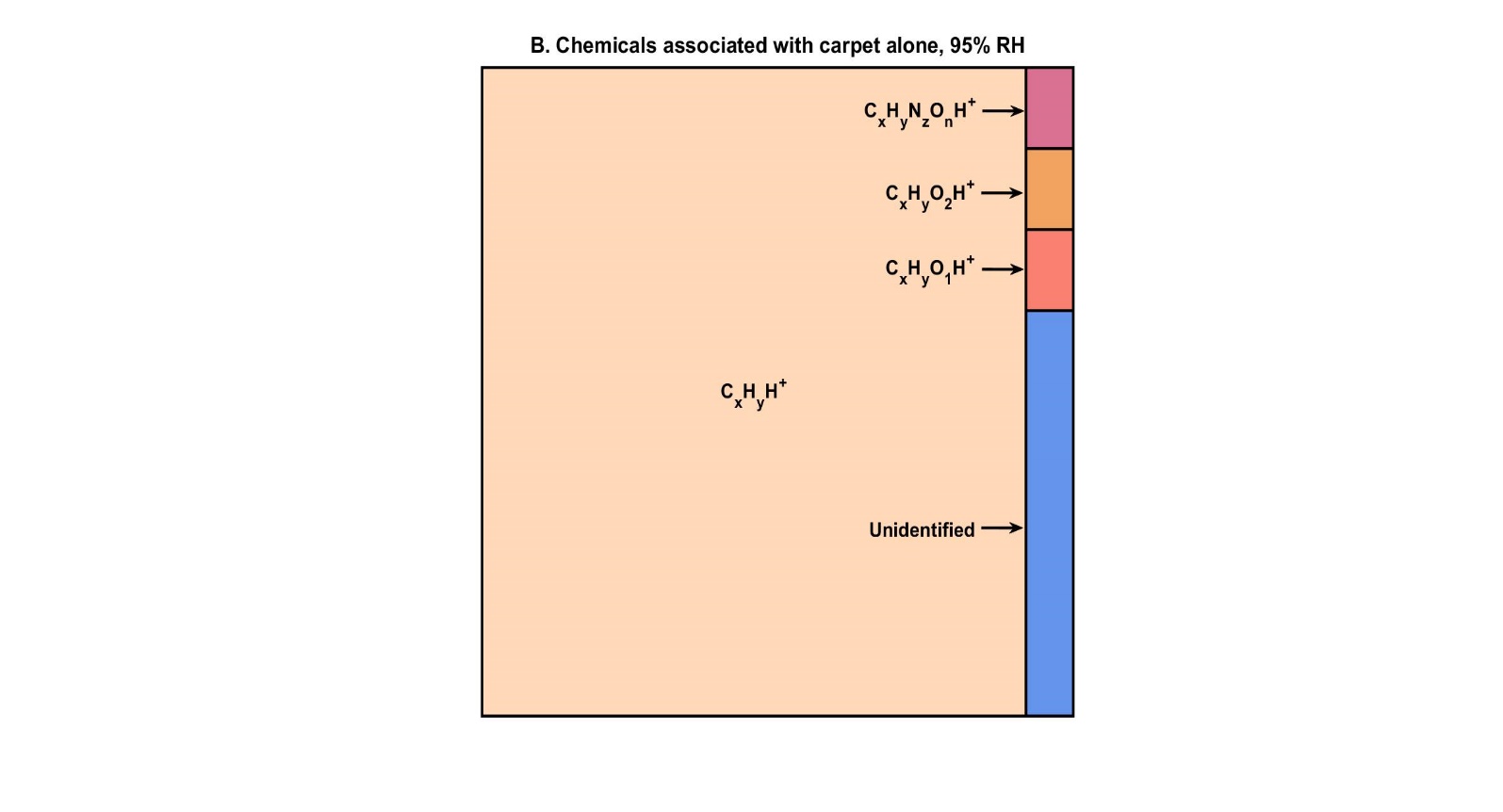

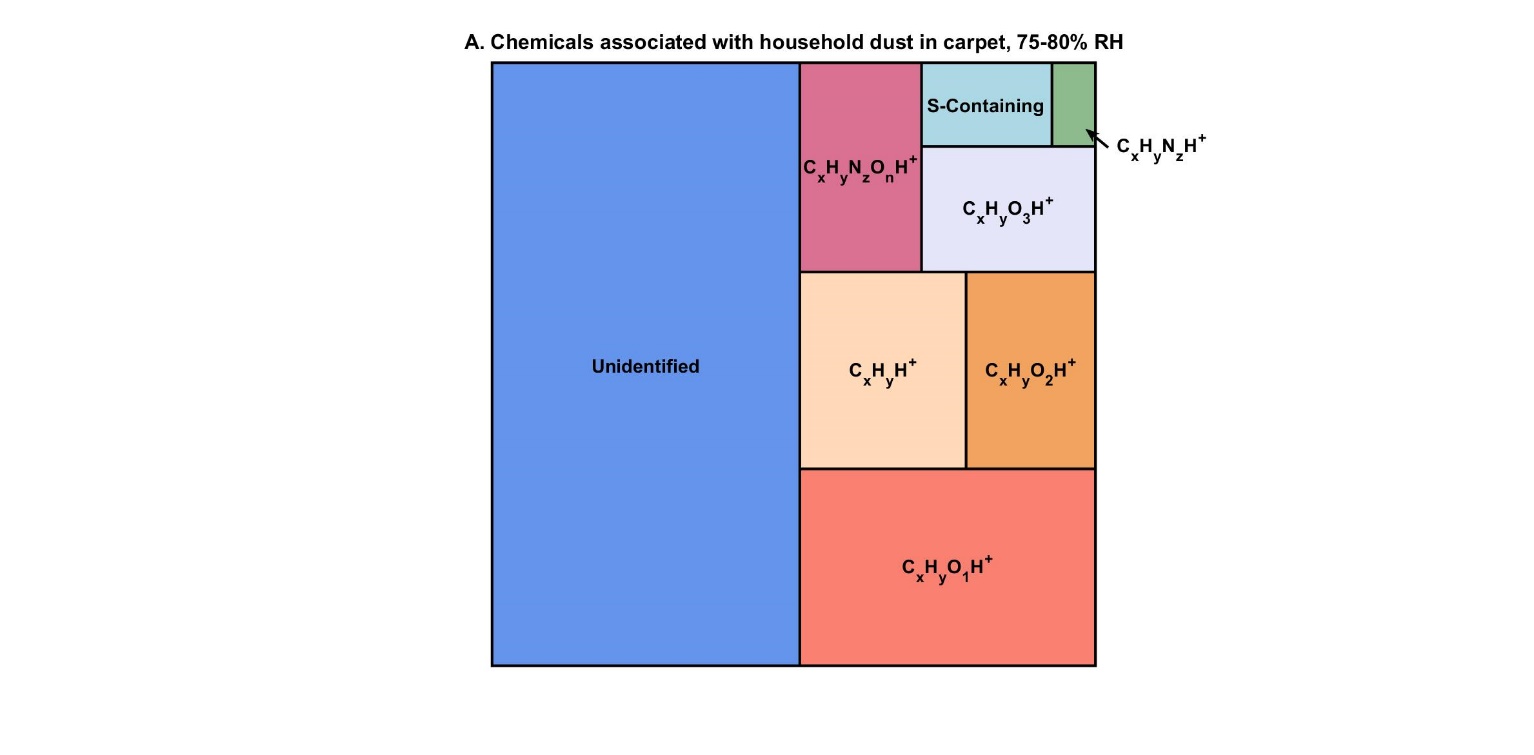


**Figure S15.** Proportion of chemical families emitted from San Francisco, CA carpet A with dust samples incubated at 75 and 80% ERH (A) and 95% ERH (B) for four weeks continuously. Measurements were taken over a period of around 2 hours.


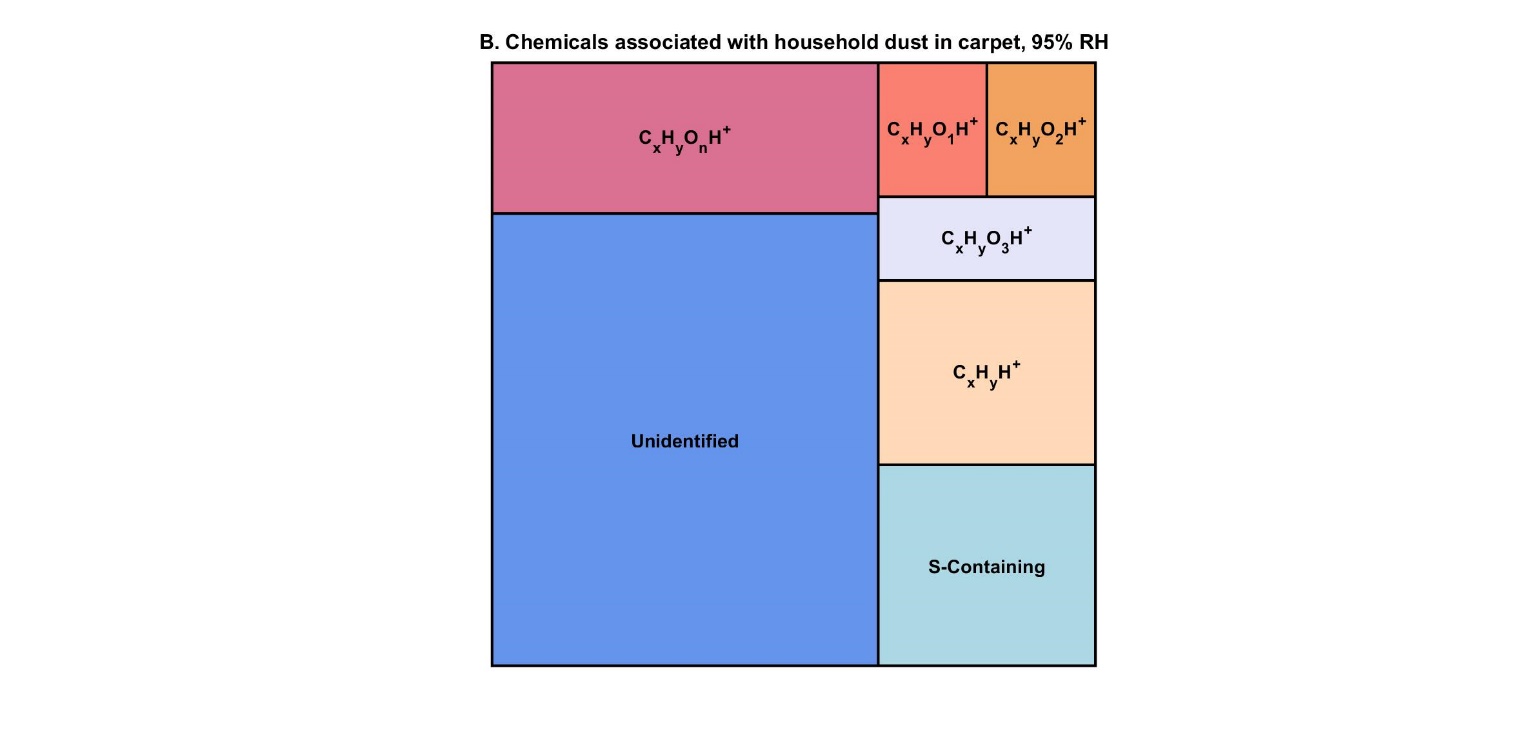


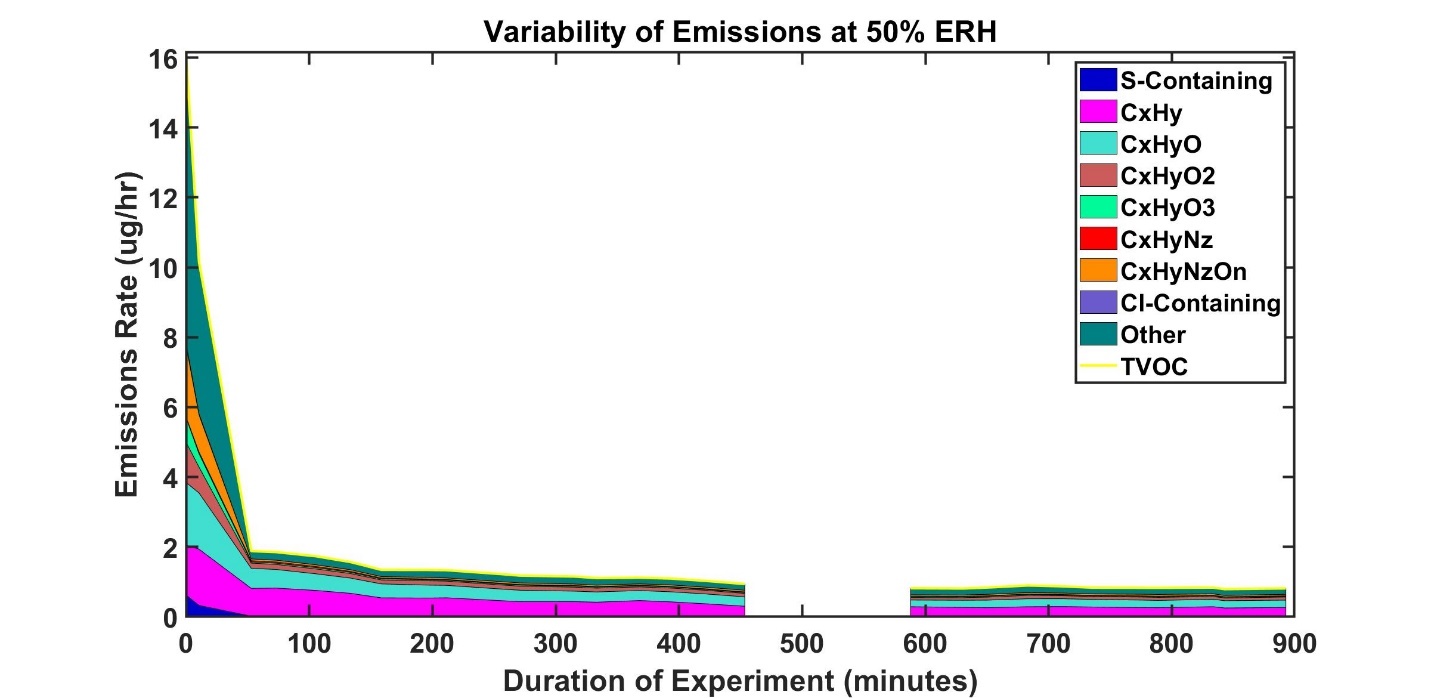


**Figure S16.** Emissions rate (µg hr^-1^) over sampling time of 900 minutes (15 hours). Samples include carpet with dust and carpet without dust samples incubated at 50% ERH from the mositure availability samples.

**
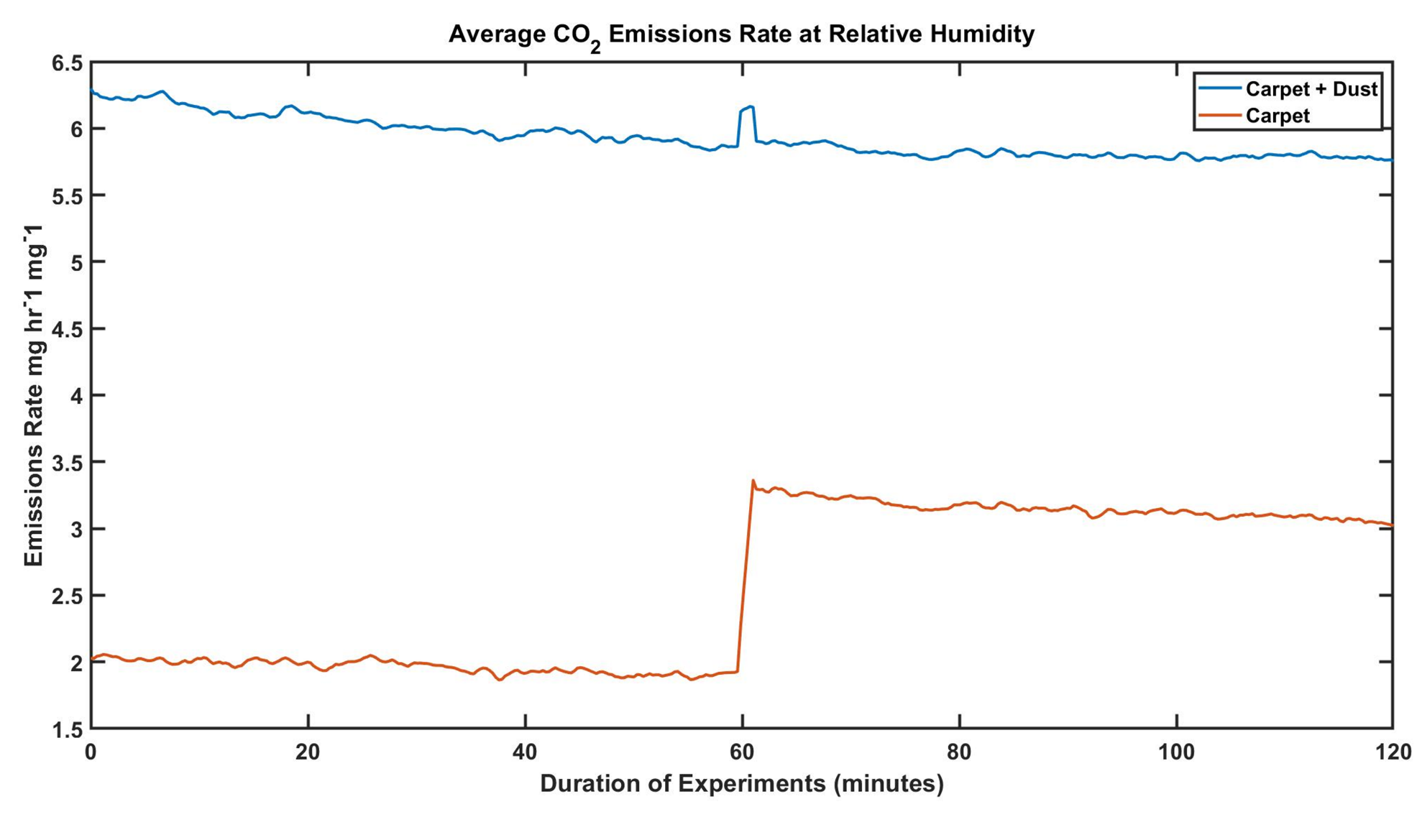
 Figure S17**. Carbon dioxide (CO_2_) emissions collected from moisture availability San Francisco, CA carpet A with dust and carpet A without dust incubated at 95% ERH. A recalibration of the instrument occurred at 60 minutes resulting in the spike seen at this time point.


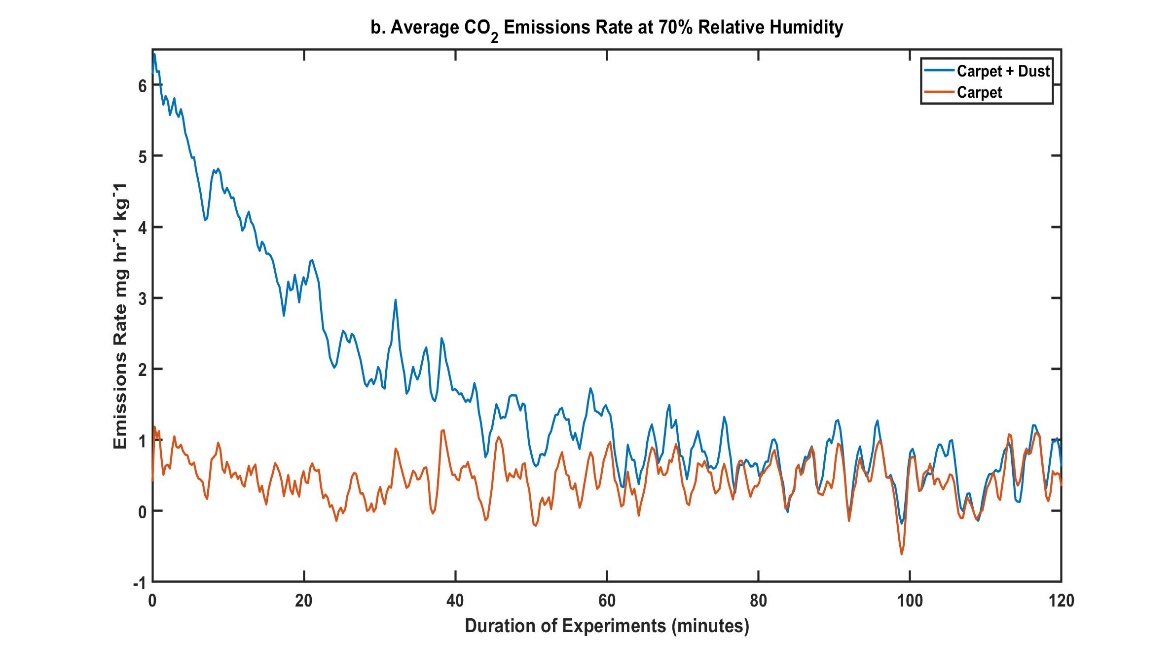

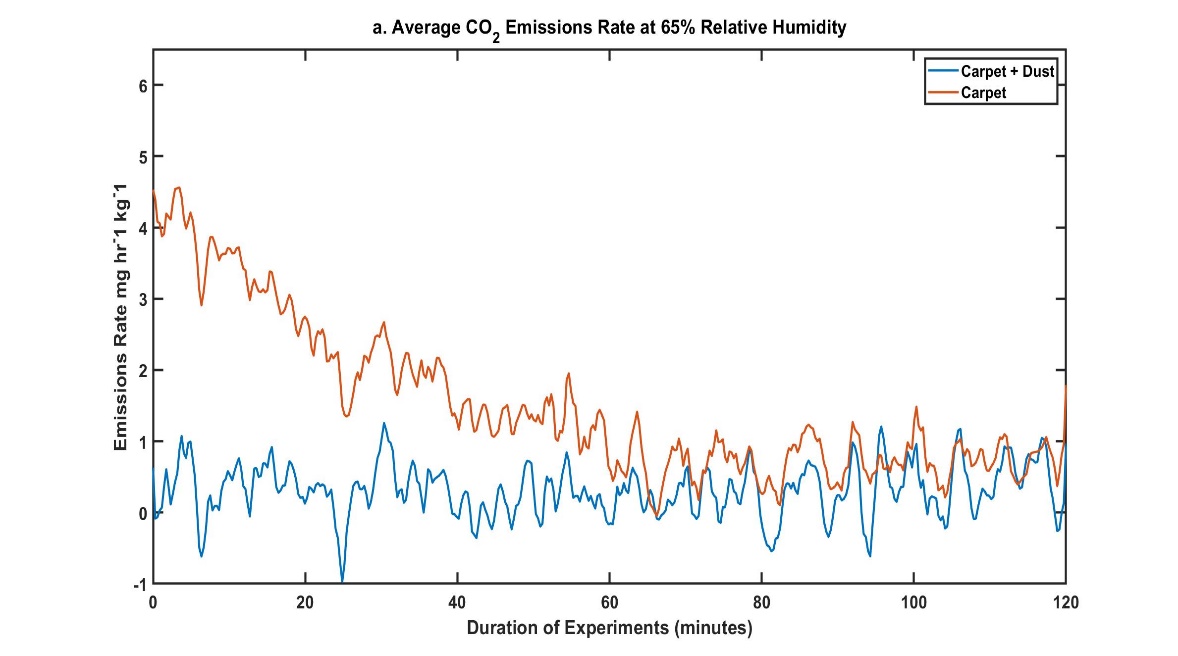

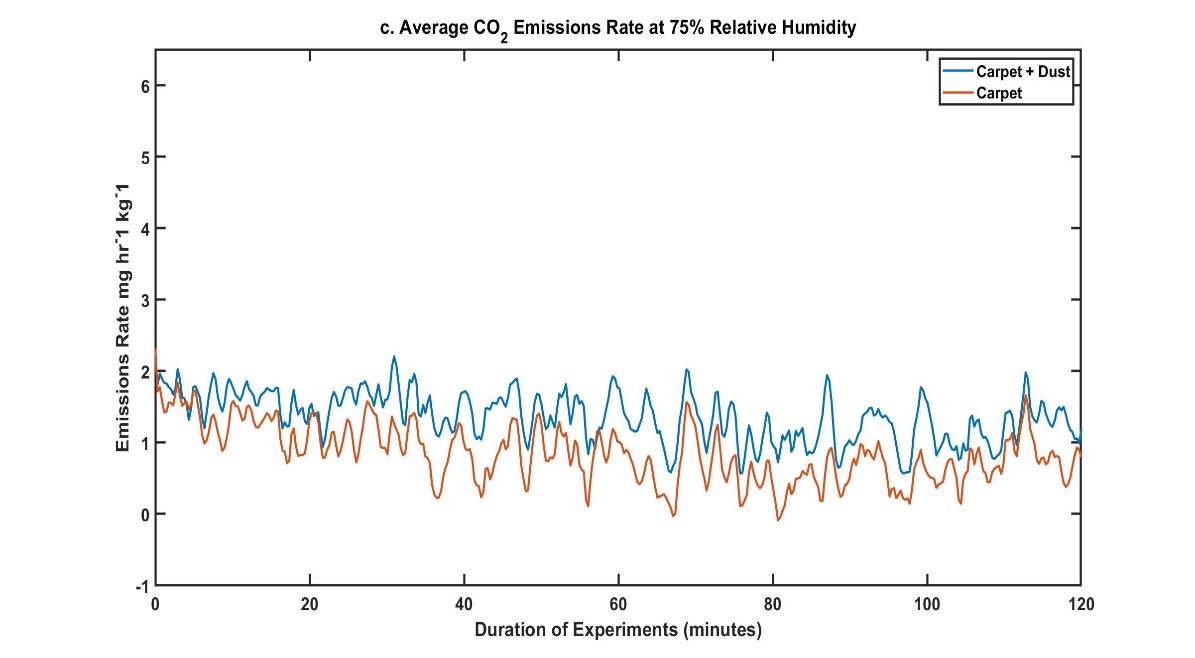


**Figure S18**. Carbon dioxide (CO_2_) emissions collected from moisture availability San Francisco, CA carpet A with dust and carpet A without dust samples incubated at 65% (A), 70% (B) and 75% (C).

**
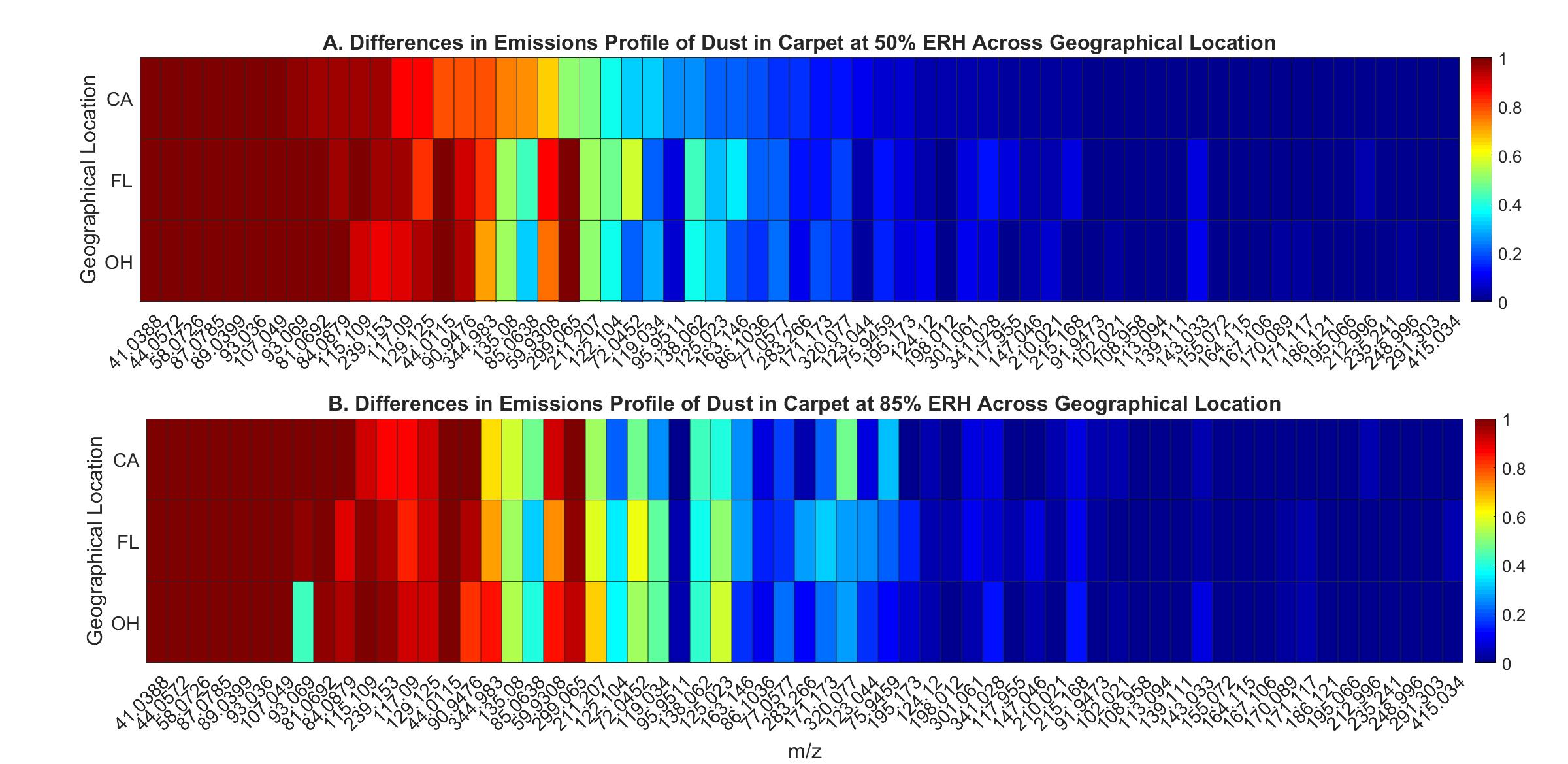
 Figure S19.** Heatmap displaying presence/absence of m/z ratios associated with carpet with dust at the medium relative humidity condition found in the moisture availability at each collection location San Francisco, CA, Gainesville, FL and Columbus, OH. mVOC profiles were similar between the emissions profile of carpet with dust at 50% ERH and 85% ERH across the collection sites.

**Figure S20.** Average number of fungal species determined from sequencing at each ERH in all the moisture availability samples. A linear regression was revealed with p=0.005 and R^2^=0.82 showing as the ERH increased the diversity of fungal species decreased.

**Table Headings**

All tables are located in the “Online_Supporting_Information_Tables.xlsm”. The table descriptions can be found below.

**Table S1.** A variety of statistical tests and comparisons were made for each sample set, moisture availability and collection site location. The statistical test, program, comparison, and variables used in the comparison can be found below.

**Table S2**. Fungal species associated with San Francisco, CA carpet and dust samples incubated at the High (95%) ERH condition for four weeks (moisture availability samples), using only fungal species found in ≥20% of all samples. Values not listed indicate that species was not significant (p<0.05) for that comparison.

**Table S3.** Fungal species associated with San Francisco, CA carpet and dust samples incubated at the Medium (75%-85%) ERH condition for four weeks (moisture availability samples), using only fungal species found in ≥20% of all samples. Values not listed indicate that species was not significant (p<0.05) for that comparison.

**Table S4.** Fungal species from the San Francisco, CA moisture availability carpet with dust samples associated with the Low (50% - 70%) ERH condition compared to the Medium (75%-85%) ERH and High (95%) ERH condition, using only fungal species found in ≥20% of all samples. Values not listed indicate that species was not significant (p<0.05) for that comparison.

**Table S5.** List of fungal species more associated with samples of painted drywall A from the moisture availability samples set at 95% ERH compared to samples set at 50%, 65%, 70%, 75%, 80% and 85% ERH. Utilizing only fungal species found in ≥20% of all samples. Values not listed indicate that species was not significant (p<0.05) for that comparison.

**Table S6.** List of fungal species more associated with samples of inoculated painted drywall A samples from the moisture availability experiments, set at 85% ERH compared to samples set at 50%-80% ERH or 95% ERH. Utilizing only fungal species found in ≥20% of all samples. Values not listed indicate that species was not significant (p<0.05) for that comparison.

**Table S7.** List of bacterial species more associated with samples of San Francisco, CA carpet with dust incubated at 50%-65%, 70%-75% and 80%-85% ERH when compared to samples set at 95% ERH continuously. Significance was determined as p<0.05.

**Table S8.** List of the 15 most abundant bacterial species found in the inoculated painted drywall samples from the moisture availability data set at each ERH condition. Quantities are an average of the triplicate samples for each sample condition.

**Table S9.** Fungal species associated with samples of carpet without dust, carpet with dust, painted drywall and inoculated painted drywall from San Francisco, CA when compared to samples from Columbus, OH and Gainesville, FL. These samples were part of the collection site location experiments in which samples were incubated at either 50% or 85% ERH continuously for four weeks. Significance was determined as p<0.05.

**Table S10.** Fungal species associated with samples of carpet A, carpet A with dust, painted drywall A and inoculated painted drywall A from Gainesville, FL when compared to samples from Columbus, OH and San Francisco, CA. These samples were part of the collection site location experiments in which samples were incubated at either 50% or 85% ERH continuously for four weeks. Significance was determined as p<0.05.

**Table S11.** Fungal species associated with samples of carpet A without dust, carpet A with dust autoclaved drywall A and inoculated drywall from Columbus, OH when compared to samples from Gainesville, FL and San Francisco, CA. These samples were part of the collection location experiments in which samples were incubated at either 50% or 85% ERH continuously for four weeks. Significance was determined as p<0.05.

**Table S12**. Results from the adonis comparisons from each sample set, moisture availability and collection location. Comparisons were made regarding sample type, carpet, carpet with dust and painted drywall as well as sample collection location.

**Table S13.** Fungal species associated with either carpet samples (A and B) or drywall samples (A and B) when compared from the collection location samples. All samples of carpet and drywall were utilized (carpet with dust, carpet without dust, inoculated painted drywall and autoclaved drywall) collected from each location.

**Table S14.** Fungal species detected in the Florida dust, Florida irradiated dust, Florida dust embedded in carpet A and Florida dust embedded in carpet B that were more associated with 50% ERH than 85% ERH condition. These samples were incubated at either 50% or 85% ERH for four weeks.

**Table S15.** M/z ratios more associated with the carpet A without dust incubated at 95% ERH than the carpet A with dust incubated at 95% ERH from moisture availability data set. All formulas and compounds are tentative identifications.

**Table S16.** M/z ratios more associated with the autoclaved drywall incubated at 95% ERH than the inoculated drywall incubated at 95% ERH from moisture availability data set. All formulas and compounds are tentative identifications.

**Table S17.** M/z ratios more associated with the inoculated drywall incubated at 95% ERH than the inoculated drywall incubated at 50-80% ERH from moisture availability data set. All formulas and compounds are tentative identifications.

**Table S18.** M/z ratios more associated with the inoculated drywall incubated at 95% ERH than the inoculated drywall incubated at 85% ERH from moisture availability data set. All formulas and compounds are tentative identifications.

**Table S19.** M/z ratios more associated with carpet with dust incubated at 75%-80% ERH when compared to carpet without dust incubated at 75-80% ERH from moisture availability data set. All formulas and compounds are tentative identifications.

**References**

1. Zhou G, Whong WZ, Ong T, Chen B. Development of a fungus-specific PCR assay for detecting low-level fungi in an indoor environment. Mol Cell Probes. 2000;14:339–48.

2. Nadkarni M, Martin FE, Jacques NA, Hunter N. Determination of bacterial load by real-time PCR using a broad range (universal) probe and primer set. Microbiology. 2002;148:257–66.

3. Qian J, Hospodsky D, Yamamoto N, Nazaroff WW, Peccia J. Size-resolved emission rates of airborne bacteria and fungi in an occupied classroom. Indoor Air. 2012;22:339–51.

4. Haines SR, Siegel JA, Dannemiller KC. Modeling microbial growth in carpet dust exposed to diurnal variations in relative humidity using the “Time‐of‐Wetness” framework. Indoor Air [Internet]. Blackwell Munksgaard; 2020 [cited 2021 Jan 26];30:978–92. Available from: https://onlinelibrary.wiley.com/doi/abs/10.1111/ina.12686

5. Bergmann GT, Bates ST, Eilers KG, Lauber CL, Caporaso JG, Walters WA, et al. The under-recognized dominance of Verrucomicrobia in soil bacterial communities. Soil Biol Biochem [Internet]. Elsevier Ltd; 2011;43:1450–5. Available from: http://dx.doi.org/10.1016/j.soilbio.2011.03.012

6. Schoch CL, Seifert K a., Huhndorf S, Robert V, Spouge JL, Levesque C a., et al. Nuclear ribosomal internal transcribed spacer (ITS) region as a universal DNA barcode marker for Fungi. Proc Natl Acad Sci U S A [Internet]. 2012;109:1–6. Available from: http://www.pnas.org/cgi/doi/10.1073/pnas.1117018109

7. Caporaso JG, Kuczynski J, Stombaugh J, Bittinger K, Bushman FD, Costello EK, et al. QIIME allows analysis of high-throughput community sequencing data. Nat Methods. 2010;7:335–6.

8. Altschul SF, Gish W, Miller W, Myers EW, Lipman DJ. Basic local alignment search tool. J Mol Biol. 1990;215:403–10.

9. Abarenkov K, Nilsson RH, Larsson KH, Alexander IJ, Eberhardt U, Erland S, et al. The UNITE database for molecular identification of fungi - recent updates and future perspectives. New Phytol. 2010;186:281–5.

10. Dannemiller KC, Reeves D, Bibby K, Yamamoto N, Peccia J. Fungal High-throughput Taxonomic Identification tool for use with Next-Generation Sequencing (FHiTINGS). J Basic Microbiol. 2014;54:315–21.

11. McDonald D, Price MN, Goodrich J, Nawrocki EP, Desantis TZ, Probst A, et al. An improved Greengenes taxonomy with explicit ranks for ecological and evolutionary analyses of bacteria and archaea. ISME J. 2012;6:610–8.

12. Jordan A, Haidacher S, Hanel G, Hartungen E, Märk L, Seehauser H, et al. A high resolution and high sensitivity proton-transfer-reaction time-of-flight mass spectrometer (PTR-TOF-MS). Int J Mass Spectrom. Elsevier; 2009;286:122–8.

13. Holzinger R. PTRwid: A new widget tool for processing PTR-TOF-MS data. Atmos Meas Tech. 2015;

14. Misztal PK, Lymperopoulou DS, Adams RI, Scott RA, Lindow SE, Bruns T, et al. Emission Factors of Microbial Volatile Organic Compounds from Environmental Bacteria and Fungi. 2018;52:8272–82.

15. Cappellin L, Karl T, Probst M, Ismailova O, Winkler PM, Soukoulis C, et al. On quantitative determination of volatile organic compound concentrations using proton transfer reaction time-of-flight mass spectrometry. Environ Sci Technol [Internet]. American Chemical Society; 2012 [cited 2020 Oct 20];46:2283–90. Available from: https://pubs.acs.org/doi/abs/10.1021/es203985t

16. Park JH, Goldstein AH, Timkovsky J, Fares S, Weber R, Karlik J, et al. Active atmosphere-ecosystem exchange of the vast majority of detected volatile organic compounds. Science (80- ). 2013;

17. Isaacman-Vanwertz G, Massoli P, O’Brien R, Lim C, Franklin JP, Moss JA, et al. Chemical evolution of atmospheric organic carbon over multiple generations of oxidation. Nat Chem. 2018;

18. Graus M, Müller M, Hansel A. High resolution PTR-TOF: Quantification and Formula Confirmation of VOC in Real Time. J Am Soc Mass Spectrom. No longer published by Elsevier; 2010;21:1037–44.

19. Lemfack MC, Gohlke BO, Toguem SMT, Preissner S, Piechulla B, Preissner R. MVOC 2.0: A database of microbial volatiles. Nucleic Acids Res. Oxford University Press; 2018;46:D1261–5.
